# Supplementary material for: How valid are projections of the future prevalence of diabetes? Rapid reviews of prevalence-based and Markov chain models and comparisons of different models’ projections for England
Source: BMJ Open. 2020 Mar 3;10(3):e033483. doi: 10.1136/bmjopen-2019-033483 (PMC7059487; doi:10.1136/bmjopen-2019-033483)
Supplement: Supplementary data [file bmjopen-2019-033483supp001.pdf]

Appendix 1: Tables giving details of models

Index

Table 1.1: Search strategy of the rapid reviews

Table 1.2: Measures of intermediate hyperglycaemia used in Markov chain models

Table 1.3: Data sources of estimates used by our Markov Chain models

Table 1.4: Ratios used for comparing different estimates

Table 1.5: The three sets of transition probabilities used in different models

Bevan G*, et al. BMJ Open* 2020; 10:e033483. doi: 10.1136/bmjopen-2019-033483

Table 1.1: Search strategy of the rapid reviews

**Epidemiological models**	
**Web of Science**	from All Databases<br>You searched for: TOPIC: ("diabet*" OR "type 2 diabetes" OR "diabetes mellitus") AND TITLE: ("Engl*" or "United Kingdom" or "UK") AND TOPIC: ("model" or "simulation" or "project*") AND TOPIC: ("epidemiolog*" or "prevalence" or "incidence" or "trend*") NOT TITLE: ("child*")<br>Refined by: LANGUAGES: (ENGLISH)<br>Timespan: All years.<br>Search language=Auto
**PubMed**	(((((("diabet*" OR "type 2 diabetes" OR "diabetes mellitus")) AND ("Engl*" OR "UK" OR "United Kingdom")) AND ("model" OR "simulation" OR "project*")) AND ("epidemiolog*" OR "prevalence" OR "incidence" OR "trend*")) NOT "child*" AND Humans[Mesh]) AND Humans[Mesh] AND English[lang] AND (Humans[Mesh] AND English[lang])
**Markov chain models**	
**Web of science**	TITLE: ("diabet*" OR "type 2 diabetes" OR "diabetes mellitus" or "pre-diabetes" or "prediabetes") AND TITLE: ("economic evaluation" or "cost-effectiveness" or "cost effectiveness" or "cost-utility" or "cost utility") AND TOPIC: ("Markov") NOT TOPIC: ("child*")<br>Refined by: LANGUAGES: (ENGLISH)<br>Timespan: All years.<br>Search language=Auto
**PubMed**	**((("diabet*" OR "type 2 diabetes" OR "diabetes mellitus" OR "prediabetes" OR "pre-**diabetes") AND ("economic evaluation" OR "cost-effectiveness" OR "cost effectiveness" OR "cost-utility" OR "cost utility")) AND "Markov" NOT "child") AND ("humans"[MeSH Terms] AND English[lang])

Table 1.2: Measures of intermediate hyperglycaemia used in Markov chain models

Measure of intermediate hyperglycaemia	Definition
Impaired Fasting Glucose (IFG)	• Diagnosed with an Oral Glucose Tolerance Test (OGTT) performed after an overnight fast<br>• Defined by a Fasting Plasma Glucose (FPG) concentration of<br>○ 5.6-6.9 mmol/L according to the American Diabetes Association (ADA)[1]<br>○ 6.0-6.9 mmol/L according to the World Health Organization (WHO)[2]
Impaired Glucose Tolerance (IGT)	• Diagnosed with a 2-hour Glucose Tolerance Test (2hrGTT), i.e. a blood test performed 2 hours after a 75-g glucose load<br>• Defined by 2-h plasma glucose concentration of<br>○ 7.8-11 mmol/L according to the American Diabetes Association (ADA)[1]<br>○ 7-11 mmol/L according to the World Health Organization (WHO)[2]
Glycated Haemoglobin (HbA1c)	• Diagnosed with the HbA1c test, measuring the average blood glucose over 2-3 months<br>• Defined by HbA1c concentration of<br>○ 39-47 mmol/mol (5.7-6.4%) according to the American Diabetes Association (ADA)[1]<br>○ 42-47 mmol/mol (6.0-6.4%) according to the World Health Organization (WHO)[3]

Bevan G, *et al. BMJ Open* 2020; 10:e033483. doi: 10.1136/bmjopen-2019-033483

Table 1.3: Data sources of estimates used by our Markov Chain models

Estimate	Year(s)	Source
Estimated prevalence of intermediate hyperglycaemia (based on HbA1c)	2015	Public Health England[4]
Estimated prevalence of diabetes (both types)	2015	Public Health England[5]
Estimated prevalence of normoglycaemia: residual of the population for 2015	2015	Office of National Statistics[6]
Age distributions for those with intermediate hyperglycaemia & diabetes	Five years of combined data from 2009 to 2013	Health Surveys for England (HSE)[7]
Mortality rates by age	2015	Office of National Statistics[6]
Hazard ratios for those with diabetes & T2D	2015-16	National Diabetes Audit[8]
Hazard ratios for those for those with intermediate hyperglycaemia	Various years	Systematic review[9]

Bevan G, *et al*. *BMJ Open* 2020; 10:e033483. doi: 10.1136/bmjopen-2019-033483

Table 1.4: Ratios comparing different estimates

Numerator	Denominator	Ratio	Sources
Diagnosed prevalence of diabetes	True prevalence of diabetes	75%	[10,11]
Prevalence of T2D	Prevalence of diabetes	90%	[12]
Prevalence of diabetics aged over 80 (England) in 2015, 2030 & 2035	Prevalence of diabetics aged 20 to 79 (England) in 2030 & 2035	• 2015: 18%<br>• 2030: 26%<br>• 2035: 29%	[6, 7]

Table 1.5: The three sets of transition probabilities used in different models

	Model 1*	Model 2**	Model 3***
Normoglycaemia – Normoglycaemia	0.925	0.925	0.831
Normoglycaemia – Intermediate hyperglycaemia	0.069	0.069	0.163
Normoglycaemia – T2D	0.000	0.000	0.000
Normoglycaemia – Dead	0.006	0.006	0.006
Totals	*1.000*	*1.000*	*1.000*
Intermediate hyperglycaemia -Intermediate hyperglycaemia	0.856	0.878	0.755
Intermediate hyperglycaemia- Normoglycaemia	0.090	0.090	0.162
Intermediate hyperglycaemia – T2D	0.036	0.013	0.060
Intermediate hyperglycaemia – Dead	0.019	0.019	0.023
Totals	*1.000*	*1.000*	*1.000*
T2D-T2D	0.977	0.977	0.973
T2D – Normoglycaemia	0.000	0.000	0.000
T2D- Intermediate hyperglycaemia	0.000	0.000	0.005
T2D – Dead	0.023	0.023	0.022
Totals	*1.000*	*1.000*	*1.000*

Notes:
* Model 1is based on the transition probabilities from Roberts et al[14]for HbA1c.
** Model 2 is based on Model 1 modified to generate the PHE projections of the prevalence of T2D:the transition probability from intermediate hyperglycaemia to T2D of Model 2 (0.013) is a third of that of Model 1 (0.036); and has a corresponding increase in the transition probability of remaining as intermediate hyperglycaemia (0.836 to 0.878).
*** Model 3 is based on the transition probabilities from Neuman et al[15] for IGT.

References

1 American Diabetes Association (ADA). 2. Classification and Diagnosis of Diabetes: Standards of Medical Care in Diabetes. *Diabetes Care* 2018;**41**:S13–27.

2 World Health Organization (WHO). Definition and diagnosis of diabetes mellitus and intermediate hyperglycemia : report of a WHO/IDF consultation. Geneva (CH): 2006.

3 World Health Organization (WHO). Use of glycated haemoglobin (HbA1c) in the diagnosis of diabetes mellitus. 2011.

4 Public Health England - National Cardiovascular Intelligence Network. Prevalence estimates of non-diabetic hyperglycaemia. 2015.

5 National Cardiovascular Intelligence Network (NCVIN) - Public Health England. Prevalence estimates of diabetes. 2016.

6 Office for National Statistics. 2014-based National Population Projections. 2015.

7 National Cardiovascular Intelligence Network (NCVIN)- Public Health England. NHS Diabetes Prevention Programme (NHS DPP) Non-diabetic hyperglycaemia. 2015.

8 Healthcare Quality Improvement Partnership (HQIP). National Diabetes Audit, 2015-16 Report 2: Complications and Mortality. 2017.

9 Huang Y, Cai X, Mai W, *et al.* Association between prediabetes and risk of cardiovascular disease and all cause mortality: systematic review and

meta-analysis. *BMJ* 2016;**355**:i5953. doi:10.1136/bmj.i5953

10 Holman N, Forouhi NG, Goyder E, *et al.* The Association of Public Health Observatories (APHO) Diabetes Prevalence Model: Estimates of total diabetes prevalence for England, 2010-2030. *Diabet Med* 2011;**28**:575–82. doi:10.1111/j.1464-5491.2010.03216.x

11 Moody A, Cowley G, Fat LN, *et al.* Social inequalities in prevalence of diagnosed and undiagnosed diabetes and impaired glucose regulation in participants in the Health Surveys for England series. *BMJ Open* 2016;**6**:e010155. doi:10.1136/BMJOPEN-2015-010155

12 Gatineau M, Hancock C, Holman N, *et al.* Adult obesity and type 2 diabetes. London, UK: 2014.

13 Shaw JEE, Sicree RAA, Zimmet PZZ. Global estimates of the prevalence of diabetes for 2010 and 2030. *Diabetes Res Clin Pract* 2010;**87**:4–14. doi:10.1016/j.diabres.2009.10.007

14 Roberts S, Craig D, Adler A, *et al.* Economic evaluation of type 2 diabetes prevention programmes: Markov model of low- and high-intensity lifestyle programmes and metformin in participants with different categories of intermediate hyperglycaemia. *BMC Med* 2018;**16**:16. doi:10.1186/s12916-017-0984-4

15 Neumann A, Lindholm L, Norberg M, *et al.* The cost-effectiveness of interventions targeting lifestyle change for the prevention of diabetes in a Swedish primary care and community based prevention program. *Eur J Heal Econ* 2017;**18**:905–19. doi:10.1007/s10198-016-0851-9
