# Supplementary material for: How valid are projections of the future prevalence of diabetes? Rapid reviews of prevalence-based and Markov chain models and comparisons of different models’ projections for England
Source: BMJ Open. 2020 Mar 3;10(3):e033483. doi: 10.1136/bmjopen-2019-033483 (PMC7059487; doi:10.1136/bmjopen-2019-033483)
Supplement: Supplementary data [file bmjopen-2019-033483supp002.pdf]

Appendix 2: Rapid Reviews of Epidemiological & Markov chain models

Table 2.1 gives the search strategies for the review & Table 2.2 gives details of our rapid review of Markov chain models.

Table 2.1: Search strategies

**Web of science**	TITLE: ("diabet*" OR "type 2 diabetes" OR "diabetes mellitus" or "pre-diabetes" or "prediabetes") & TITLE: ("economic evaluation" or "cost-effectiveness" or "cost effectiveness" or "cost-utility" or "cost utility") NOT TOPIC: ("child*" or "pediatric" or "paediatric") NOT TOPIC: ("type 1 diabetes") & TOPIC: ("markov")<br>Refined by: LANGUAGES: (ENGLISH)<br>Timespan: All years.<br>Search language=Auto
**PubMed**	(((("diabet*"[All Fields] OR "type 2 diabetes"[All Fields] OR "diabetes mellitus"[All Fields] OR "pre-diabetes"[All Fields] OR "prediabetes"[All Fields]) & ("economic evaluation"[All Fields] OR "cost-effectiveness"[All Fields] OR "cost effectiveness"[All Fields] OR "cost-utility"[All Fields] OR "cost utility"[All Fields])) & "markov"[All Fields]) NOT ("child*"[All Fields] OR "pediatric"[All Fields] OR "paediatric"[All Fields])) NOT "type 1 diabetes"[All Fields] & ("humans"[MeSH Terms] & English[lang])

Bevan G, *et al. BMJ Open* 2020; 10:e033483. doi: 10.1136/bmjopen-2019-033483

Table 2.2: Details of our rapid review of Markov chain models

Author	Country	Risk measure *	Objectives	Model description	Population modelled	Outcomes	Number of cases with no intervention	Sensitivity analysis	Model validation
Caro et al, 2004 [1]	Canada	IGT	To compare health & economic outcomes of acarbose, an intensive lifestyle modification programme, metformin or no intervention to prevent progression to diabetes	A Markov model to simulate long-term outcomes in a cohort of patients with IH under each of four treatment strategies. The cohort is followed for a 10- year period in the base case analyses. The model cycles over 6-month periods. Four main states were considered: normoglycaemia (NG), intermediate hyperglycaemia (IH) Type 2 Diabetes (T2D) & death. Patients who revert to NG may develop IH again, while patients who develop diabetes are assumed to remain in that state until death.	Cohort of patients with IH. For base case, patient characteristics were taken from STOP-NIDDM trial. Just over half of patients in that trial were male, & mean age at start of the trial was 54.5 years	No of patients transitioning to T2D<br>No who reverted & remained NG<br>Life expectancy<br>Years free of T2D	For a cohort of 1000 patients, over course of 10 years, 542 untreated patients with IH are expected to develop diabetes, while 242 will have returned to NG	Performed, results for base case not reported	Not reported
Chen et al, 2001[2]	Taiwan	NA	To develop natural history of T2D<br>To quantify efficacy of early detection of T2D in slowing or reducing progression of complications<br>To evaluate effect of inter-screening interval & age at start of screening on slowing/reducing progression of complications or deaths<br>To compare cost & effectiveness of a screening regime<br>To assess cost–effectiveness of T2D screening by age-specific groups & different inter-screening interval	A Markov model to simulate natural history of T2D from normal, onset, clinical complications, deaths. Disease progression modules from onset of T2D to complications include three parts: Retinopathy, Nephropathy, & Neuropathy.	Hypothetical cohort with 30,000 adults aged over 30	Life-years gained<br>QALYs	Not reported	Not reported	Not reported
Gillies et al, 2008[3]	UK	IGT	To compare potential screening strategies, & subsequent interventions, for prevention & treatment of T2D<br>(a) screening for T2D to enable early detection & treatment<br>(b) screening for T2D & impaired glucose tolerance, intervening with lifestyle interventions in those with a diagnosis of impaired glucose tolerance<br>(c) as for (b) but with pharmacological interventions<br>(d) no screening	Hybrid model consists of a decision tree & a Markov model<br>The decision tree comprises three main arms, representing no screening, screening for undiagnosed T2D, & screening for impaired glucose tolerance & undiagnosed diabetes, with either lifestyle or pharmacological interventions applied in those with impaired glucose tolerance<br><br>The Markov model consists of seven states: normal glucose tolerance, undiagnosed impaired glucose tolerance, diagnosed impaired glucose tolerance, death, & three states for people with diabetes (undiagnosed, diagnosed clinically, or diagnosed through screening, either from a screening test or because they are diagnosed with impaired glucose tolerance initially & hence enter a surveillance programme)<br>Each model cycle represents one year & the model is run for a time horizon of 50 years.	Hypothetical population, aged 45 at time of screening, with above average risk of diabetes	Clinical & cost outcomes	Not reported	Performed, results reported	Not reported

Bevan G, *et al. BMJ Open* 2020; 10:e033483. doi: 10.1136/bmjopen-2019-033483

Author	Country	Risk measure *	Objectives	Model description	Population modelled	Outcomes	Number of cases with no intervention	Sensitivity analysis	Model validation
Herman et al, 2005[4]	USA	IGT	To estimate lifetime cost–utility of the DPP interventions.	Markov model assesses progression from IH to onset of diabetes to clinically diagnosed diabetes to diabetes with complications & death by using a lifetime simulation model. Description of the model reported elsewhere.	Members of the DPP cohort 25 years of age or older with impaired glucose tolerance	Progression of disease<br>Costs<br>Quality of life	If the entire DPP cohort were treated with the placebo intervention, approximately 50% of individuals would develop diabetes within 7 years. Over a lifetime conversion rate from IH to T2D is 82.8%	Performed, results reported	Not reported
Ikeda et al, 2010[5]	Japan	IGT	To estimate cost-effectiveness of administering voglibose, in addition to standard care of diet & exercise, compared with standard care alone for high-risk Japanese patients with impaired glucose tolerance	Markov model consisting of five stages: normal glucose tolerance, IH, T2D, dialysis & death	IH cohort, mean age 56, corresponding to the average age in the voglibose clinical trial population	Long-term costs<br>Life expectancy<br>Cost effectiveness	Not reported	Performed, results reported	Not reported
Johansson et al, 2009 [6]	Sweden	FPG	To estimate cost-effectiveness of a community-based program promoting general population lifestyle changes to prevent diabetes.	Markov model constructed to reflect metabolic syndrome, covers adults, with termination age set at 85 years, after which no further health effects or costs are accumulated. Model is fully described elsewhere.	At high risk population aged 36–56 years at baseline	Costs<br>QALYs	Not reported	Performed, results reported	Not reported
Liu et al, 2013[7]	China	IGT	To estimate clinical & economic outcomes of screening for undiagnosed diabetes & impaired glucose tolerance (IH), followed by the implementation of lifestyle intervention in those with IH.	Hybrid decision tree Markov model. The decision tree included five arms representing five scenarios. The first three scenarios involved screening for undiagnosed diabetes & IH followed by one of three active lifestyle interventions (diet, exercise or duo-intervention), which were applied to the IH subjects. The fourth scenario involved screening for undiagnosed diabetes & IH, without formal lifestyle interventions. The fifth scenario involved control group with no screening or intervention.<br>The decision tree used positive screening rates & the prevalence of diabetes & IH in reference population to determine how many individuals started in each state of the Markov models. Each Markov model consisted of eight main health states: IH, normal glucose tolerance, onset of diabetes, four diabetes complication states & death.<br>The Markov models ran for a time horizon of 40 years, & each of the model cycles represented 1 year. Separate simulations with different incidence rates of diabetes, mortality rates & health utilities were performed for the diabetes prevention programmes or for the control starting at 25, 40 & 60 years, respectively.	A representative sample of Chinese adults aged 25 years & above	Remaining survival years<br>QALYs per subject with diabetes or IH<br>Life-years gained before onset of diabetes or before onset of any complication per subject with IH<br>Cost per subject for prevention strategies or control at different initiation ages.	Not reported	Performed, results reported.	Performed, not reported
Neumann et al, 2011 [8]	Germany	IGT	To investigate long-term cost-effectiveness of lifestyle intervention programmes for the prevention of T2D	Four-state Markov modelling with a probabilistic cohort analysis : NG, IH, diagnosed T2D, or death.<br>A one-year cycle length & a lifetime time horizon are applied.	Cohort, at baseline 16% of individuals having IH, 84% NG & no one T2D.	Cost per quality-adjusted life year (QALY)	Not reported	Performed, results reported	Not reported
Neumann	Sweden	IFG	To estimate cost-	The model consisted of	With IH (details not	QALY	Not reported	Performed,	Not reported

3

Author	Country	Risk measure *	Objectives	Model description	Population modelled	Outcomes	Number of cases with no intervention	Sensitivity analysis	Model validation
et al, 2017 [9]		IGT	effectiveness of a T2D prevention initiative targeting weight reduction, increased physical activity & healthier diet in persons in pre-diabetic states by comparing a hypothetical intervention versus no intervention in a Swedish setting.	six different, mutually exclusive states: NG, IH (IGT & IGT), T2D & death. The length of one cycle was 1 year. A lifetime horizon was applied. As it was assumed that 1 year was too short to develop T2D directly from NG, this transition was not possible. Hence, all hypothetical persons must have developed any of the three pre-diabetic states before the development of T2D.	reported) based on the Vasterbotten Intervention Program (VIP)	Incremental cost-effectiveness ratios (ICERs)		results reported	
Palmer & Tucker, 2012 [10]	Australia	IGT	To examine long-term cost-effectiveness of the control, metformin & ILC interventions in the Diabetes Prevention Program (DPP) for a cohort of subjects at high risk of developing type 2 diabetes in an Australian healthcare setting	Semi-Markov model, with four health states: 'normal glucose regulation' (NGR) (plasma glucose con-centration <5.6 mmol/L in fasting state or <7.8 mmol/L 2 h after a 75 g oral glucose load); 'impaired glucose tolerance' (IH) (fasting plasma glucose concentration 5.6–6.9 mmol/L or 7.8–11.0 mmol/L 2 h after a 75 g oral glucose load); 'type 2 diabetes' (T2D) (plasma glucose concentration at least 7.0 mmol/L or 11.1 mmol/L 2 h after a 75 g oral glucose load),'dead'. Each cycle in the model represented one year of a simulated subject's life & at the end of each cycle, subjects could remain in the same state, progress to another state or die. The simulation ran over subject lifetimes	Hypothetical cohort was defined with baseline characteristics in keeping with DPP study: mean age 50.6 years; 32.2% male; mean body mass index 34.0 kg/m2; & IH present.	Cumulative incidence<br>Lifetime incremental direct costs<br>Incremental costs per QALY-gained	Mean cumulative incidence (95% CI) of type 2 diabetes in the control arm , estimated at 89.7% (89.4–90.1)	Performed, results reported	Validation performed against the observed incidence in the US DPP & follow-up DPPOS trials. R2 correlation-coefficient estimated at 0.9987
Palmer et at, 2004 [11]	Australia<br>France<br>Germany<br>Switzerland<br>UK	IGT	To establish whether implementing active treatments used in DPP would be cost-effective in the selected countries.	Markov model consisting of 3 states: IH (as defined in the DPP), T2D & deceased. Simulated patients initially had IH & progressed at differing rates to T2D depending on treatment received. A patient lifetime horizon was used.	Hypothetical cohort of patients with IH, constructed to resemble the study population of the DPP (mean age, 50.6 years; mean body weight, 94.2 kg; mean body mass index [BMI], 34.0 kg/m2; men, 32.2%)	No of years free of T2D<br>Percentage of patients developing T2D<br>Life expectancy<br>Total lifetime costs per patient	Not reported	Performed, results reported	Not reported
Roberts et al, 2018 [12]	England	IFG<br>IGT<br>HbA1c	To examine costs and effects of different intensity lifestyle programmes and metformin in participants with different categories of intermediate hyperglycaemia	Decision tree and Markov model (50-year horizon) to compare four approaches: (1) a low-intensity lifestyle programme based on current NICE guidance, (2) a high-intensity lifestyle programme based on the US Diabetes Prevention Program, (3) metformin, and (4) no intervention, modelled for three different types of intermediate hyperglycaemia (IFG, IGT and HbA1c).	Population with a diagnosis of intermediate hyperglycaemia (IFG, IGT, HbA1c)	Impact on an individual participant in a prevention programme: (1) discounted cumulative healthcare costs (including costs of diagnostic tests and primary and secondary care associated with the intervention, intermediate hyperglycaemia, T2DM and complications of T2DM), (2) discounted QALYs, (3) incidence of T2DM, (4) average number of years with T2DM, (5) cost-effectiveness ratios in £/QALY, and (6) incremental cost-effectiveness ratios (ICERs), in £/QALY (for non-dominated interventions).<br>Impact of a nation-wide prevention programme: (1) discounted annual incremental costs, (2) discounted	With no intervention, 42% of the IGT population and 38% of the IFG and HbA1c population developed T2DM over 50 years.	Performed, results available	Performed against the National Diabetes Audit 2015-2016. Reported for the prevalence of T2D by age groups (55-59, 60-64, 65-69, 70-74, 75-79, 80-84, 85+)

Author	Country	Risk measure *	Objectives	Model description	Population modelled	Outcomes	Number of cases with no intervention	Sensitivity analysis	Model validation
						cumulative incremental costs, (3) discounted incremental costs as a percentage of the total diabetes expenditure, and (4) cumulative incidence of T2DM.			
Schaufler & Wolfe, 2010[13]	Germany	OGTT	To examine cost effectiveness of screening for T2D in Germany	Markov model to reproduce the time-discrete stochastic process using a 1 year cycle	General German population	Quality of Life (QOL)<br>Lifetime costs<br>Age at diabetes diagnosis<br>Incidence & Age at occurrence of diabetes-related complications.	Not reported	Performed, results reported	Performed, results not reported
Smith et al, 2010[14]	USA	IFG	To assessed cost-effectiveness of a modified version of the US DPP (mDPP)	Markov model with six states: risk factor negative (no diabetes), risk factor positive (enrolled in mDPP), risk factor positive (not enrolled in mDPP), stable T2D, complications, death	Cohort of 55-year-old men & women without a history of diabetes	Metabolic syndrome risk at 1 year<br>Costs<br>QALYs<br>T2D incidence	Without the mDPP, 9.6% of the cohort developed diabetes over 3 years	Performed, results for base-case not reported	Not reported
Wong et al, 2016 [15]	Hong Kong	IGT	To investigate costs & cost-effectiveness of a short message service (SMS) intervention to prevent the onset of T2D with IH..	Markov model with one-year transition cycle with four Markov states: normal glucose tolerance (NG), IH, T2D, & death.<br>Long-term modelling referred to time horizon over a 50-year period beyond the two year intervention	Cohort of individuals with prediabetes	Costs<br>QALYs	Not reported	Performed, results reported	Not reported
Zhou et al. 2005[16]	USA	IGT	To develop & validate a comprehensive computer simulation model to assess the impact of screening, prevention, & treatment strategies on T2D & its complications, comorbidities, quality of life, & cost.	Markov model with four states: NG, IH, T2D, death.	Not described	Health states<br>Utilities<br>Costs	Not reported	Not reported	Performed against data on individuals with T2D in Wisconsin, USA) from the Wisconsin Epidemiologic Study of Diabetic Retinopathy (WESDR). Results not reported.
Zhuo et al, 2012 [17]	USA	HbA1c	To examine change in cost effectiveness of diabetes-preventive interventions because of progressive 0.1% decremental reductions in the HbA1c cutoff from 6.4% to 5.5%.	Markov model reported elsewhere.	Nationally representative sample of U.S. adults (aged 18 years) from the 1999–2006 National Health & Nutrition Examination Survey (NHANES)	Cost effectiveness associated with HbA1c cutoffs was measured as cost per QALY gained	Not reported	Performed, results reported	Performed against results of 47 major clinical trials & cohort studies. Results not reported. Details of the model's validation reported elsewhere

Notes:

* Risk measures: HbA1c: Glycated Haemoglobin; IFG: Impaired fasting glucose; IGT: Impaired glucose tolerance; OGTT: Oral glucose tolerance test; FPG: Fasting plasma glucose; NG: normoglycaemia

References

[1] Caro JJ, Getsios D, Caro I, et al. Economic evaluation of therapeutic interventions to prevent Type 2 diabetes in Canada. Diabet Med 2004;21:1229–36. doi:10.1111/j.1464-5491.2004.01330.x

[2] Chen TH-H, Yen M-F, Tung T-H. A computer simulation model for cost–effectiveness analysis of mass screening for Type 2 diabetes mellitus. Diabetes Res Clin Pract 2001;54:37–42. doi:10.1016/S0168-8227(01)00307-2

[3] Gillies CL, Lambert PC, Abrams KR, et al. Different strategies for screening & prevention of type 2 diabetes in adults: cost effectiveness analysis. BMJ 2008;336.http://www.bmj.com/content/336/7654/1180.long

[4] Herman WH, Hoerger TJ, Brandle M, et al. The cost-effectiveness of lifestyle modification or metformin in preventing type 2 diabetes in adults with impaired glucose tolerance. Ann Intern Med 2005;142:323–32.http://www.ncbi.nlm.nih.gov/pubmed/15738451

[5] Ikeda S, Kobayashi M, Tajima N. Cost-effectiveness analysis of voglibose for prevention of type 2 diabetes mellitus in Japanese patients with impaired glucose tolerance. 2010;1:252–8. doi:10.1111/j.2040-1124.2010.00052.x

[6] Johansson P, Östenson C-G, Hilding AM, et al. A cost-effectiveness analysis of a community-based diabetes prevention program in Sweden. Int J Technol Assess Health Care 2009;25:350–8. doi:10.1017/S0266462309990079

[7] Liu X, Li C, Gong H, et al. An economic evaluation for prevention of diabetes mellitus in a developing country: a modelling study. BMC Public Health 2013;13:729. doi:10.1186/1471-2458-13-729.

[8] Neumann A, Schwarz P, Lindholm L. Estimating the cost-effectiveness of lifestyle intervention programmes to prevent diabetes based on an example from Germany: Markov modelling. Cost Eff Resour Alloc 2011;9:17. doi:10.1186/1478-7547-9-17

[9] Neumann A, Lindholm L, Norberg M, et al. The cost-effectiveness of interventions targeting lifestyle change for the prevention of diabetes in a Swedish primary care & community based prevention program. Eur J Heal Econ 2017;18:905–19. doi:10.1007/s10198-016-0851-9

[10] Palmer AJ, Tucker DMD, Polkinghorne KR, et al. Cost & clinical implications of diabetes prevention in an Australian setting: A long-term modeling analysis. Prim Care Diabetes 2012;6:109–21. doi:10.1016/j.pcd.2011.10.006

[11] Palmer AJ, Roze S, Valentine WJ, et al. Intensive lifestyle changes or metformin in patients with impaired glucose tolerance: Modeling the long-term health economic implications of the diabetes prevention program in Australia, France, Germany, Switzerland, & the United Kingdom. Clin Ther 2004;26:304–21. doi:10.1016/S0149-2918(04)90029-X

[12] Roberts S, Craig D, Adler A, et al. Economic evaluation of type 2 diabetes prevention programmes: Markov model of low- & high-intensity lifestyle programmes & metformin in participants with different categories of intermediate hyperglycaemia. BMC Med 2018;16:16. doi:10.1186/s12916-017-0984-4

[13] Schaufler TM, Wolff M. Cost Effectiveness of Preventive Screening Programmes for Type 2 Diabetes Mellitus in Germany. Appl Health Econ Health Policy 2010;8:191–202. doi:10.2165/11532880-000000000-00000

[14] Smith KJ, Hsu HE, Roberts MS, et al. Cost-effectiveness analysis of efforts to reduce risk of type 2 diabetes & cardiovascular disease in southwestern Pennsylvania, 2005-2007. Prev Chronic Dis 2010;7:A109.http://www.ncbi.nlm.nih.gov/pubmed/20712936

[15] Wong CKH, Jiao F-F, Siu S-C, et al. Cost-Effectiveness of a Short Message Service Intervention to Prevent Type 2 Diabetes from Impaired Glucose Tolerance. J Diabetes Res 2016;2016:1–8. doi:10.1155/2016/1219581

[16] Zhou H, Isaman DJM, Messinger S, et al. A computer simulation model of diabetes progression, quality of life, & cost. Diabetes Care 2005;28:2856–63.http://www.ncbi.nlm.nih.gov/pubmed/16306545

[17] Zhuo X, Zhang P, Selvin E, et al. Alternative HbA1c Cutoffs to Identify High-Risk Adults for Diabetes Prevention. Am J Prev Med 2012;42:374–81. doi:10.1016/j.amepre.2012.01.003
