# Supplementary material for: How valid are projections of the future prevalence of diabetes? Rapid reviews of prevalence-based and Markov chain models and comparisons of different models’ projections for England
Source: BMJ Open. 2020 Mar 3;10(3):e033483. doi: 10.1136/bmjopen-2019-033483 (PMC7059487; doi:10.1136/bmjopen-2019-033483)
Supplement: Author's manuscript [file bmjopen-2019-033483.draft_revisions.pdf]

BMJ Open is committed to open peer review. As part of this commitment we make the peer review history of every article we publish publicly available.

When an article is published we post the peer reviewers' comments and the authors' responses online. We also post the versions of the paper that were used during peer review. These are the versions that the peer review comments apply to.

The versions of the paper that follow are the versions that were submitted during the peer review process. They are not the versions of record or the final published versions. They should not be cited or distributed as the published version of this manuscript.

BMJ Open is an open access journal and the full, final, typeset and author-corrected version of record of the manuscript is available on our site with no access controls, subscription charges or pay-per-view fees (http://bmjopen.bmj.com).

If you have any questions on BMJ Open's open peer review process please email info.bmjopen@bmj.com

BMJ Open

How valid are models' projections of the future prevalence of diabetes? Rapid reviews of epidemiological and Markov chain models and comparisons of different models' projections for England

Journal:	*BMJ Open*
Manuscript ID	bmjopen-2019-033483
Article Type:	Research
Date Submitted by the Author:	13-Aug-2019
Complete List of Authors:	Bevan, Gwyn; London School of Economics and Political Science, Management<br>De Poli, Chiara; London School of Economics and Political Science, Management<br>Keng, Mi; University of Oxford, Nuffield Department of Population Health<br>Raine, Rosalind; University College London, Department of Applied Health Research
Keywords:	Diabetes Mellitus, Prevalence, Markov Chains, Obesity, EPIDEMIOLOGY

SCHOLARONE™
Manuscripts

I, the Submitting Author has the right to grant and does grant on behalf of all authors of the Work (as defined in the below author licence), an exclusive licence and/or a non-exclusive licence for contributions from authors who are: i) UK Crown employees; ii) where BMJ has agreed a CC-BY licence shall apply, and/or iii) in accordance with the terms applicable for US Federal Government officers or employees acting as part of their official duties; on a worldwide, perpetual, irrevocable, royalty-free basis to BMJ Publishing Group Ltd ("BMJ") its licensees and where the relevant Journal is co-owned by BMJ to the co-owners of the Journal, to publish the Work in this journal and any other BMJ products and to exploit all rights, as set out in our licence.

The Submitting Author accepts and understands that any supply made under these terms is made by BMJ to the Submitting Author unless you are acting as an employee on behalf of your employer or a postgraduate student of an affiliated institution which is paying any applicable article publishing charge ("APC") for Open Access articles. Where the Submitting Author wishes to make the Work available on an Open Access basis (and intends to pay the relevant APC), the terms of reuse of such Open Access shall be governed by a Creative Commons licence – details of these licences and which Creative Commons licence will apply to this Work are set out in our licence referred to above.

Other than as permitted in any relevant BMJ Author's Self Archiving Policies, I confirm this Work has not been accepted for publication elsewhere, is not being considered for publication elsewhere and does not duplicate material already published. I confirm all authors consent to publication of this Work and authorise the granting of this licence.

For peer review only

How valid are models' projections of the future prevalence of diabetes? Rapid reviews of epidemiological and Markov chain models and comparisons of different models' projections for England

Gwyn Bevan*

emeritus professor of policy analysis

Department of Management, London School of Economics and Political Science, WC2A 2AE, London, UK.

Email: R.G.Bevan@lse.ac.uk

Mob: +44 (0)77867 88967

Chiara De Poli

Research fellow

Department of Management, London School of Economics and Political Science, WC2A 2AE, London, UK.

Mi Jun Keng (0000-0001-5979-1706)

researcher

Nuffield Department of Population Health, University of Oxford, OX3 7LF, Oxford, UK.

Rosalind Raine [3]

professor of applied health research

Department of Applied Health Research, UCL, WC1E 7HB, London, UK

* Corresponding author

For peer review only - http://bmjopen.bmj.com/site/about/guidelines.xhtml

How valid are projections of the future prevalence of diabetes? Rapid reviews of epidemiological and Markov chain models using England as a case study

Abstract

Objectives

To examine validity of epidemiological models giving projections of prevalence of diabetes in adults, in England and the UK, and of Markov chain models giving estimates of impacts of interventions to prevent type 2 diabetes (T2D).

Methods

Rapid reviews of epidemiological and Markov chain models. Estimation of the future prevalence of T2D in England: by Markov chain models; and from the trend in the prevalence of diabetes as recorded in the Quality and Outcomes Framework (QOF) estimated by Ordinary Least Squares (OLS) regression analysis.

Setting

Adult population in England and UK.

Main outcome measure

Prevalence of T2D in 2025.

Results

The epidemiological models reviewed use sample estimates of past prevalence rates by age and sex and projected population changes. Three most recent

For peer review only - http://bmjopen.bmj.com/site/about/guidelines.xhtml

models, including that of Public Health England (PHE), neither take account of increases in obesity, nor report confidence intervals.

The Markov chain models reviewed use transition probabilities between states of risk and death, estimated from various sources, to give projected impacts of the preventive interventions on the numbers of adults who go on to develop T2D. None of their accounts give the full matrix of transition probabilities, nor report tests of validation of their models' estimates of the impacts of preventive interventions on prevalence of T2D at the population level.

Projections of the prevalence of T2D in England in 2025 were (in millions, with 95% confidence intervals where available) by PHE, 3.95; from the QOF trend, 4.91 (4.79 to 5.03); and by our two Markov chain models, 5.64 and 9.10.

Conclusions

Governments require realistic projections of the future prevalence of T2D from epidemiological models that take account of increases in obesity; and estimates of the likely relative impacts of preventive interventions from models that have been validated against projections from realistic epidemiological models.

Article summary

Strengths and limitations of this study

- We undertook rapid reviews of epidemiological models and Markov chain models, which have been used to give projections of the future prevalence of diabetes to examine their data sources and assumptions.
- We compared projections of the future prevalence of diabetes in England from: reports for the epidemiological models; our own Markov chain models (which used transition probabilities from our review); and the trend in the prevalence of diagnosed diabetes as reported by general practitioners in England (estimated by ordinary least squares regression analysis).

For peer review only - http://bmjopen.bmj.com/site/about/guidelines.xhtml
For peer review only

- This study's limitations are that our reviews were rapid and our models are transparent and simple.

Keywords

Diabetes Mellitus

Prevalence

Forecasting

Markov Chains

Obesity

3,372 words

For peer review only

How valid are projections of the future prevalence of diabetes? Rapid reviews of epidemiological and Markov chain models using England as a case study

Introduction

Rigorous analysis of worldwide trends of increases in the preventable onset of Type 2 Diabetes (T2D) in adults justifies a call for the urgent of implementation of 'population-based interventions that prevent diabetes, enhance its early detection, and use lifestyle and pharmacological interventions to prevent or delay its progression to complications'.[1] In March 2015, NHS England and Public Health England (PHE) launched the National NHS Diabetes Prevention Programme (NDPP), which is a pragmatic lifestyle intervention that targets adults with intermediate hyperglycaemia (glucose levels associated with a high risk of developing T2D). The NDPP aims 'to significantly reduce the 4 million people in England otherwise expected to have Type 2 Diabetes (T2D) by 2025' based on evidence from 'well-designed randomised controlled trials (RCTs) in Finland, the USA, Japan, China and India'.[2] Many studies have used Markov chain models to estimate the impacts of such preventive interventions using transition probabilities between states: 'normoglycaemia' (glucose levels associated with a low risk of developing T2D), and 'intermediate hyperglycaemia' T2D and death. When we used these models,[3] we found, however, that our projections of the future prevalence of T2D in 2025 in England, in the absence of a preventive intervention, was much higher than 4 million, which is based on PHE's epidemiological model. Epidemiological models give future projections of the prevalence of T2D (at future time t, N(t)) by multiplying projections of the country's population by age and sex (at time t (**P**(t)) by projections of age-specific prevalence of diabetes (at time t, **D**(t)). (N(t) = **D**(t)* **P**(t)).) Hence this study, which is a critical review of methods of epidemiological and Markov chain models. Although we have used England for the purpose of comparing projections by these different models, our study raises

For peer review only - http://bmjopen.bmj.com/site/about/guidelines.xhtml

general questions about their validity. And hence of the evidence available to governments assessing the urgency of preventing T2D and choosing between different interventions. We consider only adults with diabetes. We use 'diabetes' to cover all types of diabetes, T2D for adults with type 2, 'true' prevalence for both diagnosed and undiagnosed diabetes and T2D.

Methods

Rapid reviews

In March 2018, we undertook two rapid reviews of articles published at any available on Web of science and PubMed, which together provide a comprehensive coverage of the literature in the medical and applied health research fields. Review 1 aimed to identify primary studies published from 2010 of epidemiological models giving estimates of the prevalence of diabetes in adults in England or the UK. We examined how the models take account of future changes in age-specific prevalence rates and test their validity.

Review 2 aimed to identify primary studies using Markov chain models that reported results of interventions to prevent T2D. We included articles using Markov chain models to run economic analyses, utility analyses and cost effectiveness analyses of interventions targeting people diagnosed with T2D, or with intermediate hyperglycaemia according to different measures: Glycated Haemoglobin (HbA1c), Impaired Fasting Glucose (IFG), and Impaired Glucose Tolerance (IGT) (Definitions are given in Table 1.1 of Appendix 1). We compared models' transition probabilities, estimates of the future prevalence of T2D without a preventive intervention, and tests of validation.

Articles included in each review were critically appraised and technical specifications of the models and projections were extracted and tabulated. The flowcharts in Figures 1 and 2 show the screening process. Appendix 2 gives the search strategy for each review and more details on the review of Markov

For peer review only - http://bmjopen.bmj.com/site/about/guidelines.xhtml

models. We deemed these rapid reviews to be sufficient in identifying the principal methods of each type of models.

Figure 1 - Review flowcharts of epidemiological and Markov chain models to go about here

Figure 2: Review flowcharts of epidemiological and Markov chain models to go about here

Markov chain model

From review 2 we derived matrices of transition probabilities to develop our own Markov chain models (see Figure 3), which are based on a cycle length of 1 year, to make projections of T2D cases in England without an intervention, up to 2035. The data sources of our estimates for England, of the prevalence of diabetes, intermediate hyperglycaemia and normoglycaemia; and of mortality rates of those with T2D, intermediate hyperglycaemia and normoglycaemia are given in Table 1.2 of Appendix 1.

Given doubts over the reliability of diagnosing intermediate hyperglycaemia (IH),[4] we examined the robustness of our results by using the PHE estimate (IH = 5.05 million), and the extreme value of zero (IH = 0). The hazard ratios for those with intermediate hyperglycaemia found in a systematic review[5] defined by HbA1c and IGT were 0.97 and 1.32. We used 1.32 for IGT, but 1 for HbA1c because their estimate of 0,97 is not significantly different from 1.

Figure 3: Our Markov chain model to go about here

Estimating the trend in diagnosed diabetes

We estimated, by OLS regression analysis (using R),[6] the trend increase in numbers diagnosed with diabetes by general practitioners in England, as reported in the Quality Outcomes Framework (QOF) from 2004-05 (2004) to 2017-18 (2017)).[7] We used these estimates to give projections of the future prevalence of diagnosed diabetes to 2035.

For peer review only - http://bmjopen.bmj.com/site/about/guidelines.xhtml

Comparing projections of the prevalence of diabetes

We compared three sets of projections of the prevalence of diabetes and T2D in England from:

- different epidemiological models,
- the trend in QOF data,
- our Markov chain models.

The ratios we used for making comparisons across different estimates and the sources are given in Table 1.3 of Appendix 1.

Patients and public involvement

Patients and the public were not involved in this research study.

Results

Rapid review 1: Methods of epidemiological models

Rapid review 1 of methods of epidemiological models retrieved 633 articles. A further five were snowballed. After removing duplicates, we screened 597 articles, of which 11 were relevant and fully assessed. After reviewing the full articles, five were excluded and seven were included in our analysis[8–14]. This review identified four different underlying models described in Table 1 which have been used to give five different projections of the future prevalence of diabetes for England and the UK. Two models produce global estimates: Shaw et al,[8] Guariguata et al[15], which is used by Whiting et al[9] and Guariguata et al;[10] and two for England only, the PHE model,[12] and the Association of Public Health Observatories (APHO) Diabetes Prevalence Model,[13] which is used by Hex et al[11] and Gatineau et al.[14]

For peer review only - http://bmjopen.bmj.com/site/about/guidelines.xhtml

Table 1: Methods of epidemiological models

Model	Method of estimation	Prevalence rates used for projections	Validation against QOF data?	Model validation?	Confidence intervals?
Shaw et al[8]	Logistic regression	Age & sex	No	No	No
Guariguata et al[15]	Logistic regression	Age & sex, & urban / rural	No	No	No
Association of Public Health Observatories (APHO)[13,14]	Direct estimation from HSE for age, sex, & IMD. Trend in obesity estimated by linear regression.	Age & sex, Index of Multiple Deprivation (2004), Ethnicity & increases in obesity	Yes for 2008/09	No	Yes
PHE[12]	Logistic regression	Age & sex, ethnicity, IMD 2015	Yes for 2014/15	Yes: refitting model on 70% of data & assessing against remaining 30%	No

Each epidemiological model uses: projected population changes; and estimates of the true age-specific prevalence rates of diabetes, from past annual Health Surveys for England (HSE), which are subject to two limitations. First, the small size of the sample means that the point estimate for the year of the survey is surrounded by large confidence interval estimates. Gatineau et al indicate that the HSE survey for 2013 gives point estimate of prevalence of 7.3% with confidence interval estimates ranging from 4.3 to 10.3%.[14] The PHE model[12] reduces the sampling error from HSE by using three years of data (2012, 2013 and 2014). Second, the HSE estimates of prevalence are based on those who self-reported a diabetes diagnosis made by a doctor (by HbA1c or FPG); and, for those who have not been diagnosed and agreed to have a blood test, having a HbA1c value of 6.5% or more.[12] Hence these estimates may be in error from because of poor reliability of self-reporting or because of actual diagnostic errors. Barry et al (p. 9) report that 'The most commonly used test (HbA1c) is neither sensitive nor specific; the fasting glucose test is specific but not sensitive, and the fasting glucose test is neither sensitive nor specific'. [4] Holman et al (p.6) pointed out, however, that 'Although HbA1c and fasting identify different groups of people with undiagnosed diabetes, the proportion of people that are identified is similar'.[13]

For peer review only - http://bmjopen.bmj.com/site/about/guidelines.xhtml

Our review aimed to answer two questions.

1. *Tests of validation?* A basic test of the validity of a forecasting model is to apply this to past data to predict a known future: e.g. does the model using HSE data from 2004 predict prevalence as estimated from HSE data in 2014? None of the accounts of the models we reviewed reports such a test. The PHE model[12] was validated by refitting the model on 70% of the data (randomly selected) and checking its estimates against the remaining 30% of data.
2. *Modelling future changes in age-specific prevalence rates?* Only the APHO model[13] aimed to do this by estimating the net effect of trends in: changes in ethnicity; and being overweight and obese to create a sex-specific obesity adjustment index. They did not, however, give details of how that index was modelled. The other three models[8,12,15] assumed that future age-specific prevalence of diabetes would be as estimated from past HSEs.

The epidemiological models we reviewed are focused on estimating geographical variations in the future prevalence of diabetes within countries, rather than giving sound estimates of future totals.

Rapid review 2: Markov chain models

Rapid review 2 of Markov chain models identified 304 articles. An additional one was snowballed. After removing duplicates, 222 articles were screened, 20 of them were considered relevant and fully assessed. Of these, one was excluded because we could not locate it, one did not report the results of a Markov chain model, and one modelled the progression from diabetes to its complications only. Table 2 gives details of the remaining 17 articles,[16–32]ordered in terms of their completeness of information on transition probabilities. (More details are given in Appendix 2). Two articles did not report the measure of intermediate hyperglycaemia used.[28,32] Twelve reported a model using one risk measure only: nine models used IGT,[17,18,20–23,26,27,31] two HbA1c[25,29] and one FPG only.[16] Neumann et al reported two models, using

For peer review only - http://bmjopen.bmj.com/site/about/guidelines.xhtml

IFG and IGT;[17] and Roberts et al[24], two models using HbA1c, IGT and IFG. Hence, we reviewed 20 models.

For peer review only - http://bmjopen.bmj.com/site/about/guidelines.xhtml

Table 2: Transition probabilities reported in different models

Reference	Measure of intermediate hyperglycaemia	Country	Normoglycaemia to Intermediate hyperglycaemia	Intermediate hyperglycaemia to Normoglycaemia	Normoglycaemia to T2D	T2D to Normoglycaemia	Intermediate hyperglycaemia to T2D	T2D to Intermediate hyperglycaemia	Mortality rates
Johansson et al, 2009 **[16]	FPG	Sweden	**NR**	**NR**	**NR**	**NR**	**NR**	**NR**	**NR**
Herman et al, 2005[27]	IGT	USA	**NR**	**NR**	**NR**	**NR**	10.80%[33]	**NR**	**NR**
Palmer et at, 2004[26]	IGT	Australia, France, Germany, Switzerland, & UK	**NR**	**NR**	**NR**	**NR**	11%[34]	**NR**	Intermediate hyperglycaemia:1.37 (1.05 - 1.79) Undiagnosed T2D: 1.76 (1.17 – 2.66) Diagnosed T2D: 2.26 (1.78 – 2.87)
Zhuo et al, 2012[29]	HbA1c	USA	**NR**	**NR**	**NR**	**NR**	0.07% to 18.9%[35]	**NR**	**NR**
Chen et al, 2001[28]	**NR**	Taiwan	**NR**	**NR**	1.10%[36]	**NR**	**NR**	**NR**	**NR**
Zhou et al 2005[25]	HbA1c	USA	**NR**	**NR**	0%	0%	**NR**	0%	**NR**
Schaufler et al, 2010[30]	IGT or IFG	Germany	male, 2.23% female, 1.45%[37]	**NR**	male, 2.51% & female, 1.66%[37]	**NR**	male, 4.79% female, 4.23%[37]	**NR**	**NR**[38] **(Source given for higher mortality rates for T2D)**
Gillies et al, 2008[18]	IGT	UK	< 65, 1.66% > 65, 2.49%[39]	**NR**	**NR**	**NR**	1.96% based on 12 studies[34,39–49]	**NR**	Increased risk of death with diabetes (hazard ratio) 0.756 (SE = 0.087) [50] 1% increase in HbA1c (hazard ratio) 0.104 (SE =0.039 [51]
Ikeda et al, 2010[31]	IGT	Japan	3.10% [52]	**NR**	0%	0%	**NR**	0%	Intermediate hyperglycaemia: 1.35 T2DM: 3.03 [53]
Smith et al, 2010[32]	NR	USA	4% [54]	**NR**	0.40% [55]	0%	10.80%[27]	0%	Intermediate hyperglycaemia: 1.7 [56]

For peer review only

Reference	Measure of intermediate hyperglycaemia	Country	Normoglycaemia to Intermediate hyperglycaemia	Intermediate hyperglycaemia to Normoglycaemia	Normoglycaemia to T2D	T2D to Normoglycaemia	Intermediate hyperglycaemia to T2D	T2D to Intermediate hyperglycaemia	Mortality rates
									stable T2D: 2 [57] complicated T2D: 2.4 [58]
Neumann et al, 2017[17]	IGT	Sweden	Risk equation reported	Risk equation reported	0%	0%	Risk equation reported	Risk equation reported	No increased risk for intermediate hyperglycaemia. T2D mortality not reported.
Caro et al, 2004[20]	IGT	Canada	16.30% (original estimate)	16.20% (original estimate)	0%	0%	6.30% (original estimate)	0%	Intermediate hyperglycaemia: 1.45 (original estimate)
Neumann et al, 2011[21]	IGT	Germany	16.30% [20]	16.20% [20]	0%	0%	6.00%[59]	0.50% (original estimate)	**NR**
Liu et al, 2013[22]	IGT	China	1.28% [60]	11.60% [61]	0%	0%	initiation ages<br>• 25: 0.644%<br>• 40: 16.7%<br>• 60: 57.8%<br>[62–64]	0%	**NR**
Wong et al, 2016[23]	IGT	Hong Kong	16.30% [20]	16.20% [20]	0%	0%	years 1-3 11% [34]<br>years > 4 5.6% [65]	0%	Intermediate hyperglycaemia: 1.50 (1.10–2.00) T2DM: 2.30 (1.60–3.20) [19]
Roberts et al, 2018[24]	IGT	England	6.33% [39]	8.97% [66]	0%	0%	4.55%[67]	0%	Intermediate hyperglycaemia: 1.50 T2D: 1.9 [68]
	HbA1c	England	6.86% [39]	8.97% [66]	0%	0%	3.55%[67]	0%	Intermediate hyperglycaemia: 1.2 T2D: 1.6 [68]
	IFG (ADA)	England	6.86% [39]	8.97% [66]	0%	0%	4.74%[67]	0%	Intermediate hyperglycaemia: 1.2 T2D: 1.6 [68]
Range	IGT		1.28 -16.30%	8.97-16.20%	0.00-4.6%	0%	1.96-11.00%	0.00-0.5%	Intermediate hyperglycaemia:1.35-1.7 T2D: 1.76-3.03

For peer review only - http://bmjopen.bmj.com/site/about/guidelines.xhtml

Reference	Measure of intermediate hyperglycaemia	Country	Normoglycaemia to Intermediate hyperglycaemia	Intermediate hyperglycaemia to Normoglycaemia	Normoglycaemia to T2D	T2D to Normoglycaemia	Intermediate hyperglycaemia to T2D	T2D to Intermediate hyperglycaemia	Mortality rates
Meta-analyses	IGT						4.55%[67]		Intermediate hyperglycaemia: 1.32 (1.23 to 1.40) [5]
	HbA1c						3.55%[67]		Intermediate hyperglycaemia: 0.97 (0.88 to 1.07) [5]
	IFG (ADA)						3.54%[67]		Intermediate hyperglycaemia: 1.13, (1.02 - 1.25) [5]

Key and Notes:

NR: not reported, 0%: not allowed

*Relative risk over normoglycaemia. Ranges in parentheses are 95% confidence intervals

** The model and data sources were described in a technical report

For peer review only - http://bmjopen.bmj.com/site/about/guidelines.xhtml

These Markov chain models use two different sources of data to estimate transition probabilities: between states other than death (ideally from RCTs or meta analyses); and from these states to death (based on mortality rates of a country. As these models require transition probabilities from each state to sum to one, the validity of the interaction between two sets of transition probabilities needs to be tested. We have done this by comparing our models' estimates of the number of T2D cases in the absence of any preventive intervention with those from epidemiological models.

Our review aimed to answer two questions:

1. *How do transition probabilities compare?* Table 2 shows that of the 17 articles only five reported the full set of transition probabilities between states other than death (i.e. normoglycaemia, intermediate hyperglycaemia, and T2D). All models, except that of Neumann et al,[21] allow transitions from T2D to death only. Neumann et al[21] allow movement (at a low rate, 0.5%) from T2D to intermediate hyperglycaemia (IGT) (because 'this transition exists but seldom occurs', p 4). Only two models allow transition from normoglycaemia directly to T2D: Schaufler et al[30] (IFG or IGT - for males, 2.51% and females, 1.66%) and Smith et al (measure of intermediate hyperglycaemia not specified, 0.40%).[32] Table 2 shows that wide ranges of transition probabilities used by the different IGT models: from normoglycaemia to intermediate hyperglycaemia, 1.28 to 16.30%; from intermediate hyperglycaemia to low, 8.97-16.20%; normoglycaemia to T2D, 0.00-4.6%; intermediate hyperglycaemia to T2D, 1.96-11.00%. A meta-analysis recommended a rate of 4.55% for the last.[67]

   No article reports the transition probabilities from different states to death (i.e. mortality rates for each state). Six articles report the relative risk of mortality for intermediate hyperglycaemia and T2D compared with normoglycaemia. For IGT these ranged for intermediate hyperglycaemia (IGT) from 1.35 to 1.7, and for T2D from 1.76 to 3.03. Roberts et al[24] report this for HbA1c to be 1.2. A systematic review and

For peer review only - http://bmjopen.bmj.com/site/about/guidelines.xhtml

meta-analysis[5] derived estimates (with 95% confidence intervals) to be: for IGT 1.32 (1.23 to 1.40) and for HbA1c 0.97 (0.88 to 1.07). One article[21] reported a matrix in which probabilities of transitions between states other than death sum to one, which implies no one dies.

2. *How were models validated?* Of the 17 articles, estimating the impacts of preventive interventions on prevalence of T2D, only four[14,20,29,69] modelled the general population (with normoglycaemia and intermediate hyperglycaemia); and, of these, only Caro et al [20] reported estimates of those developing T2D in the absence of a preventive intervention: 9.6% of 55-year-old men and women over three years. They did not report a check of their estimate against other projections. Of the other articles, which modelled populations with intermediate hyperglycaemia only, only three reported estimates of the percentages developing T2D in the absence of intervention [11,19,20]. Only two reported tests of validation: against the observed incidence in RCTs (correlation coefficient of 0.9987), and National Diabetes Audit 2015-2016. [70] They estimated these percentages developing T2D over 10 years to be: for those with IGT over 50%[20] and 23%;[11] and, for both IFG and HbA1c19%.[11]

The primary focus of the articles we reviewed is on estimating the ratio of costs to benefits of preventive interventions for those with IGT. None reported another ratio that governments need to know: of the numbers of T2D cases prevented to projections of its future prevalence in the general population.

Our Markov chain models

Our Markov chain models are designed to use available data for England with one transition probability only between states. As PHE identify those with intermediate hyperglycaemia using HbA1c (from 5.7% to 6.4%),[12] the model used by Roberts et al[24] for HbA1c is most appropriate for projecting the prevalence of T2D in England. They used the recommended transition probabilities from different risk measures of intermediate hyperglycaemia to T2D identified by a meta-analysis.[67] Neumann et al[21] and Caro et al[20] have similar transition probabilities with higher rates of transition than Roberts

For peer review only - http://bmjopen.bmj.com/site/about/guidelines.xhtml

et al[24] for IGT from normoglycaemia to intermediate hyperglycaemia, and intermediate hyperglycaemia to T2D: 16.3% and 6.00% compared with 6.33% and 4.55%. We used the transition probabilities used by Neumann et al[21] because that is more recent. Model 1 is based on Roberts et al (HbA1c).[24] Model 2 is Model 1 modified to give the projections of PHE. Model 2's transition probability from intermediate hyperglycaemia to T2D (0.013) is a third of that of Model 1 (0.036) and below the lowest rate of any model we reviewed (0.02). (Model 2 has a corresponding increase in the transition probability of remaining in intermediate hyperglycaemia (0.836 to 0.878).) Model 3 is based on Neumann et al.[21] Details of the models are given in Table 1.4 of Appendix 1.

Estimating the trend in diagnosed diabetes

Table 3 reports the OLS estimate of the trend in diagnosed diabetes from QOF data, which gives an annual rate of increase of 11%.

Table 3: The trend model from QOF data

Coefficients	Value	Standard error	T	Pr > |t|	Lower bound (95%)	Upper bound (95%)
Intercept	-229	2.436	94.228	< 0.0001	-234.889	-224.167
Year	0.115	0.001	95.235	< 0.0001	0.113	0.118
Adjusted R-squared	0.9987					

Comparing projections of the future prevalence of T2D

Table 4 gives: for the different epidemiological models their defined populations, data sources, and projections of diabetes true prevalence (in millions); and comparable estimates of the true prevalence of diabetes from the QOF trend (increased by a third). It also gives the annual rate of increase in prevalence from the first in the series to the last. Table 4 shows that, for the three models that do not allow for increase in prevalence rates by age and sex,[8–10] the older the

For peer review only - http://bmjopen.bmj.com/site/about/guidelines.xhtml

HSE data used, the lower is the estimate of the rate of increase in prevalence for England.

For peer review only

For peer review only - http://bmjopen.bmj.com/site/about/guidelines.xhtml

Table 4: True diabetes prevalence (millions) estimated by different epidemiological models and from the QOF Trend

Source of estimate	Population	Data source	Details of series									
			First year	Prevalence	Final Year	Prevalence	Annual rate of increase (%)*	Projections				
								2015	2020	2025	2030	2035
Shaw et al[8]	UK: 20 to 79 (UN, 2007)	HSE (2003)	2010	2.14	2030	2.55	2.0				2.55	
Whiting et al[9]	UK: 20 to 79 (UN, 2011)	HSE (2004 & 2009)	2011	3.06	2030	3.65	3.1				3.65	
Guariguata et al[10]	UK: 20 to 79 (UN, 2011)	HSE (2004)	2013	2.98	2035	3.62	2.9					3.62
Holman et al[13]	England: >15 (ONS)	HSE (2006)	2010	3.10	2030	4.60 (3.25-6.88)	7.5	3.47 (2.47-5.07)	3.82 (2.70-5.62)	4.19 (2.93-6.19)	4.60 (3.25-6.88)	
PHE[12]	England: >15 (ONS)	HSE (2012, 2013 & 2014)	2015	3.81	2035	4.94	5.6	3.81	4.09	4.39	4.68	4.94
QOF trend**	England: >15 registered with GPs	QOF (2004-05 to 2017-18)	2004-05	1.77	2017	3.20	11.0	3.99 (3.88-4.09)	4.72 (4.61-4.84)	5.46 (5.32-5.59)	6.19 (6.04-6.35)	6.93 (6.75-7.11)

Notes:

*Estimated as the rate of increase from the first estimate to the last

** To estimate the true prevalence from the QOF trend these estimates were increased by a third.

For peer review only

Global models are used to give three projections (in millions) for diabetes prevalence in the UK (aged 20 to 79): for 2030 (2.55[8] and 3.65[9]) and 2035 (3.62).[10] Each projection is below the PHE[71] model's estimate for England for 2015 (3.81) (based on HSEs for 2012, 2103 and 2014). There are two reasons for this: their low rates of increase over time; and excision of those over 79, who we estimated to account for nearly 30% who would be over 15 in England and develop diabetes (see Table 1.3 of Appendix 1). The projections by these global models are not examined further.

Two models give projections for England (aged over 15): the PHE model[71] gives projections for 2030 (4.68) and 2035 (4.94); and APHO only up to 2030 (4.60) (with 95% confidence intervals from 3.25 to 6.88).[13] Although the two accounts of the APHO model report the same projection for 2030; one estimated the prevalence of diabetes in 2010 (3.10)[13] to be higher than the other for 2013 (2.17).[14] Also, one attributed approximately half of the increase in prevalence to 2030 to increases in obesity,[13] the other estimated this to have been a third.[14]

Figure 4 compares the projections of the true prevalence of diabetes: by PHE, and (with 95% confidence intervals) by Holman et al[13], and from the QOF trend (for the last two we show their 95% confidence interval estimates). The estimates from the QOF trend are the highest and towards the upper end of the 95% confidence intervals of Holman et al.[13] For 2025, projections (with 95% confidence interval estimates where available) are as follows: by Holman et al, 4.19 (2.93-6.19); by PHE,[71] 4.39; from the QOF trend, 5.46 (5.32-5.59).

Figure 4: Projections of true diabetes prevalence by PHE, Holman et al & from the QOF trend (millions): 2005 to 2035 to go about here

Figure 5 compares projections of the true prevalence of T2D in England to 2035 from PHE, the QOF trend, and the Markov models. Table 5 gives projections in

For peer review only - http://bmjopen.bmj.com/site/about/guidelines.xhtml

millions for 2025. These show that the projections: by Model 2 replicated the projections by PHE; by Model 1 are above those from the QOF trend; by Model 3 seem to be implausibly explosive. Figure 6 and Table 5 also show that projections by models 1 and 3 are robust to errors in the estimate of the numbers of those with intermediate hyperglycaemia in 2015.

Table 5: Projections of adults with T2D in England for 2025

Model	Projections for 2025 (millions)			
	Statistical		Markov (numbers with intermediate hyperglycaemia in 2015)	
	Point estimate	95% confidence intervals	5.05*	Zero
PHE	3.95	n.a.		
QOF trend;	4.91	4.79 to 5.03		
Model 1**			5.64	5.05
Model 2 ***			3.86	
Model 3****			9.10	8.60

Notes

n.a Not available

* as estimated by PHE

**based on Roberts et al,[24]

***based on Roberts et al,[24] but modified to reproduce the QOF trend to 2035

**** based on Neuman et al[21]

Figure 5: Projections of adults with T2D in England to go about here

Discussion

The four epidemiological models we reviewed[8,12,13,15] use past estimated prevalence rates by age and sex and projected changes in populations. They are focused on estimating geographical variations in the future prevalence of diabetes within countries, rather than giving sound estimates of future totals. Only one model aims to take account of increases in prevalence rates.[13] No model was validated by using past data to predict a known future.

Of the five projections of diabetes prevalence, for England and the UK we reviewed,[8–10,12,13] only one[13] reported confidence intervals. Three projections of diabetes prevalence for the UK (aged 20 to 79) by global models for 2030[8,9] and 2035[10] are below the PHE estimate for 2015 for England

For peer review only - http://bmjopen.bmj.com/site/about/guidelines.xhtml

(over 15). This raises questions over the validity of their global projections and their excision of those over 79 (estimated to account for nearly 30% of developing T2D after 2030). The estimates of T2D prevalence (in millions) in England for 2025 (with 95% confidence intervals where available) were: by PHE[71] 3.95; by the APHO model[13,14] 3.77 (2.64 to 5.57); from the QOF trend, 4.91 (4.79 to 5.03).

Markov chain models of the impacts of interventions that aim to prevent T2D require estimates of transition probabilities between states other than death and from these states to death, which are based on different sources. None of the articles we reviewed reported the complete matrix of transition probabilities. Only two[19,24] reported checks on the validity of their models using their projections of numbers developing T2D with no intervention, and none against projections from epidemiological models. This disconnect means that governments lack information on what the impact on the future prevalence of T2D might be if, like England they were to roll out at scale interventions like the NDPP. We found that projections from two of our own Markov chain models (based on those of Roberts et al (for HbA1c),[24] and Neuman et al (for IGT)[21] gave projections (in millions) with T2D for England (for 2025 of 5.64 and 9.1 million), which are above all estimates from the epidemiological models we reviewed. Our model that reproduced PHE's projections has a lower rate of transition from intermediate hyperglycaemia to T2D than any of the models we reviewed.

For peer review only

The limitations of our research are that we did not undertake systematic reviews, hence we may have omitted relevant articles. We also developed simple transparent Markov chain models and a simple regression model to project a trend using QOF data.

Conclusions

There are three implications of our study. First, methods of current epidemiological models are designed to underestimate the scale of increases in the future prevalence of T2D, and hence the urgency for governments of

implementing preventive interventions. Second, models used to assess the preventive interventions lack transparency and tests of validity. Third, we need research to remedy these deficiencies.

Author contributions

Mi Jun Keng did the original work in developing initial Markov Chain models to estimate the impacts of preventive interventions on the future prevalence of Type 2 Diabetes (T2D) in England and has been involved throughout this project. Chiara De Poli worked with Mi Jun in developing those models, organised workshops to generate estimates to be used by models, undertook the rapid reviews of epidemiological and Markov Chain models, and commented on drafts of this paper. Gwyn Bevan prepared drafts of the paper, reviewed epidemiological and Markov Chain models, developed the models used in this paper, and undertook comparisons of projections. Rosalind Raine took part in the workshops on the models, reviewed our methods and findings, and commented on drafts.

Data statement

The data we have used and the models we have developed are fully described in Appendices to this article.

For peer review only

For peer review only - http://bmjopen.bmj.com/site/about/guidelines.xhtml

Declaration of interests

There are no conflicts of interest.

Funding

Funding was from the National Institute for Health Research (NIHR) Collaboration for Leadership in Applied Health Research and Care North Thames at Barts Health NHS Trust, which had no role in the writing of the manuscript or the decision to submit it for publication. None of us has been paid to write this article. The corresponding author has had full access to all the data in the study and had final responsibility for the decision to submit for publication.

Ethics approval

No ethics approval was sought for this study.

References

1 NCD Risk Factor Collaboration (NCD-RisC). Worldwide trends in diabetes since 1980: a pooled analysis of 751 population-based studies with 4.4 million participants. *Lancet (London, England)* 2016;**387**:1513–30. doi:10.1016/S0140-6736(16)00618-8

2 Public Health England, NHS England. National NHS Diabetes initiative launched in major bid to prevent illness (press release). 2015.

3 De Poli C, Bevan G, Keng MJ, *et al.* Impact of interventions to prevent diabetes in England: a simulation model. *Lancet* 2017;**390**:S36. doi:10.1016/S0140-6736(17)32971-9

4 Barry E, Roberts S, Oke J, *et al.* Efficacy and effectiveness of screen and treat

For peer review only - http://bmjopen.bmj.com/site/about/guidelines.xhtml

policies in prevention of type 2 diabetes: systematic review and meta-analysis of screening tests and interventions. *BMJ* 2017;**356356**. doi:10.1136/bmj.i6538

5 Huang Y, Cai X, Mai W, *et al.* Association between prediabetes and risk of cardiovascular disease and all cause mortality: systematic review and meta-analysis. *BMJ* 2016;**355**:i5953. doi:10.1136/bmj.i5953

6 RStudio Team. RStudio: Integrated Development for R. 2015.

7 NHS Digital. Quality and Outcomes Framework (QOF) - 2016-17. 2017.

8 Shaw JEE, Sicree RAA, Zimmet PZZ. Global estimates of the prevalence of diabetes for 2010 and 2030. *Diabetes Res Clin Pract* 2010;**87**:4–14. doi:10.1016/j.diabres.2009.10.007

9 Whiting DR, Guariguata L, Weil C, *et al.* IDF diabetes atlas: global estimates of the prevalence of diabetes for 2011 and 2030. *Diabetes Res Clin Pract* 2011;**94**:311–21. doi:10.1016/j.diabres.2011.10.029

10 Guariguata L, Whiting DR, Hambleton I, *et al.* Global estimates of diabetes prevalence for 2013 and projections for 2035. *Diabetes Res Clin Pract* 2014;**103**:137–49. doi:10.1016/j.diabres.2013.11.002

11 Hex N, Bartlett C, Wright D, *et al.* Estimating the current and future costs of Type 1 and Type 2 diabetes in the UK, including direct health costs and indirect societal and productivity costs. *Diabet Med* 2012;**29**:855–62. doi:10.1111/j.1464-5491.2012.03698.x

12 Public Health England. Technical document for the diabetes prevalence model for England 2016. London: 2016.

13 Holman N, Forouhi NG, Goyder E, *et al.* The Association of Public Health Observatories (APHO) Diabetes Prevalence Model: estimates of total diabetes prevalence for England, 2010-2030. *Diabet Med* 2011;**28**:575–82. doi:10.1111/j.1464-5491.2010.03216.x

14 Gatineau M, Hancock C, Holman N, *et al.* Adult obesity and type 2 diabetes. London, UK: 2014.

15 Guariguata L, Whiting D, Weil C, *et al.* The International Diabetes Federation

For peer review only

diabetes atlas methodology for estimating global and national prevalence of diabetes in adults. *Diabetes Res Clin Pract* 2011;**94**:322–32. doi:10.1016/j.diabres.2011.10.040

16 Johansson P, Östenson C-G, Hilding AM, *et al.* A cost-effectiveness analysis of a community-based diabetes prevention program in Sweden. *Int J Technol Assess Health Care* 2009;**25**:350–8. doi:10.1017/S0266462309990079

17 Neumann A, Lindholm L, Norberg M, *et al.* The cost-effectiveness of interventions targeting lifestyle change for the prevention of diabetes in a Swedish primary care and community based prevention program. *Eur J Heal Econ* 2017;**18**:905–19. doi:10.1007/s10198-016-0851-9

18 Gillies CL, Lambert PC, Abrams KR, *et al.* Different strategies for screening and prevention of type 2 diabetes in adults: cost effectiveness analysis. *BMJ* 2008;**336**.

19 Palmer AJ, Tucker DMD, Polkinghorne KR, *et al.* Cost and clinical implications of diabetes prevention in an Australian setting: A long-term modeling analysis. *Prim Care Diabetes* 2012;**6**:109–21. doi:10.1016/j.pcd.2011.10.006

20 Caro JJ, Getsios D, Caro I, *et al.* Economic evaluation of therapeutic interventions to prevent Type 2 diabetes in Canada. *Diabet Med* 2004;**21**:1229–36. doi:10.1111/j.1464-5491.2004.01330.x

21 Neumann A, Schwarz P, Lindholm L. Estimating the cost-effectiveness of lifestyle intervention programmes to prevent diabetes based on an example from Germany: Markov modelling. *Cost Eff Resour Alloc* 2011;**9**:17. doi:10.1186/1478-7547-9-17

22 Liu X, Li C, Gong H, *et al.* An economic evaluation for prevention of diabetes mellitus in a developing country: a modelling study. *BMC Public Health* 2013;**13**:729. doi:10.1186/1471-2458-13-729

23 Wong CKH, Jiao F-F, Siu S-C, *et al.* Cost-Effectiveness of a Short Message Service Intervention to Prevent Type 2 Diabetes from Impaired Glucose Tolerance. *J Diabetes Res* 2016;**2016**:1–8. doi:10.1155/2016/1219581

24 Roberts S, Craig D, Adler A, *et al.* Economic evaluation of type 2 diabetes prevention programmes: Markov model of low- and high-intensity lifestyle

For peer review only - http://bmjopen.bmj.com/site/about/guidelines.xhtml

programmes and metformin in participants with different categories of intermediate hyperglycaemia. *BMC Med* 2018;**16**:16. doi:10.1186/s12916-017-0984-4

25 Zhou H, Isaman DJM, Messinger S, *et al.* A computer simulation model of diabetes progression, quality of life, and cost. *Diabetes Care* 2005;**28**:2856–63.

26 Palmer AJ, Roze S, Valentine WJ, *et al.* Intensive lifestyle changes or metformin in patients with impaired glucose tolerance: Modeling the long-term health economic implications of the diabetes prevention program in Australia, France, Germany, Switzerland, and the United Kingdom. *Clin Ther* 2004;**26**:304–21. doi:10.1016/S0149-2918(04)90029-X

27 Herman WH, Hoerger TJ, Brandle M, *et al.* The cost-effectiveness of lifestyle modification or metformin in preventing type 2 diabetes in adults with impaired glucose tolerance. *Ann Intern Med* 2005;**142**:323–32.

28 Chen TH-H, Yen M-F, Tung T-H. A computer simulation model for cost–effectiveness analysis of mass screening for Type 2 diabetes mellitus. *Diabetes Res Clin Pract* 2001;**54**:37–42. doi:10.1016/S0168-8227(01)00307-2

29 Zhuo X, Zhang P, Selvin E, *et al.* Alternative HbA1c Cutoffs to Identify High-Risk Adults for Diabetes Prevention. *Am J Prev Med* 2012;**42**:374–81. doi:10.1016/j.amepre.2012.01.003

30 Schaufler TM, Wolff M. Cost Effectiveness of Preventive Screening Programmes for Type 2 Diabetes Mellitus in Germany. *Appl Health Econ Health Policy* 2010;**8**:191–202. doi:10.2165/11532880-000000000-00000

31 Ikeda S, Kobayashi M, Tajima N. Cost-effectiveness analysis of voglibose for prevention of type 2 diabetes mellitus in Japanese patients with impaired glucose tolerance. 2010;**1**:252–8. doi:10.1111/j.2040-1124.2010.00052.x

32 Smith KJ, Hsu HE, Roberts MS, *et al.* Cost-effectiveness analysis of efforts to reduce risk of type 2 diabetes and cardiovascular disease in southwestern Pennsylvania, 2005-2007. *Prev Chronic Dis* 2010;**7**:A109.

33 The CDC Diabetes Cost-effectiveness Group TCDC. Cost-effectiveness of Intensive Glycemic Control, Intensified Hypertension Control, and Serum Cholesterol Level

For peer review only - http://bmjopen.bmj.com/site/about/guidelines.xhtml

Reduction for Type 2 Diabetes. *JAMA* 2002;**287**:2542. doi:10.1001/jama.287.19.2542

34 Knowler WC, Barrett-Connor E, Fowler SE, *et al.* Reduction in the incidence of type 2 diabetes with lifestyle intervention or metformin. *N Engl J Med* 2002;**346**:393–403. doi:10.1056/NEJMoa012512

35 Selvin E, Steffes MW, Zhu H, *et al.* Glycated Hemoglobin, Diabetes, and Cardiovascular Risk in Nondiabetic Adults. *N Engl J Med* 2010;**362**:800–11. doi:10.1056/NEJMoa0908359

36 Kuo HS, Chang HJ, Chou P, *et al.* A Markov chain model to assess the efficacy of screening for non-insulin dependent diabetes mellitus (NIDDM). *Int J Epidemiol* 1999;**28**:233–40.

37 Icks A, Rathmann W, Haastert B, *et al.* Cost-Effectiveness of Type 2 Diabetes Screening: Results from Recently Published Studies. *Das Gesundheitswes* 2005;**67**:167–71. doi:10.1055/s-2005-858232

38 Roper NA, Bilous RW, Kelly WF, *et al.* Cause-specific mortality in a population with diabetes: South Tees Diabetes Mortality Study. *Diabetes Care* 2002;**25**:43–8.

39 Meigs JB, Muller DC, Nathan DM, *et al.* The natural history of progression from normal glucose tolerance to type 2 diabetes in the Baltimore Longitudinal Study of Aging. *Diabetes* 2003;**52**:1475–84.

40 Rasmussen SS, Glümer C, Sandbaek A, *et al.* Progression from impaired fasting glucose and impaired glucose tolerance to diabetes in a high-risk screening programme in general practice: the ADDITION Study, Denmark. *Diabetologia* 2007;**50**:293–7. doi:10.1007/s00125-006-0530-y

41 Wein P, Beischer N, Harris C, *et al.* A trial of simple versus intensified dietary modification for prevention of progression to diabetes mellitus in women with impaired glucose tolerance. *Aust N Z J Obstet Gynaecol* 1999;**39**:162–6.

42 Bonora E, Kiechl S, Willeit J, *et al.* Population-based incidence rates and risk factors for type 2 diabetes in white individuals: the Bruneck study. *Diabetes* 2004;**53**:1782–9.

For peer review only - http://bmjopen.bmj.com/site/about/guidelines.xhtml

43 Chiasson J-L, Josse RG, Gomis R, *et al.* Acarbose for prevention of type 2 diabetes mellitus: the STOP-NIDDM randomised trial. *Lancet* 2002;**359**:2072–7. doi:10.1016/S0140-6736(02)08905-5

44 de Vegt F, Dekker JM, Jager A, *et al.* Relation of impaired fasting and postload glucose with incident type 2 diabetes in a Dutch population: The Hoorn Study. *JAMA* 2001;**285**:2109–13.

45 Edelstein SL, Knowler WC, Bain RP, *et al.* Predictors of progression from impaired glucose tolerance to NIDDM: an analysis of six prospective studies. *Diabetes* 1997;**46**:701–10.

46 Eriksson J, Lindström J, Valle T, *et al.* Prevention of Type II diabetes in subjects with impaired glucose tolerance: the Diabetes Prevention Study (DPS) in Finland. Study design and 1-year interim report on the feasibility of the lifestyle intervention programme. *Diabetologia* 1999;**42**:793–801.

47 Eriksson J, Lehtovirta M, Ehrnstrom B, *et al.* Long-term beneficial effects of glipizide treatment on glucose tolerance in subjects with impaired glucose tolerance. *J Intern Med* 2006;**259**:553–60. doi:10.1111/j.1365-2796.2006.01633.x

48 Jarrett RJ, Keen H, Fuller JH, *et al.* Worsening to diabetes in men with impaired glucose tolerance ('borderline diabetes'). *Diabetologia* 1979;**16**:25–30.

49 Wareham NJ, Byrne CD, Williams R, *et al.* Fasting proinsulin concentrations predict the development of type 2 diabetes. *Diabetes Care* 1999;**22**:262–70.

50 DECODE study group. Glucose tolerance and mortality: comparison of WHO and American Diabetic Association diagnostic criteria. *Lancet* 1999;**354**:617–21. doi:10.1016/S0140-6736(98)12131-1

51 Rossing P, Hougaard P, Borch-Johnsen K, *et al.* Predictors of mortality in insulin dependent diabetes: 10 year observational follow up study. *BMJ* 1996;**313**:779–84.

52 Oizumi T. [Epidemiologic investigation on the incidence of diabetes mellitus from impaired glucose tolerance in population based study]. *Nihon Rinsho* 2005;**63 Suppl 2**:73–7.

For peer review only - http://bmjopen.bmj.com/site/about/guidelines.xhtml

53 Nakagami T, DECODA Study Group. Hyperglycaemia and mortality from all causes and from cardiovascular disease in five populations of Asian origin. *Diabetologia* 2004;**47**:385–94. doi:10.1007/s00125-004-1334-6

54 Orchard TJ, Temprosa M, Goldberg R, *et al.* The effect of metformin and intensive lifestyle intervention on the metabolic syndrome: the Diabetes Prevention Program randomized trial. *Ann Intern Med* 2005;**142**:611–9.

55 Fox CS, Pencina MJ, Meigs JB, *et al.* Trends in the Incidence of Type 2 Diabetes Mellitus From the 1970s to the 1990s: The Framingham Heart Study. *Circulation* 2006;**113**:2914–8. doi:10.1161/CIRCULATIONAHA.106.613828

56 Lakka H-M, Laaksonen DE, Lakka TA, *et al.* The metabolic syndrome and total and cardiovascular disease mortality in middle-aged men. *JAMA* 2002;**288**:2709–16.

57 Moss SE, Klein R, Klein BE. Cause-specific mortality in a population-based study of diabetes. *Am J Public Health* 1991;**81**:1158–62.

58 Fuller JH, Stevens LK, Wang SL. Risk factors for cardiovascular mortality and morbidity: the WHO Mutinational Study of Vascular Disease in Diabetes. *Diabetologia* 2001;**44 Suppl 2**:S54-64.

59 Tuomilehto J, Lindström J, Eriksson JG, *et al.* Prevention of Type 2 Diabetes Mellitus by Changes in Lifestyle among Subjects with Impaired Glucose Tolerance. *N Engl J Med* 2001;**344**:1343–50. doi:10.1056/NEJM200105033441801

60 Pan XR, Hu YH, Li GW, *et al.* Impaired glucose tolerance and its relationship to ECG-indicated coronary heart disease and risk factors among Chinese. Da Qing IGT and diabetes study. *Diabetes Care* 1993;**16**:150–6.

61 Harris MI, Klein R, Welborn TA, *et al.* Onset of NIDDM occurs at least 4-7 yr before clinical diagnosis. *Diabetes Care* 1992;**15**:815–9.

62 Pan X-R, Li G-W, Hu Y-H, *et al.* Effects of Diet and Exercise in Preventing NIDDM in People With Impaired Glucose Tolerance: The Da Qing IGT and Diabetes Study. *Diabetes Care* 1997;**20**.

63 Jia WP, Pang C, Chen L, *et al.* Epidemiological characteristics of diabetes mellitus and impaired glucose regulation in a Chinese adult population: the Shanghai

For peer review only - http://bmjopen.bmj.com/site/about/guidelines.xhtml

Diabetes Studies, a cross-sectional 3-year follow-up study in Shanghai urban communities. *Diabetologia* 2007;**50**:286–92. doi:10.1007/s00125-006-0503-1

64 Dunstan DW, Zimmet PZ, Welborn TA, *et al.* The rising prevalence of diabetes and impaired glucose tolerance: the Australian Diabetes, Obesity and Lifestyle Study. *Diabetes Care* 2002;**25**:829–34.

65 Diabetes Prevention Program Research Group, Knowler WC, Fowler SE, *et al.* 10-year follow-up of diabetes incidence and weight loss in the Diabetes Prevention Program Outcomes Study. *Lancet (London, England)* 2009;**374**:1677–86. doi:10.1016/S0140-6736(09)61457-4

66 Balk EM, Earley A, Raman G, *et al.* Combined Diet and Physical Activity Promotion Programs to Prevent Type 2 Diabetes Among Persons at Increased Risk: A Systematic Review for the Community Preventive Services Task Force. *Ann Intern Med* 2015;**163**:437–51. doi:10.7326/M15-0452

67 Morris DH, Khunti K, Achana F, *et al.* Progression rates from HbA1c 6.0–6.4% and other prediabetes definitions to type 2 diabetes: a meta-analysis. *Diabetologia* 2013;**56**:1489–93. doi:10.1007/s00125-013-2902-4

68 DECODE Study Group - European Diabetes Epidemiology. Is the current definition for diabetes relevant to mortality risk from all causes and cardiovascular and noncardiovascular diseases? *Diabetes Care* 2003;**26**:688–96.

69 Moody A, Cowley G, Fat LN, *et al.* Social inequalities in prevalence of diagnosed and undiagnosed diabetes and impaired glucose regulation in participants in the Health Surveys for England series. *BMJ Open* 2016;**6**:e010155. doi:10.1136/BMJOPEN-2015-010155

70 Healthcare Quality Improvement Partnership (HQIP). National Diabetes Audit, 2015-16 Report 2: Complications and Mortality. 2017.

71 National Cardiovascular Intelligence Network (NCVIN) - Public Health England. Prevalence estimates of diabetes. 2016.

For peer review only

For peer review only - http://bmjopen.bmj.com/site/about/guidelines.xhtml

Figure 1 - Review flowchart of epidemiological models

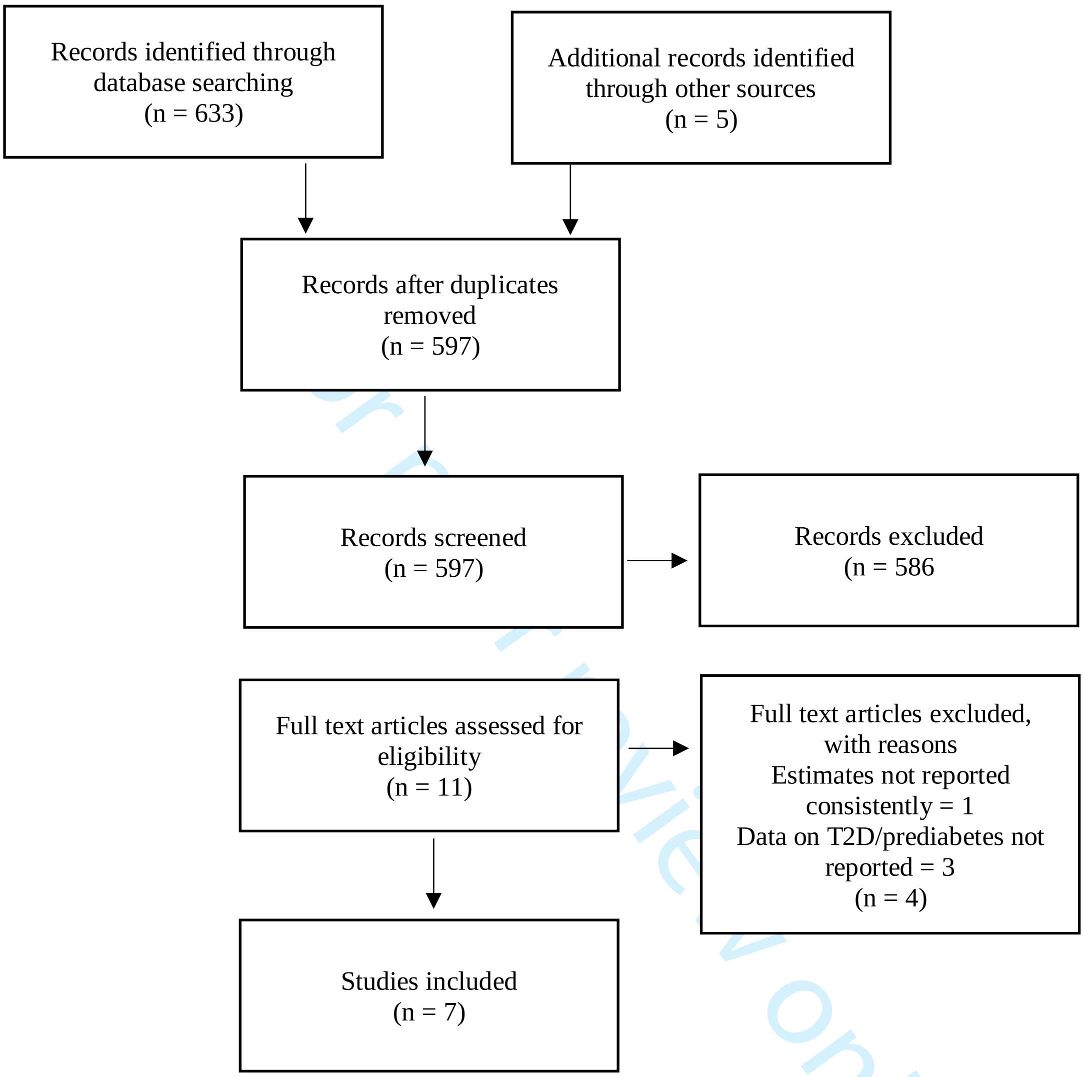

For peer review only - http://bmjopen.bmj.com/site/about/guidelines.xhtml

Figure 2 - Review flowchart of Markov chain models

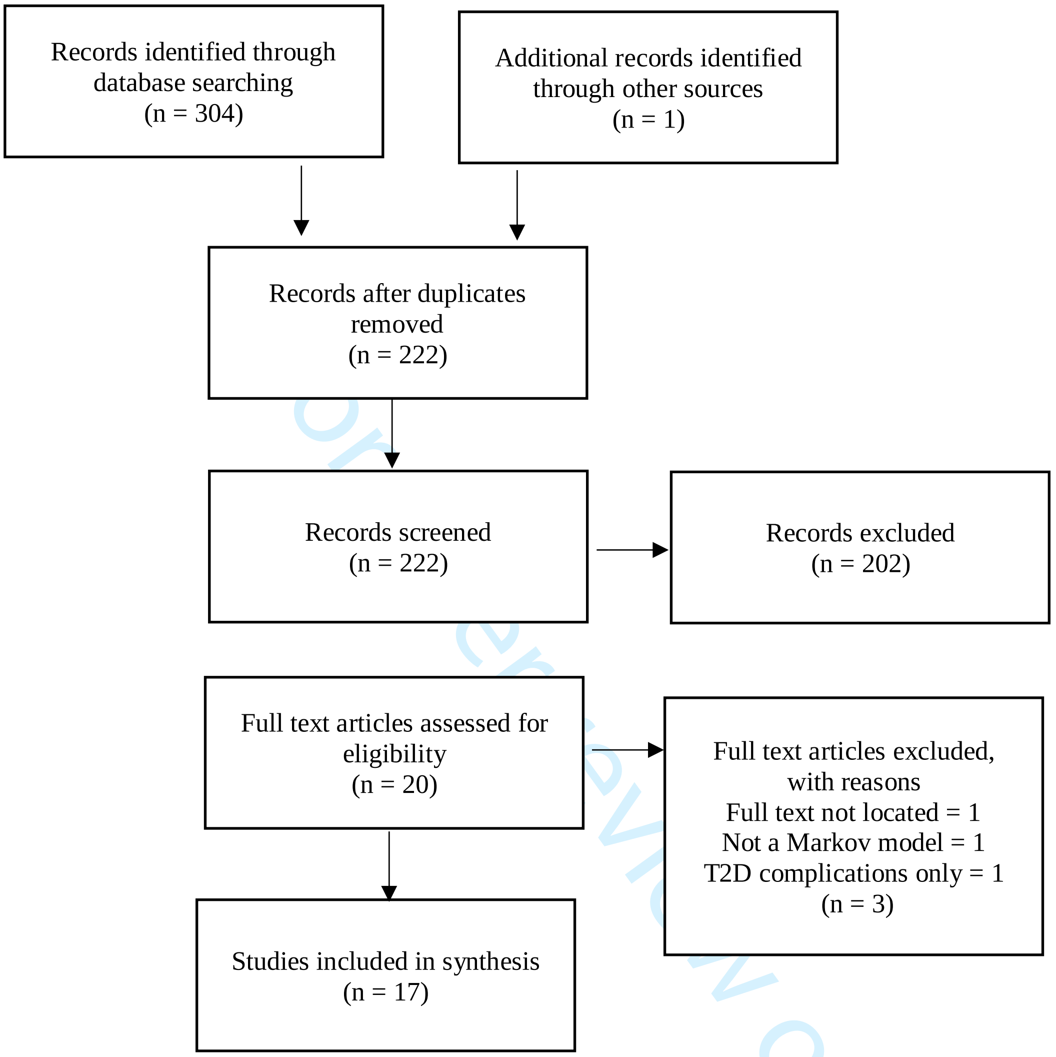

For peer review only

Figure 3 - Our Markov chain models

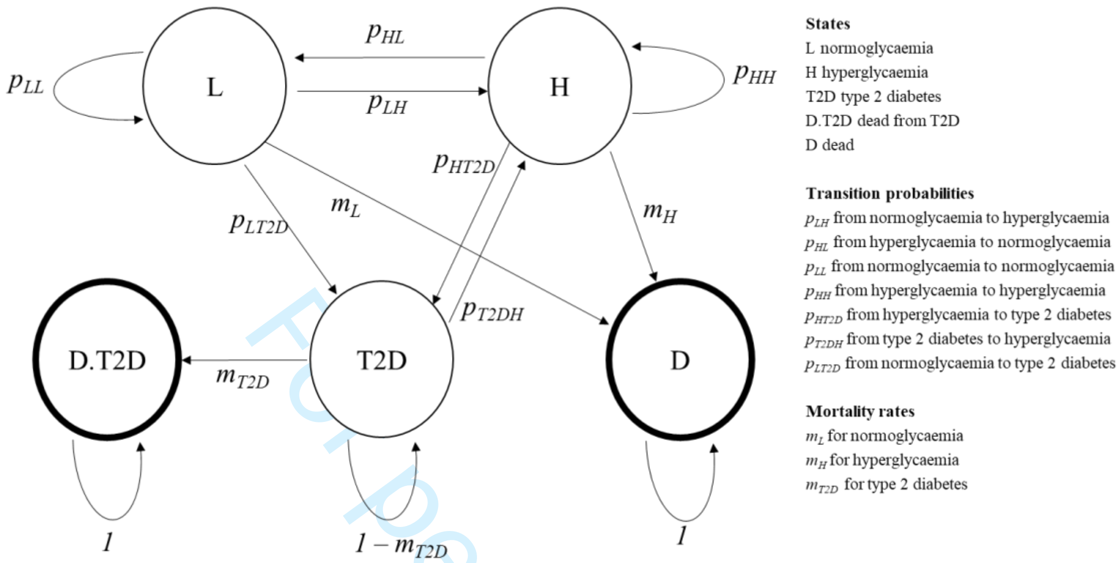

For peer review only

Figure 4 - Projections of true diabetes prevalence by PHE, Holman et al & from the QOF trend: 2005 to 2035

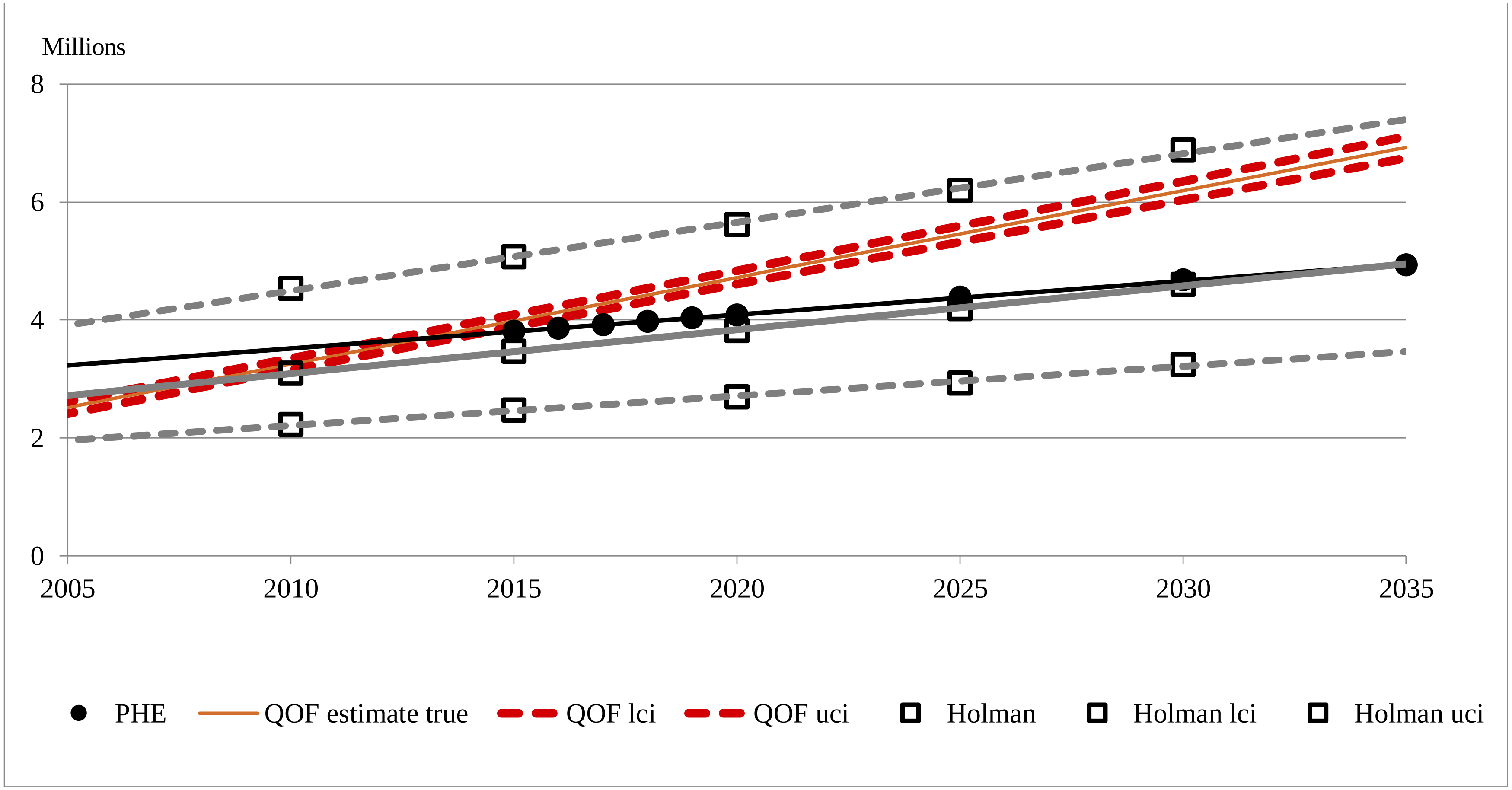

Figure 5 - Projections of adults with T2D in England: 2015 to 2035

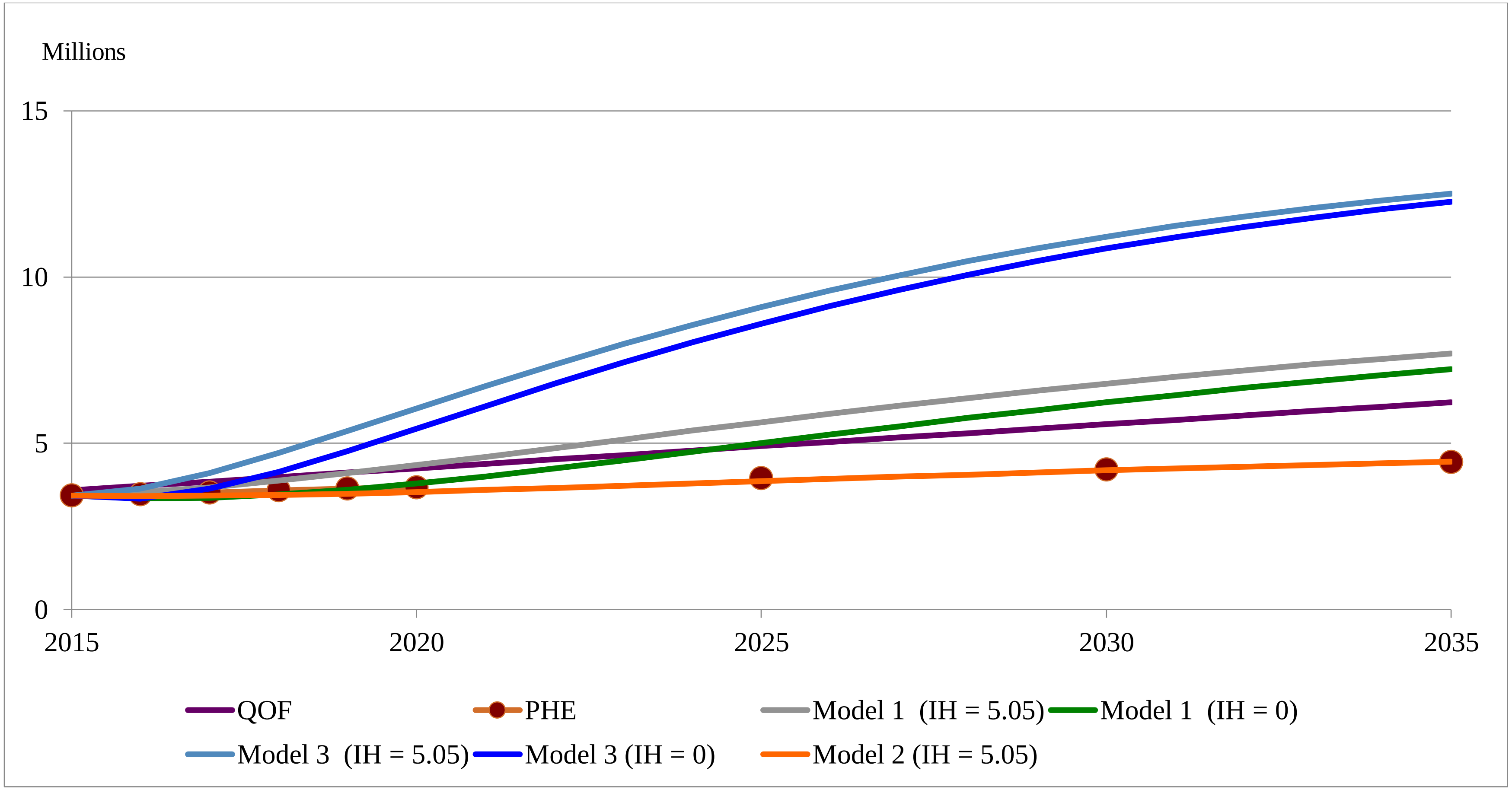

Appendix 1: Tables giving details of models

Index

Table 3.1: Measures of intermediate hyperglycaemia used in Markov chain models

Table 3.2: Data sources of estimates used by our Markov Chain models

Table 3.3: Ratios used for comparing different estimates

Table 3.4: The three sets of transition probabilities used in different models

For peer review only

Table 3.1: Measures of intermediate hyperglycaemia used in Markov chain models

Measure of intermediate hyperglycaemia	Definition
Impaired fasting glucose (IFG)	• Diagnosed with an Oral glucose tolerance test (OGTT) performed after an overnight fast<br>• Defined by a fasting plasma glucose (FPG) concentration of<br>○ 5.6-6.9 mmol/L according to American Diabetes Association (ADA)[1]<br>○ 6.0-6.9 mmol/L according to the World Health Organization (WHO)[2]
Impaired glucose tolerance (IGT)	• Diagnosed with a 2-hour glucose tolerance test (2hrGTT), i.e. a blood test performed 2 hours after a 75-g glucose load<br>• Defined by 2-h plasma glucose concentration of<br>○ 7.8-11 mmol/L according to to American Diabetes Association (ADA)[1]<br>○ 7-11 mmol/L according to the World Health Organization (WHO)[2]
Glycated Haemoglobin (HbA1c)	• Diagnosed with the A1c test, measuring the average blood glucose over 2-3 months<br>• Defined by A1c concentration of<br>○ 39-47 mmol/mol (5.7-6.4%) according to to American Diabetes Association (ADA)[1]<br>○ 42-47 mmol/mol (6.0-6.4%) according to the World Health Organization (WHO)[3]

For peer review only

Table 3.2: Data sources of estimates used by our Markov Chain models

Estimate	Year(s)	Source
Estimated prevalence of intermediate hyperglycaemia (based on HbA1c)	2015	Public Health England[4]
Estimated prevalence of diabetes (both types)	2015	Public Health England[5]
Estimated prevalence of normoglycaemia: residual of the population for 2015	2015	Office of National Statistics[6]
Age distributions for those with intermediate hyperglycaemia & diabetes	Five years of combined data from 2009 to 2013	Health Surveys for England (HSE)[7]
Mortality rates by age	2015	Office of National Statistics[6]
Hazard ratios for those with diabetes & T2D	2015-16	National Diabetes Audit[8]
Hazard ratios for those for those with intermediate hyperglycaemia	Various years	Systematic review[9]

For peer review only

Table 3.3: Ratios used for comparing different estimates

Numerator	Denominator	Ratio	Sources
Diagnosed prevalence of diabetes	True prevalence of diabetes	75%	[10,11]
Prevalence of T2D	Prevalence of diabetes	90%	[12]
English population aged 20 to 79 in 2030 & 2035	UK population aged 20 to 79 in 2030 & 2035	• 2030: 87%<br>• 2035: 87%	[6]
Prevalence of diabetics aged over 15 (England) in 2030 & 2035	Prevalence of diabetics aged 20 to 79 (England) in 2030 & 2035	• 2030: 128%<br>• 2035: 129%	[7]
Prevalence of diabetics aged over 15 in England in 2030 & 2035	Prevalence of diabetics aged over 20 in UK in 2030 & 2035	• 2030: 0.87*1.28 = 111%<br>• 2035: 0.87*1.29 = 113%	[6, 7]

For peer review only

Table 3.4: The three sets of transition probabilities used in different models

	Model 1*	Model 2**	Model 3***
Normoglycaemia – Normoglycaemia	0.925	0.925	0.831
Normoglycaemia – Intermediate hyperglycaemia	0.069	0.069	0.163
Normoglycaemia – T2D	0.000	0.000	0.000
Normoglycaemia – Dead	0.006	0.006	0.006
Total	*1.000*	*1.000*	*1.000*
Intermediate hyperglycaemia -Intermediate hyperglycaemia	0.856	0.878	0.754
Intermediate hyperglycaemia- Normoglycaemia	0.090	0.090	0.162
Intermediate hyperglycaemia – T2D	0.036	0.013	0.060
Intermediate hyperglycaemia – Dead	0.019	0.019	0.024
Totals	*1.000*	*1.000*	*1.000*
T2D-T2D	0.977	0.977	0.974
T2D – Normoglycaemia	0.000	0.000	0.000
T2D- Intermediate hyperglycaemia	0.000	0.000	0.005
T2D – Dead	0.023	0.023	0.021
Total	*1.000*	*1.000*	*1.000*

Notes:
* Model 1is based on the transition probabilities from Roberts et al[13]for HbA1c.
** Model 2 is based on Model 1 modified to generate the PHE projections of the prevalence of T2D:the transition probability from intermediate hyperglycaemia to T2D of Model 2 (0.013) is a third of that of Model 1 (0.036); and has a corresponding increase in the transition probability of remaining as intermediate hyperglycaemia (0.836 to 0.878).
*** Model 3 is based on the transition probabilities from Neuman et al[14] for IGT.

References

1 American Diabetes Association (ADA). 2. Classification and Diagnosis of Diabetes: Standards of Medical Care in Diabetes. *Diabetes Care* 2018;**41**:S13–27.

2 World Health Organization (WHO). Definition and diagnosis of diabetes mellitus and intermediate hyperglycemia : report of a WHO/IDF consultation. Geneva (CH): 2006.

3 World Health Organization (WHO). Use of glycated haemoglobin (HbA1c) in the diagnosis of diabetes mellitus. 2011.

4 Public Health England - National Cardiovascular Intelligence Network. Prevalence estimates of non-diabetic hyperglycaemia. 2015.

5 National Cardiovascular Intelligence Network (NCVIN) - Public Health England. Prevalence estimates of diabetes. 2016.

6 Office for National Statistics. 2014-based National Population Projections. 2015.

7 National Cardiovascular Intelligence Network (NCVIN)- Public Health England. NHS Diabetes Prevention Programme (NHS DPP) Non-diabetic hyperglycaemia. 2015.

8 Healthcare Quality Improvement Partnership (HQIP). National Diabetes Audit, 2015-16 Report 2: Complications and Mortality. 2017.

9 Huang Y, Cai X, Mai W, *et al.* Association between prediabetes and risk of cardiovascular disease and all cause mortality: systematic review and

For peer review only - http://bmjopen.bmj.com/site/about/guidelines.xhtml

meta-analysis. *BMJ* 2016;**355**:i5953. doi:10.1136/bmj.i5953

10 Holman N, Forouhi NG, Goyder E, *et al.* The Association of Public Health Observatories (APHO) Diabetes Prevalence Model: Estimates of total diabetes prevalence for England, 2010-2030. *Diabet Med* 2011;**28**:575–82. doi:10.1111/j.1464-5491.2010.03216.x

11 Moody A, Cowley G, Fat LN, *et al.* Social inequalities in prevalence of diagnosed and undiagnosed diabetes and impaired glucose regulation in participants in the Health Surveys for England series. *BMJ Open* 2016;**6**:e010155. doi:10.1136/BMJOPEN-2015-010155

12 Gatineau M, Hancock C, Holman N, *et al.* Adult obesity and type 2 diabetes. London, UK: 2014.

13 Roberts S, Craig D, Adler A, *et al.* Economic evaluation of type 2 diabetes prevention programmes: Markov model of low- and high-intensity lifestyle programmes and metformin in participants with different categories of intermediate hyperglycaemia. *BMC Med* 2018;**16**:16. doi:10.1186/s12916-017-0984-4

14 Neumann A, Lindholm L, Norberg M, *et al.* The cost-effectiveness of interventions targeting lifestyle change for the prevention of diabetes in a Swedish primary care and community based prevention program. *Eur J Heal Econ* 2017;**18**:905–19. doi:10.1007/s10198-016-0851-9

For peer review only

For peer review only - http://bmjopen.bmj.com/site/about/guidelines.xhtml

Appendix 2: Rapid Reviews of Epidemiological and Markov chain models

Table 1 gives the search strategies for each review and Table 2 the details of our rapid review of Markov chain models

Table 1: Search strategies for each review

**Epidemiological models**	
**Web of science**	TOPIC: ("diabet*" OR "type 2 diabetes" OR "diabetes mellitus") AND TITLE: ("Engl*" or "United Kingdom" or "UK") AND TOPIC: ("model" or "simulation" or "project*") AND TOPIC: ("epidemiolog*" or "prevalence" or "incidence" or "trend*") NOT TITLE: ("child*")<br>Timespan: All years.<br>Search language=Auto
**PubMed**	(((("diabet*"[All Fields] OR "type 2 diabetes"[All Fields] OR "diabetes mellitus"[All Fields] OR "pre-diabetes"[All Fields] OR "prediabetes"[All Fields]) AND ("economic evaluation"[All Fields] OR "cost-effectiveness"[All Fields] OR "cost effectiveness"[All Fields] OR "cost-utility"[All Fields] OR "cost utility"[All Fields])) AND "markov"[All Fields]) NOT ("child*"[All Fields] OR "pediatric"[All Fields] OR "paediatric"[All Fields])) NOT "type 1 diabetes"[All Fields] AND ("humans"[MeSH Terms] AND English[lang])
**Markov chain models**	
**Web of science**	TITLE: ("diabet*" OR "type 2 diabetes" OR "diabetes mellitus" or "pre-diabetes" or "prediabetes") AND TITLE: ("economic evaluation" or "cost-effectiveness" or "cost effectiveness" or "cost-utility" or "cost utility") NOT TOPIC: ("child*" or "pediatric" or "paediatric") NOT TOPIC: ("type 1 diabetes") AND TOPIC: ("markov")<br>Refined by: LANGUAGES: (ENGLISH)<br>Timespan: All years.<br>Search language=Auto
**PubMed**	(((("diabet*"[All Fields] OR "type 2 diabetes"[All Fields] OR "diabetes mellitus"[All Fields] OR "pre-diabetes"[All Fields] OR "prediabetes"[All Fields]) AND ("economic evaluation"[All Fields] OR "cost-effectiveness"[All Fields] OR "cost effectiveness"[All Fields] OR "cost-utility"[All Fields] OR "cost utility"[All Fields])) AND "markov"[All Fields]) NOT ("child*"[All Fields] OR "pediatric"[All Fields] OR "paediatric"[All Fields])) NOT "type 1 diabetes"[All Fields] AND ("humans"[MeSH Terms] AND English[lang])

For peer review only

Table 2: Details of our rapid review of Markov chain models

Author	Type	Country	Risk measure	Objectives of the model	Description of the Markov model	Transition probabilities	Mortality rates	Population modelled	Outcomes	Results in terms of the number of cases prevented under "no intervention"	Sensitivity analysis	Model validation
Caro et al 2004	EA	Canada	IGT	- To compare the health and economic outcomes of acarbose, an intensive lifestyle modification programme, metformin or no intervention to prevent progression to diabetes	A Markov model to simulate long-term outcomes in a cohort of patients with IGT under each of four treatment strategies. The cohort is followed for a 10- year period in the base case analyses. The model cycles over 6-month periods. Four main states were considered: IGT, diabetes, normal glucose tolerance (NGT) and death. Patients who revert to NGT may develop IGT again, while patients who develop diabetes are assumed to remain in that state until death.	Reported, originally developed for the model	Estimated based on age- and gender-specific death hazards calculated from Canadian life table data and increased by 45% to take into account the effect of IGT. Upon reverting to NGT, patients were assumed to lose the increased risk.	For the base case, patient characteristics were taken from the STOP-NIDDM trial [12]. Just over half of patients in that trial were male, and the mean age at the start of the trial was 54.5 years.	No of patients transitioning to T2D<br>No who reverted and remained NGT<br>Life expectancy<br>Years free of T2D	For a cohort of 1000 patients, over the course of 10 years, 542 untreated patients with IGT are expected to develop diabetes, while 242 will have returned to NGT	Performed, results for base case not available	Not available
Chen et al, 2001	CEA	Taiwan	NA	- To develop the natural history of T2D<br>- To quantify the efficacy of early detection of T2D in slowing or reducing the progression of complications<br>- To evaluate the effect of inter-screening interval and age at the start of screening on slowing/reducing the progression of complications or deaths<br>- To compare the cost and effectiveness of a screening regime<br>- To assess the cost–effectiveness of T2D screening by age-specific groups and different inter-	A Markov model to simulate the natural history of T2D from normal, onset of DM, clinical complications, deaths.<br>Disease progression modules from onset of DM to complications include three parts: Retinopathy, Nephropathy, and Neuropathy.	Not reported<br>Transition parameters used for simulating disease progression refer to Eastman et al., Javitt at al., Harris et al., Klein et al., Ballard et al., Humphrey et al. , USRD, Dyck et al., Humphrey et al., and CDC–DCS group.	Not provided<br>- Life-table information was used to adjust for competing causes of deaths<br>- Mortality rates for diabetes complications retrieved from the literature	A hypothetical cohort with 30,000 adults aged over 30	Life-years gained<br>QALYs	Not available	Not available	Not available

For peer review only

Author	Type	Country	Risk measure	Objectives of the model	Description of the Markov model	Transition probabilities	Mortality rates	Population modelled	Outcomes	Results in terms of the number of cases prevented under "no intervention"	Sensitivity analysis	Model validation
				screening interval.								
Gillies et al, 2008	CEA	UK	IGT	To compare potential screening strategies, and subsequent interventions, for the prevention and treatment of T2D<br>- screening for T2D to enable early detection and treatment<br>- screening for T2D and impaired glucose tolerance, intervening with lifestyle interventions in those with a diagnosis of impaired glucose tolerance<br>- as for (b) but with pharmacological interventions<br>- no screening	Hybrid model consists of a decision tree and a Markov model<br>The decision tree comprises three main arms, representing no screening, screening for undiagnosed T2D, and screening for impaired glucose tolerance and undiagnosed diabetes, with either lifestyle or pharmacological interventions applied in those with impaired glucose tolerance<br>The Markov model consists of seven states: normal glucose tolerance, undiagnosed impaired glucose tolerance, diagnosed impaired glucose tolerance, death, and three states for people with diabetes (undiagnosed, diagnosed clinically, or diagnosed through screening, either from a screening test or because they are diagnosed with impaired glucose tolerance initially and hence enter a surveillance programme)<br>Each model cycle represents one year and the model is run for a time horizon of 50 years.	Reported	Increased risk of death with diabetes<br>(hazard ratio) 0.756 SE - (0.087)<br>Increased risk of death for 1% increase in HbA1c (hazard ratio) 0.104 (SE =0.039	Hypothetical population, aged 45 at time of screening, with above average risk of diabetes..	Clinical and cost outcomes	Not available	Performed, results available	Not available

For peer review only

Author	Type	Country	Risk measure	Objectives of the model	Description of the Markov model	Transition probabilities	Mortality rates	Population modelled	Outcomes	Results in terms of the number of cases prevented under "no intervention"	Sensitivity analysis	Model validation
Herman et al, 2005	CEA	USA	IGT	To estimate the lifetime cost–utility of the DPP interventions.	Markov model originally developed by the Centers for Disease Control and Prevention and Research Triangle Institute International to assess the progression from impaired glucose tolerance to onset of diabetes to clinically diagnosed diabetes to diabetes with complications and death by using a lifetime simulation model.	Not reported	Not reported	Members of the DPP cohort 25 years of age or older with impaired glucose tolerance.	Progression of disease<br>Costs<br>Quality of life	If the entire DPP cohort were treated with the placebo intervention, approximately 50% of individuals would develop diabetes within 7 years. Over a lifetime conversion rate from IGT to T2D is 82.8%	Performed, results available	Not available
Ikeda et al, 2010	CEA	Japan	IGT	To estimate the cost-effectiveness of administering voglibose, in addition to standard care of diet and exercise, compared with standard care alone for high-risk Japanese patients with impaired glucose tolerance	Markov model consisting of five stages: normal glucose tolerance, IGT, T2DM, dialysis and death	Available only for transition from NGT to IGT	For the annual mortality of NGT, the average values for males and females in the national data of the abridged life table in 2008 were used Relative risk of death in IGT and T2DM in comparison with NGT was set as 1.35 and 3.03, respectively.	The age of the IGT population was set as 56, corresponding to the average age in the voglibose clinical trial population,	- Long-term costs<br>- Life expectancy<br>- Cost-effectiveness	Not available	Performed, results available	Not available
Johansson et al, 2009	CEA	Sweden	FPG	To estimate the cost-effectiveness of a community-based program promoting general population lifestyle changes to prevent diabetes.	Markov model constructed to reflect the metabolic syndrome, covers adults, with the termination age set at 85 years, after which no further health effects or costs are accumulated<br>Model is fully described in a separate technical report	Not reported	Not reported<br>Mortality risks were taken from Swedish registers, and included both disease-related mortality as well as unrelated mortality	Population group aged 36–56 years at baseline	- Costs<br>- QALYs	Not available	Performed, results available	Not available
Liu et al, 2013	EA	China	IGT	To estimate the clinical and economic outcomes of screening for undiagnosed diabetes and impaired glucose tolerance (IGT), followed by the implementation of lifestyle intervention in those with IGT.	Hybrid decision tree Markov model. The decision tree included five arms representing five scenarios. The first three scenarios involved screening for undiagnosed diabetes and IGT followed by one of the three active lifestyle interventions (diet, exercise or duo-intervention), which	Reported	Not reported<br>The life-table information was used to evaluate the competing causes of death at the different initiation ages	A representative sample of Chinese adults was used to create a simulated population of 20,000 people aged 25 years and above.	- Remaining survival years QALYs per subject with diabetes or IGT<br>- Life-years gained before the onset of diabetes or before the onset of any complication per subject with IGT<br>- Cost per subject for prevention strategies or	Not available	Performed, results available.	Performed, not reported

For peer review only

Author	Type	Country	Risk measure	Objectives of the model	Description of the Markov model	Transition probabilities	Mortality rates	Population modelled	Outcomes	Results in terms of the number of cases prevented under "no intervention"	Sensitivity analysis	Model validation
					were applied to the IGT subjects. The fourth scenario involved screening for undiagnosed diabetes and IGT, without the formal lifestyle interventions. The fifth scenario involved the control group with no screening or intervention.<br><br>The decision tree used positive screening rates and the prevalence of diabetes and IGT in the reference population to determine how many individuals started in each state of the Markov models. Each Markov model consisted of eight main health states: IGT, normal glucose tolerance, onset of diabetes, four diabetes complication states and death. The Markov models ran for a time horizon of 40 years, and each of the model cycles represented 1 year. Separate simulations with different incidence rates of diabetes, mortality rates and health utilities were performed for the diabetes prevention programmes or for the control starting at 25, 40 and 60 years, respectively.				control at different initiation ages.			
Neumann et al, 2011	CEA	Germany	IGT	To investigate the long-term cost-effectiveness of lifestyle intervention programmes for the prevention of T2D	Four-state Markov modelling with a probabilistic cohort analysis : normal glucose tolerance (NGT), impaired glucose tolerance	Reported	Not reported Mortality Life tables provide the mortality rates for different ages and sexes. Eight different mortality categories, by age and	The prevalence of IGT among the general German population is used as the base for the model, with 16% of individuals having	- Cost per quality-adjusted life year (QALY)	Not available	Performed, results available	Not available

For peer review only

Author	Type	Country	Risk measure	Objectives of the model	Description of the Markov model	Transition probabilities	Mortality rates	Population modelled	Outcomes	Results in terms of the number of cases prevented under "no intervention"	Sensitivity analysis	Model validation
					(IGT), diagnosed type 2 diabetes mellitus (T2D), or death. A one-year cycle length and a lifetime time horizon are applied.		sex, are established: less than 35, 35-64, 65-74, and 75 years and over for men and women. Mortality statistics were obtained from the Statistical Office of the Federal State of Saxony Transition probability of a person with T2D dying from T2D. adjusted using the data on all-cause deaths attributable to diabetes from the study by Roglic et al [25].	IGT, 84% NGT and no one T2D.				
Neumann et al, 2017	CEA	Sweden	IFG IGT	To estimate the cost-effectiveness of a T2D prevention initiative targeting weight reduction, increased physical activity and healthier diet in persons in pre-diabetic states by comparing a hypothetical intervention versus no intervention in a Swedish setting.	The model consisted of six different, mutually exclusive states: NGT, IFG, IGT, IFG and IGT, T2D and death. The length of one cycle was 1 year. A lifetime horizon was applied.<br><br>As it was assumed that 1 year was too short to develop T2D directly from NGT, this transition was not possible. Hence, all hypothetical persons must have developed any of the three pre-diabetic states before the development of T2D.	Not reported	Age-based all-cause mortality and mortality due to T2D were taken from Statistics Sweden and the National Board of Health and Welfare based on the years 2003–2009 Five-year age ranges were estimated No increased risk of dying due to any of the pre-diabetic states was assumed.	Not reported Based on the Vasterbotten Intervention Program (VIP)	- QALY<br>- Incremental cost-effectiveness ratios (ICERs)	Not available	Performed, results available	Not available
Palmer et al, 2012	CEA	Australia	IGT	To examine the long-term cost-effectiveness of the control, metformin and ILC interventions in the DPP for a cohort of subjects at high risk of developing type 2 diabetes in an Australian healthcare setting	Semi-Markov model, with four health states: 'normal glucose regulation' (NGR) (plasma glucose con-centration <5.6 mmol/L in fasting state or <7.8 mmol/L 2 h after a 75 g oral glucose load); 'impaired glucose tolerance' (IGT) (fasting plasma glucose concentration 5.6–6.9 mmol/L or 7.8–11.0 mmol/L 2 h after a 75 g oral	Reported	Annual mortality rates were calculated from Australian sex- and age-specific life tables and were state-dependent, but independent of treatment arm All-cause mortality rates in the NGR state were applied unadjusted Relative mortality risks for subjects in the IGT, "undiagnosed" diabetes or "diagnosed" diabetes	A hypothetical cohort was defined with baseline characteristics in keeping with the Diabetes Prevention Program (DPP) study: mean age 50.6 years; 32.2% male; mean body mass index 34.0 kg/m2; and IGT present.	- Cumulative incidence<br>- Lifetime incremental direct costs<br>- Incremental costs per QALY-gained	Mean cumulative incidence (95% CI) of type 2 diabetes in the control, metformin and ILC treatment arms estimated at 89.7% (89.4–90.1), 83.8% (83.3–84.3) and 73.4 (72.8–74.1), respectively	Performed, results available	Internal validation performed, results available

For peer review only

Author	Type	Country	Risk measure	Objectives of the model	Description of the Markov model	Transition probabilities	Mortality rates	Population modelled	Outcomes	Results in terms of the number of cases prevented under "no intervention"	Sensitivity analysis	Model validation
					glucose load); 'type 2 diabetes' (T2D) (plasma glucose concentration at least 7.0 mmol/L or 11.1 mmol/L 2 h after a 75 g oral glucose load),'dead'. Each cycle in the model represented one year of a simulated subject's life and at the end of each cycle, subjects could remain in the same state, progress to another state or die. The simulation ran over subject lifetimes		states were 1.50 (95% CI 1.10–2.00), 1.30 (0.90–2.66) and 2.30 (1.60–3.20), respectively					
Palmer et at, 2004	EA	Australia France Germany Switzerland UK	IGT	To establish whether implementing the active treatments used in the DPP would be cost-effective in the selected countries.	Markov model consisting of 3 states: IGT (as defined in the DPP), type 2 DM, and deceased. Simulated patients initially had IGT and progressed at differing rates to T2S depending on the treatment received. A patient lifetime horizon was used.	Reported	Partially reported The probability of death associated with IGT or T2D was calculated from age- and sex-dependent country-specific, national, all-cause mortality tables, adjusted using published relative risks (RRs) for all-cause mortality for patients with IGT versus normo-glycemic patients.	The cohort of patients in this analysis was constructed to resemble the study population of the DPP (mean age, 50.6 years; mean body weight, 94.2 kg; mean body mass index [BMI], 34.0 kg/m2; men, 32.2%)	- No of years free of DM<br>- Percentage of patients developing DM<br>- Life expectancy<br>- Total lifetime costs per patient	Not available	Performed, results available	Not available
Roberts et al, 2018	EA	England	IFG IGT HbA1c	To examine the costs and effects of different intensity lifestyle programmes and metformin in participants with different categories of intermediate hyperglycaemia	Decision tree and Markov model (50-year horizon) to compare four approaches: (1) a low-intensity lifestyle programme based on current NICE guidance, (2) a high-intensity lifestyle programme based on the US Diabetes Prevention Program, (3) metformin, and (4) no intervention, modelled for three different types of intermediate hyperglycaemia (IFG, IGT and HbA1c).	Reported	Not reported All-cause age-standardised mortality rates were determined from the Office of National Statistics in England, with increased risk of death calculated for participants with intermediate hyper-glycaemia or T2DM	Not described	Impact on an individual participant in a prevention programme: (1) discounted cumulative healthcare costs (including costs of diagnostic tests and primary and secondary care associated with the intervention, intermediate hyperglycaemia, T2DM and complications of T2DM), (2) discounted QALYs, (3) incidence of T2DM, (4) average number of years with T2DM, (5) cost-effectiveness ratios in £/QALY, and (6)	With no intervention, 42% of the IGT population and 38% of the IFG and HbA1c population developed T2DM over 50 years.	Performed, results available	Performed, reported

For peer review only - http://bmjopen.bmj.com/site/about/guidelines.xhtml

Author	Type	Country	Risk measure	Objectives of the model	Description of the Markov model	Transition probabilities	Mortality rates	Population modelled	Outcomes	Results in terms of the number of cases prevented under "no intervention"	Sensitivity analysis	Model validation
									incremental cost-effectiveness ratios (ICERs), in £/QALY (for non-dominated interventions). Impact of a nation-wide prevention programme: (1) discounted annual incremental costs, (2) discounted cumulative incremental costs, (3) discounted incremental costs as a percentage of the total diabetes expenditure [17], and (4) cumulative incidence of T2DM.			
Schaufler et al, 2010	CEA	Germany	OGTT	To examine the cost effectiveness of screening for T2DM in Germany	Markov model to reproduce the time-discrete stochastic process using a 1 year cycle	Reported	Not reported<br>General mortality rates were derived from the official German mortality tables<br>The higher mortality for patients with diabetes than for the general population, especially after cardio-vascular events, was accounted for by applying relative risks drawn from Roper et al.[28]	Not described	- Quality of life (QOL)<br>- Lifetime costs<br>- Age at diabetes diagnosis<br>- Incidence and age at occurrence of diabetes-related complications.	Not available	Performed, results available	Performed, reported
Smith et al 2010	CEA	USA	IFG	To assessed the cost-effectiveness of a modified version of the US DPP	Markov model with six states: risk factor negative (no diabetes), risk factor positive (enrolled in mDPP), risk factor positive (not enrolled in mDPP), stable T2D, complications, death	Partially reported	Mortality rates based on age- and sex-specific US mortality (which accounts for baseline mortality) and the relative risks of death for metabolic syndrome, stable diabetes, and complicated diabetes<br>- risk factor positive 1.7 (Lakka et al 2002)<br>- risk factor negative 1 (own assumption)<br>- stable T2D 2 (Moss et al 1991)	In the model, we used a base case that examined 55-year-old men and women at monthly intervals for 3 years.<br>75% women	- Metabolic syndrome risk at 1 year<br>- Costs<br>- QALYs<br>- T2D incidence	Without the mDPP, 9.6% of the cohort developed diabetes over 3 years	Performed, results for base-case not reported	Not available

For peer review only

Author	Type	Country	Risk measure	Objectives of the model	Description of the Markov model	Transition probabilities	Mortality rates	Population modelled	Outcomes	Results in terms of the number of cases prevented under "no intervention"	Sensitivity analysis	Model validation
							- complicated T2D 2.4 (Fuller et al 2001)					
Wong et l, 2016	CEA	Hong Kong	IGT	To investigate the costs and cost-effectiveness of a short message service (SMS) intervention to prevent the onset of type 2 diabetes mellitus (T2DM) in subjects with impaired glucose tolerance (IGT).	Markov model with one-year transition cycle with four Markov states: normal glucose tolerance (NGT), IGT, T2DM, and death. Long-term modelling referred to time horizon over a 50-year period beyond the two year intervention	Reported	All-cause mortality rates for NGT were adopted from the Hong Kong Life Table 2011<br>The relative risks of mortality in IGT and T2DM were 1.50 (95% CI 1.10–2.00) and 2.30 (95%CI 1.60–3.20), respectively, which were used to adjust the age-specific death rate for subjects with IGT or T2DM [23]	Not reported	- Costs<br>- QALYs	Not available	Performed, results available	Not available
Zhou et al. 2005	CEA	USA	IGT	To develop and validate a comprehensive computer simulation model to assess the impact of screening, prevention, and treatment strategies on T2D and its complications, comorbidities, quality of life, and cost.	Markov model with four states: NGT, IGT, T2D, death.	Not reported	Not reported<br>Estimates of mortality are taken from the age-, sex-, and race-specific mortality for the U.S. population.	Not reported	- Health states<br>- Utilities<br>- Costs	Not available	Not available	Validated usning the WESDR is a population- based study of individuals with diabetes the WESDR cohort with type 2 diabetes in southern Wisconsin.
Zhuo et al, 2012	CEA	USA	HbA1c	To examine the change in the cost effectiveness of diabetes-preventive interventions because of progressive 0.1% decremental reductions in the HbA1c cutoff from 6.4% to 5.5%.	Markov model described in a report by Herman et al.	Not reported	Not reported	A nationally representative sample of U.S. adults (aged 18 years) from the 1999–2006 National Health and Nutrition Examination Survey (NHANES)	Cost effectiveness associated with the HbA1c cutoffs was measured as cost per QALY gained	Not available	Performed, results available	Performed, not reported. International

For peer review only

BMJ Open

How valid are projections of the future prevalence of diabetes? Rapid reviews of epidemiological and Markov chain models and comparisons of different models' projections for England

Journal:	*BMJ Open*
Manuscript ID	bmjopen-2019-033483.R1
Article Type:	Original research
Date Submitted by the Author:	16-Oct-2019
Complete List of Authors:	Bevan, Gwyn; London School of Economics and Political Science, Management<br>De Poli, Chiara; London School of Economics and Political Science, Management<br>Keng, Mi; University of Oxford, Nuffield Department of Population Health<br>Raine, Rosalind; University College London, Department of Applied Health Research
**Primary Subject Heading**:	Diabetes and endocrinology
Secondary Subject Heading:	Epidemiology, Health economics, Health services research, Health policy, Public health
Keywords:	Diabetes Mellitus, Prevalence, Markov Chains, Obesity, EPIDEMIOLOGY

SCHOLARONE™
Manuscripts

BMJ

I, the Submitting Author has the right to grant and does grant on behalf of all authors of the Work (as defined in the below author licence), an exclusive licence and/or a non-exclusive licence for contributions from authors who are: i) UK Crown employees; ii) where BMJ has agreed a CC-BY licence shall apply, and/or iii) in accordance with the terms applicable for US Federal Government officers or employees acting as part of their official duties; on a worldwide, perpetual, irrevocable, royalty-free basis to BMJ Publishing Group Ltd ("BMJ") its licensees and where the relevant Journal is co-owned by BMJ to the co-owners of the Journal, to publish the Work in this journal and any other BMJ products and to exploit all rights, as set out in our licence.

The Submitting Author accepts and understands that any supply made under these terms is made by BMJ to the Submitting Author unless you are acting as an employee on behalf of your employer or a postgraduate student of an affiliated institution which is paying any applicable article publishing charge ("APC") for Open Access articles. Where the Submitting Author wishes to make the Work available on an Open Access basis (and intends to pay the relevant APC), the terms of reuse of such Open Access shall be governed by a Creative Commons licence – details of these licences and which Creative Commons licence will apply to this Work are set out in our licence referred to above.

Other than as permitted in any relevant BMJ Author's Self Archiving Policies, I confirm this Work has not been accepted for publication elsewhere, is not being considered for publication elsewhere and does not duplicate material already published. I confirm all authors consent to publication of this Work and authorise the granting of this licence.

For peer review only

How valid are projections of the future prevalence of diabetes? Rapid reviews of epidemiological and Markov chain models and comparisons of different models' projections for England

Gwyn Bevan*

emeritus professor of policy analysis

Department of Management, London School of Economics and Political Science, WC2A 2AE, London, UK.

Email: R.G.Bevan@lse.ac.uk

Mob: +44 (0)77867 88967

Chiara De Poli

Research fellow

Department of Management, London School of Economics and Political Science, WC2A 2AE, London, UK.

Mi Jun Keng

researcher

Nuffield Department of Population Health, University of Oxford, OX3 7LF, Oxford, UK.

Rosalind Raine

professor of applied health research

Department of Applied Health Research, UCL, WC1E 7HB, London, UK

* Corresponding author

For peer review only

How valid are projections of the future prevalence of diabetes? Rapid reviews of epidemiological and Markov chain models and comparisons of different models' projections for England

Abstract

Objectives

To examine validity of epidemiological models giving projections of prevalence of diabetes in adults, in England and the UK, and of Markov chain models giving estimates of impacts of interventions to prevent type 2 diabetes (T2D).

Methods

Rapid reviews of epidemiological and Markov chain models. Estimation of the future prevalence of T2D in England: by Markov chain models; and from the trend in the prevalence of diabetes as recorded in the Quality and Outcomes Framework (QOF) estimated by Ordinary Least Squares (OLS) regression analysis.

Setting

Adult population in England and UK.

Main outcome measure

Prevalence of T2D in 2025.

For peer review only

Results

The epidemiological models reviewed use sample estimates of past prevalence rates by age and sex and projected population changes. Three most recent models, including that of Public Health England (PHE), neither take account of increases in obesity, nor report confidence intervals.

The Markov chain models reviewed use transition probabilities between states of risk and death, estimated from various sources, to give projected impacts of the preventive interventions on the numbers of adults who go on to develop T2D. None of their accounts give the full matrix of transition probabilities, nor report tests of validation of their models' estimates of the impacts of preventive interventions on prevalence of T2D at the population level.

Projections of the prevalence of T2D in England in 2025 were (in millions, with 95% confidence intervals where available) by PHE, 3.95; from the QOF trend, 4.91 (4.79 to 5.03); and by our two Markov chain models, 5.64 and 9.10.

Conclusions

Governments require realistic projections of the future prevalence of T2D from epidemiological models that take account of increases in obesity; and estimates of the likely relative impacts of preventive interventions from models that have been validated against projections from realistic epidemiological models.

Article summary

Strengths and limitations of this study

- We undertook rapid reviews of epidemiological models and Markov chain models, which have been used to give projections of the future prevalence of diabetes to examine their data sources and assumptions.
- We compared projections of the future prevalence of diabetes in England from: reports for the epidemiological models; our own Markov chain models

For peer review only - http://bmjopen.bmj.com/site/about/guidelines.xhtml

(which used transition probabilities from our review); and the trend in the prevalence of diagnosed diabetes as reported by general practitioners in England (estimated by ordinary least squares regression analysis).

- This study's limitations are that our reviews were rapid and our models are transparent and simple.

Keywords

Diabetes Mellitus

Prevalence

Forecasting

Markov Chains

Obesity

3,595 words

For peer review only - http://bmjopen.bmj.com/site/about/guidelines.xhtml

How valid are projections of the future prevalence of diabetes? Rapid reviews of epidemiological and Markov chain models and comparisons of different models' projections for England

Introduction

Rigorous analysis of worldwide trends of increases in the preventable onset of Type 2 Diabetes (T2D) in adults justifies a call for the urgent of implementation of 'population-based interventions that prevent diabetes, enhance its early detection, and use lifestyle and pharmacological interventions to prevent or delay its progression to complications'.[1] In March 2015, NHS England and Public Health England (PHE) launched the National NHS Diabetes Prevention Programme (NDPP), which is a pragmatic lifestyle intervention that targets adults with intermediate hyperglycaemia (glucose levels associated with a high risk of developing T2D). The NDPP aims 'to significantly reduce the 4 million people in England otherwise expected to have Type 2 Diabetes (T2D) by 2025' based on evidence from 'well-designed randomised controlled trials (RCTs) in Finland, the USA, Japan, China and India'.[2] Many studies have used Markov chain models to estimate the impacts of such preventive interventions using transition probabilities between states: 'normoglycaemia' and 'intermediate hyperglycaemia' (glucose levels associated with a low and high risks of developing T2D), T2D and death. When we used these models,[3] we found, however, that our projections of the future prevalence of T2D in 2025 in England, in the absence of a preventive intervention, was much higher than 4 million, which is based on PHE's epidemiological model. Epidemiological models give future projections of the prevalence of T2D (at future time t, N(t)) by multiplying projections of the country's population by age and sex (at time t (**P**(t)) by projections of age-specific prevalence of diabetes (at time t, **D**(t)). (N(t) = **D**(t)* **P**(t)).) Hence this study, which is a critical review of methods of epidemiological and Markov chain models. Although we have used England for

For peer review only - http://bmjopen.bmj.com/site/about/guidelines.xhtml

the purpose of comparing projections by these different models, our study raises general questions about their validity. And hence of the evidence available to governments assessing the urgency of preventing T2D and choosing between different interventions. We consider only adults with diabetes. We use 'diabetes' to cover all types of diabetes, T2D for adults with type 2, 'true' prevalence for both diagnosed and undiagnosed diabetes and T2D.

Methods

Rapid reviews

Our comparisons of projections of different models builds on two reviews of the literature, which were designed to be rapid (not systematic): "a type of knowledge synthesis in which components of the systematic review process are simplified or omitted to produce information in a short period of time"[4]. We used stringent criteria to identify the principal methods of each type of models. These reviews were undertaken in March 2018, of articles published at any time available on Web of science and PubMed, which together provide a comprehensive coverage of the literature in the medical and applied health research fields. (The search strategy of each review is given in Table 1.1 of Appendix 1.) Articles included in each review were critically appraised and technical specifications of the models and projections were extracted and tabulated. The flowcharts in Figures 1 and 2 show the screening process.

Review 1 aimed to identify primary studies published from 2010 of models giving estimates of the prevalence of diabetes in adults in England or the UK. We examined how the models take account of future changes in age-specific prevalence rates and test their validity.

Review 2 aimed to identify primary studies using Markov chain models that reported results of interventions to prevent T2D. We included articles using Markov chain models to run economic analyses, utility analyses and cost effectiveness analyses of interventions targeting people diagnosed with T2D, or

For peer review only

with intermediate hyperglycaemia according to different measures: Glycated Haemoglobin (HbA1c), Impaired Fasting Glucose (IFG), and Impaired Glucose Tolerance (IGT) (Definitions are given in Table 1.2 of Appendix 1). We compared models' transition probabilities, estimates of the future prevalence of T2D without a preventive intervention, and tests of validation. Appendix 2 gives more details on the review of Markov chain models,

Figure 1 - Review flowchart of epidemiological models to go about here

Figure 2: Review flowcharts of Markov chain models to go about here

Markov chain model

From review 2 we derived matrices of transition probabilities to develop our own Markov chain models (see Figure 3), which are based on a cycle length of 1 year, to make projections of T2D cases in England without an intervention, up to 2035. The data sources of our estimates for England, of the prevalence of diabetes, intermediate hyperglycaemia and normoglycaemia; and of mortality rates of those with T2D, intermediate hyperglycaemia and normoglycaemia are given in Table 1.3 of Appendix 1.

.

Given doubts over the reliability of diagnosing intermediate hyperglycaemia (IH),[5] we examined the robustness of our results by using the PHE estimate (IH = 5.05 million), and the extreme value of zero (IH = 0). The hazard ratios, with reference to those with normoglycaemia, for those with intermediate hyperglycaemia found in a systematic review[6] defined by HbA1c and IGT were 0.97 and 1.32. We used 1.32 for IGT, but 1 for HbA1c because their estimate of 0,97 is not significantly different from 1.

For peer review only - http://bmjopen.bmj.com/site/about/guidelines.xhtml

Figure 3: Our Markov chain model to go about here

Estimating the trend in diagnosed diabetes

We estimated, by OLS regression analysis (using R),[7] the trend increase in numbers diagnosed with diabetes by general practitioners in England, as reported in the Quality Outcomes Framework (QOF) from 2004-05 (2004) to 2017-18 (2017)).[8] We used these estimates to give projections of the future prevalence of diagnosed diabetes to 2035.

Comparing projections of the prevalence of diabetes

We compared three sets of projections of the prevalence of diabetes and T2D in England from:

- different epidemiological models,
- the trend in QOF data,
- our Markov chain models.

The ratios we used for making comparisons across different estimates and the sources are given in Table 1.4 of Appendix 1.

Patients and public involvement

Patients and the public were not involved in this research study.

Results

Rapid review 1: Methods of epidemiological models

Rapid review 1 of methods of epidemiological models retrieved 633 articles and from their citations we identified a further five by snowballing[9]. After removing duplicates, we screened 597 articles, of which 11 were relevant and fully assessed. After reviewing the full articles, five were excluded and seven were included in our analysis[10–16]. This review identified four different underlying models described in Table 1 which have been used to give five

For peer review only - http://bmjopen.bmj.com/site/about/guidelines.xhtml

different projections of the future prevalence of diabetes for England and the UK. Two models produce global estimates: Shaw et al,[10] Guariguata et al[17], which is used by Whiting et al[11] and Guariguata et al;[12] and two for England only, the PHE model,[14] and the Association of Public Health Observatories (APHO) Diabetes Prevalence Model,[15] which is used by Hex et al[13] and Gatineau et al.[16]

For peer review only

For peer review only - http://bmjopen.bmj.com/site/about/guidelines.xhtml

Table 1: Methods of epidemiological models

Model	Method of estimation	Prevalence rates used for projections	Validation against QOF data?	Model validation?	Confidence intervals?
Shaw et al[10]	Logistic regression	Age & sex	No	No	No
Guariguata et al[17]	Logistic regression	Age & sex, & urban / rural	No	No	No
Association of Public Health Observatories (APHO)[15,16]	Direct estimation from HSE for age, sex, & IMD. Trend in obesity estimated by linear regression.	Age & sex, Index of Multiple Deprivation (2004), Ethnicity & increases in obesity	Yes for 2008/09	No	Yes
PHE[14]	Logistic regression	Age & sex, ethnicity, IMD 2015	Yes for 2014/15	Yes: refitting model on 70% of data & assessing against remaining 30%	No

Each epidemiological model uses: projected population changes; and estimates of the true age-specific prevalence rates of diabetes, from past annual Health Surveys for England (HSE), which are subject to two limitations. First, the small size of the sample means that the point estimate for the year of the survey is surrounded by large confidence interval estimates. Gatineau et al indicate that the HSE survey for 2013 gives point estimate of prevalence of 7.3% with confidence interval estimates ranging from 4.3 to 10.3%.[16] The PHE model[14] reduces the sampling error from HSE by using three years of data (2012, 2013 and 2014). Second, the HSE estimates of prevalence are based on those who self-reported a diabetes diagnosis made by a doctor (by HbA1c or FPG); and, for those who have not been diagnosed and agreed to have a blood test, having a HbA1c value of 6.5% or more.[14] Hence these estimates may be in error because of poor reliability of self-reporting or because of actual diagnostic errors. Barry et al (p. 9) report that ‘The most commonly used test (HbA1c) is neither sensitive nor specific; the fasting glucose test is specific but not

For peer review only - http://bmjopen.bmj.com/site/about/guidelines.xhtml

sensitive'. [5] Holman et al (p.6) pointed out, however, that 'Although HbA1c and fasting identify different groups of people with undiagnosed diabetes, the proportion of people that are identified is similar'.[15]

Our review aimed to answer two questions about the models.

1. *How were the models validated?* A basic test of the validity of a forecasting model is to apply this to past data to predict a known future: e.g. does the model using HSE data from 2004 predict prevalence as estimated from HSE data in 2014? None of the accounts of the models we reviewed reports such a test. The PHE model[14] was validated by refitting the model on 70% of the data (randomly selected) and checking its estimates against the remaining 30% of data.
2. *Did the models try to take account of future changes in age-specific prevalence rates?* Only the APHO model[15] aimed to do this by estimating the net effect of trends in: changes in ethnicity; and being overweight and obese to create a sex-specific obesity adjustment index. They did not, however, give details of how that index was modelled. The other three models[10,14,17] assumed that future age-specific prevalence of diabetes would be as estimated from past HSEs.

The epidemiological models we reviewed are focused on estimating geographical variations in the future prevalence of diabetes within countries, rather than giving sound estimates of future totals.

Rapid review 2: Markov chain models

Rapid review 2 of Markov chain models identified 304 articles. An additional one was snowballed. After removing duplicates, 222 articles were screened, 20 of them were considered relevant and fully assessed. Of these, one was excluded because we could not locate it, one did not report the results of a Markov chain model, and one modelled the progression from diabetes to its complications only. Table 2 gives details of the remaining 17 articles,[18–34]ordered in terms of their completeness of information on transition probabilities. (More details

For peer review only - http://bmjopen.bmj.com/site/about/guidelines.xhtml

are given in Appendix 2.) Two articles did not report the measure of intermediate hyperglycaemia used.[30,34] Twelve reported a model using one risk measure only: nine models used IGT,[19,20,22–25,28,29,33] two HbA1c[27,31] and one FPG only.[18] Neumann et al reported two models, using IFG and IGT;[19] and Roberts et al[26], three models using HbA1c, IGT and IFG. Hence, we reviewed 20 models.

For peer review only

For peer review only - http://bmjopen.bmj.com/site/about/guidelines.xhtml

Table 2: Transition probabilities reported in different models

Reference	Measure of intermediate hyperglycaemia	Country	Normoglycaemia to Intermediate hyperglycaemia	Intermediate hyperglycaemia to Normoglycaemia	Normoglycaemia to T2D	T2D to Normoglycaemia	Intermediate hyperglycaemia to T2D	T2D to Intermediate hyperglycaemia	Mortality rates
Johansson et al, 2009 **[18]	FPG	Sweden	**NR**	**NR**	**NR**	**NR**	**NR**	**NR**	**NR**
Herman et al, 2005[29]	IGT	USA	**NR**	**NR**	**NR**	**NR**	10.80%[35]	**NR**	**NR**
Palmer et at, 2004[28]	IGT	Australia, France, Germany, Switzerland, & UK	**NR**	**NR**	**NR**	**NR**	11%[36]	**NR**	Intermediate hyperglycaemia:1.37 (1.05 - 1.79) Undiagnosed T2D: 1.76 (1.17 – 2.66) Diagnosed T2D: 2.26 (1.78 – 2.87)
Zhuo et al, 2012[31]	HbA1c	USA	**NR**	**NR**	**NR**	**NR**	0.07% to 18.9%[37]	**NR**	**NR**
Chen et al, 2001[30]	**NR**	Taiwan	**NR**	**NR**	1.10%[38]	**NR**	**NR**	**NR**	**NR**
Zhou et al 2005[27]	HbA1c	USA	**NR**	**NR**	0%	0%	**NR**	0%	**NR**
Schaufler et al, 2010[32]	IGT or IFG	Germany	male, 2.23% female, 1.45%[39]	**NR**	male, 2.51% & female, 1.66%[39]	**NR**	male, 4.79% female, 4.23%[39]	**NR**	**NR**[40] **(Source given for higher mortality rates for T2D)**

Reference	Measure of intermediate hyperglycaemia	Country	Normoglycaemia to Intermediate hyperglycaemia	Intermediate hyperglycaemia to Normoglycaemia	Normoglycaemia to T2D	T2D to Normoglycaemia	Intermediate hyperglycaemia to T2D	T2D to Intermediate hyperglycaemia	Mortality rates
Gillies et al, 2008[20]	IGT	UK	< 65, 1.66%<br>> 65, 2.49%[41]	**NR**	**NR**	**NR**	1.96% based on 12 studies[36,41–51]	**NR**	Increased risk of death with diabetes (hazard ratio) 0.756 (SE = 0.087) [52]<br>1% increase in HbA1c (hazard ratio) 0.104 (SE =0.039 [53]
Ikeda et al, 2010[33]	IGT	Japan	3.10% [54]	**NR**	0%	0%	**NR**	0%	Intermediate hyperglycaemia: 1.35<br>T2DM: 3.03 [55]
Smith et al, 2010[34]	NR	USA	4% [56]	**NR**	0.40% [57]	0%	10.80%[29]	0%	Intermediate hyperglycaemia: 1.7 [58]<br>stable T2D: 2 [59]<br>complicated T2D: 2.4 [60]
Neumann et al, 2017[19]	IGT	Sweden	Risk equation reported	Risk equation reported	0%	0%	Risk equation reported	Risk equation reported	No increased risk for intermediate hyperglycaemia.<br>T2D mortality not reported.
Caro et al, 2004[22]	IGT	Canada	16.30% (original estimate)	16.20% (original estimate)	0%	0%	6.30% (original estimate)	0%	Intermediate hyperglycaemia: 1.45 (original estimate)

For peer review only - http://bmjopen.bmj.com/site/about/guidelines.xhtml

Reference	Measure of intermediate hyperglycaemia	Country	Normoglycaemia to Intermediate hyperglycaemia	Intermediate hyperglycaemia to Normoglycaemia	Normoglycaemia to T2D	T2D to Normoglycaemia	Intermediate hyperglycaemia to T2D	T2D to Intermediate hyperglycaemia	Mortality rates
Neumann et al, 2011[23]	IGT	Germany	16.30% [22]	16.20% [22]	0%	0%	6.00%[61]	0.50% (original estimate)	**NR**
Liu et al, 2013[24]	IGT	China	1.28% [62]	11.60% [63]	0%	0%	initiation ages<br>• 25: 0.644%<br>• 40: 16.7%<br>• 60: 57.8%<br>[64–66]	0%	**NR**
Wong et al, 2016[25]	IGT	Hong Kong	16.30% [22]	16.20% [22]	0%	0%	years 1-3 11% [36]<br>years > 4 5.6% [67]	0%	Intermediate hyperglycaemia: 1.50 (1.10–2.00)<br>T2DM: 2.30 (1.60–3.20) [21]
Roberts et al, 2018[26]	IGT	England	6.33% [41]	8.97% [68]	0%	0%	4.55%[69]	0%	Intermediate hyperglycaemia: 1.50<br>T2D: 1.9 [70]
	HbA1c	England	6.86% [41]	8.97% [68]	0%	0%	3.55%[69]	0%	Intermediate hyperglycaemia: 1.2<br>T2D: 1.6 [70]
	IFG (ADA)	England	6.86% [41]	8.97% [68]	0%	0%	4.74%[69]	0%	Intermediate hyperglycaemia: 1.2<br>T2D: 1.6<br>[70]

For peer review only - http://bmjopen.bmj.com/site/about/guidelines.xhtml

Reference	Measure of intermediate hyperglycaemia	Country	Normoglycaemia to Intermediate hyperglycaemia	Intermediate hyperglycaemia to Normoglycaemia	Normoglycaemia to T2D	T2D to Normoglycaemia	Intermediate hyperglycaemia to T2D	T2D to Intermediate hyperglycaemia	Mortality rates
Range	IGT		1.28 -16.30%	8.97-16.20%	0.00-4.6%	0%	1.96-11.00%	0.00-0.5%	Intermediate hyperglycaemia:1.35-1.7<br>T2D: 1.76-3.03
Meta-analyses	IGT						4.55%[69]		Intermediate hyperglycaemia: 1.32 (1.23 to 1.40) [6]
	HbA1c						3.55%[69]		Intermediate hyperglycaemia: 0.97 (0.88 to 1.07) [6]
	IFG (ADA)						3.54%[69]		Intermediate hyperglycaemia: 1.13, (1.02 - 1.25) [6]

Key and Notes:

NR: not reported, 0%: not allowed

*Relative risk over normoglycaemia. Ranges in parentheses are 95% confidence intervals

** The model and data sources were described in a technical report

For peer review only - http://bmjopen.bmj.com/site/about/guidelines.xhtml

These Markov chain models use two different sources of data to estimate transition probabilities: between states other than death (ideally from RCTs or meta analyses); and from these states to death (based on mortality rates of a country). As these models require transition probabilities from each state to sum to one, the validity of the interaction between two sets of transition probabilities needs to be tested. We have done this by comparing our models' estimates of the number of T2D cases in the absence of any preventive intervention with those from epidemiological models.

Our review aimed to answer two questions about the models:

1. *How do transition probabilities compare?* Table 2 shows that of the 17 articles only five reported the full set of transition probabilities between states other than death (i.e. normoglycaemia, intermediate hyperglycaemia, and T2D). All models, except that of Neumann et al,[23] allow transitions from T2D to death only. Neumann et al[23] allow transition (at a low probability, 0.5%) from T2D to intermediate hyperglycaemia (IGT) (because 'this transition exists but seldom occurs', p 4). Only two models allow transition from normoglycaemia directly to T2D: Schaufler et al[32] (IFG or IGT - for males, 2.51% and females, 1.66%) and Smith et al (measure of intermediate hyperglycaemia not specified, 0.40%).[34] Table 2 shows that wide ranges of transition probabilities used by the different IGT models: from normoglycaemia to intermediate hyperglycaemia, 1.28 to 16.30%; from intermediate hyperglycaemia to low, 8.97-16.20%; normoglycaemia to T2D, 0.00-4.6%; intermediate hyperglycaemia to T2D, 1.96-11.00%. A meta-analysis recommended a rate of 4.55% for the last.[69]

   No article reports the transition probabilities from different states to death (i.e. mortality rates for each state). Six articles report the relative risk of mortality for intermediate hyperglycaemia and T2D compared with normoglycaemia. For IGT these ranged for intermediate hyperglycaemia (IGT) from 1.35 to 1.7, and for T2D from 1.76 to 3.03. Roberts et al[26] report this for HbA1c to be 1.2. A systematic review and meta-analysis[6] derived estimates (with 95% confidence intervals) to

For peer review only - http://bmjopen.bmj.com/site/about/guidelines.xhtml

be: for IGT 1.32 (1.23 to 1.40) and for HbA1c 0.97 (0.88 to 1.07). One article[23] reported a matrix in which probabilities of transitions between states other than death sum to one, which implies no one dies.

2. *How were models validated?* Of the 17 articles, estimating the impacts of preventive interventions on prevalence of T2D, only four[16,22,31,71] modelled the general population (with normoglycaemia and intermediate hyperglycaemia); and, of these, only Caro et al [22] reported estimates of those developing T2D in the absence of a preventive intervention: 9.6% of 55-year-old men and women over three years. They did not report a check of their estimate against other projections. Of the other articles, which modelled populations with intermediate hyperglycaemia only, only three reported estimates of the percentages developing T2D in the absence of intervention [13,21,22]. Only two reported tests of validation: against the observed incidence in RCTs. Palmer et al [21] validated the results of their model against the observed incidence in the US DPP and follow-up DPPOS trials (correlation coefficient of 0.9987). Roberts et al 2018[26] validated their results against the National Diabetes Audit 2015-2016 [72] adjusted for undiagnosed T2D and reported the prevalence of T2D by age groups. They estimated the percentages developing T2D over 10 years to be: for those with IGT to be 23%; and, for both IFG and HbA1c, 19%.

The primary focus of the articles we reviewed is on estimating the ratio of costs to benefits of preventive interventions for those with IGT. None reported another ratio that governments need to know: of the numbers of T2D cases prevented to projections of its future prevalence in the general population.

Our Markov chain models

Our Markov chain models are designed to use available data for England with one transition probability only between states. As PHE identify those with intermediate hyperglycaemia using HbA1c (from 5.7% to 6.4%),[14] the model used by Roberts et al[26] for HbA1c is most appropriate for projecting the prevalence of T2D in England. They used the recommended transition

For peer review only - http://bmjopen.bmj.com/site/about/guidelines.xhtml

probabilities from different risk measures of intermediate hyperglycaemia to T2D identified by a meta-analysis.[69] Neumann et al[23] and Caro et al[22] have similar transition probabilities, which are higher than those of Roberts et al, [26] for IGT from normoglycaemia to intermediate hyperglycaemia, and intermediate hyperglycaemia to T2D: 16.3% and 6.00% compared with 6.33% and 4.55%. We used the transition probabilities used by Neumann et al[23] because that is more recent. Model 1 is based on Roberts et al (HbA1c).[26] Model 2 is Model 1 modified to give the projections of PHE. Model 2's transition probability from intermediate hyperglycaemia to T2D (0.013) is a third of that of Model 1 (0.036) and below the lowest rate of any model we reviewed (0.02). (Model 2 has a corresponding increase in the transition probability of remaining in intermediate hyperglycaemia (0.836 to 0.878).) Model 3 is based on Neumann et al.[23] Details of the models are given in Table 1.5 of Appendix 1.

Estimating the trend in diagnosed diabetes

Table 3 reports the OLS estimate of the trend in diagnosed diabetes from QOF data, which gives an annual rate of increase of 11%.

Table 3: The trend model from QOF data

Coefficients	Value	Standard error	T	Pr > |t|	Lower bound (95%)	Upper bound (95%)
Intercept	-229	2.436	94.228	< 0.0001	-234.889	-224.167
Year	0.115	0.001	95.235	< 0.0001	0.113	0.118
Adjusted R-squared	0.9987					

Comparing projections of the future prevalence of T2D

Table 4 gives: for the different epidemiological models their defined populations, data sources, and projections of diabetes true prevalence (in millions); and comparable estimates of the true prevalence of diabetes from the QOF trend

For peer review only - http://bmjopen.bmj.com/site/about/guidelines.xhtml

(increased by a third). It also gives the annual rate of increase in prevalence from the first in the series to the last. Table 4 shows that, for the three models that do not allow for increase in prevalence rates by age and sex,[10–12] the older the HSE data used, the lower is the estimate of the rate of increase in prevalence for England.

For peer review only

For peer review only - http://bmjopen.bmj.com/site/about/guidelines.xhtml

Table 4: True diabetes prevalence (millions) estimated by different epidemiological models and from the QOF Trend

Source of estimate	Population	Data source	Details of series									
			First year	Prevalence	Final Year	Prevalence	Annual rate of increase (%)*	Projections				
								2015	2020	2025	2030	2035
Shaw et al[10]	UK: 20 to 79 (UN, 2007)	HSE (2003)	2010	2.14	2030	2.55	2.0				2.55	
Whiting et al[11]	UK: 20 to 79 (UN, 2011)	HSE (2004 & 2009)	2011	3.06	2030	3.65	3.1				3.65	
Guariguata et al[12]	UK: 20 to 79 (UN, 2011)	HSE (2004)	2013	2.98	2035	3.62	2.9					3.62
Holman et al[15]	England: >15 (ONS)	HSE (2006)	2010	3.10	2030	4.60 (3.25-6.88)	7.5	3.47 (2.47-5.07)	3.82 (2.70-5.62)	4.19 (2.93-6.19)	4.60 (3.25-6.88)	
PHE[14]	England: >15 (ONS)	HSE (2012, 2013 & 2014)	2015	3.81	2035	4.94	5.6	3.81	4.09	4.39	4.68	4.94
QOF trend**	England: >15 registered with GPs	QOF (2004-05 to 2017-18)	2004-05	1.77	2017	3.20	11.0	3.99 (3.88-4.09)	4.72 (4.61-4.84)	5.46 (5.32-5.59)	6.19 (6.04-6.35)	6.93 (6.75-7.11)

Notes:

*Estimated as the rate of increase from the first estimate to the last

** To estimate the true prevalence from the QOF trend these estimates were increased by a third.

For peer review only

For peer review only - http://bmjopen.bmj.com/site/about/guidelines.xhtml

Global models are used to give three projections (in millions) for diabetes prevalence in the UK (aged 20 to 79): for 2030 (2.55[10] and 3.65[11]) and 2035 (3.62).[12] Each projection is below the PHE[73] model's estimate for England for 2015 (3.81) (based on HSEs for 2012, 2103 and 2014). There are two reasons for this: their low rates of increase over time; and excision of those over 79, who we estimated to account for nearly 30% who would be over 15 in England and develop diabetes (see Table 1.4 of Appendix 1). The projections by these global models are not examined further.

Two models give projections for England (aged over 15): the PHE model[73] gives projections for 2030 (4.68) and 2035 (4.94); and APHO only up to 2030 (4.60) (with 95% confidence intervals from 3.25 to 6.88).[15] Although the two accounts of the APHO model report the same projection for 2030; one estimated the prevalence of diabetes in 2010 (3.10)[15] to be higher than the other for 2013 (2.17).[16] Also, one attributed approximately half of the increase in prevalence to 2030 to increases in obesity,[15] the other estimated this to have been a third.[16]

Figure 4 compares the projections of the true prevalence of diabetes: by PHE, and (with 95% confidence intervals) by Holman et al[15], and from the QOF trend (for the last two we show their 95% confidence interval estimates). The estimates from the QOF trend are the highest and towards the upper end of the 95% confidence intervals of Holman et al.[15] For 2025, projections (with 95% confidence interval estimates where available) are as follows: by Holman et al, 4.19 (2.93-6.19); by PHE,[73] 4.39; from the QOF trend, 5.46 (5.32-5.59).

Figure 4: Projections of true diabetes prevalence by PHE, Holman et al & from the QOF trend (millions): 2005 to 2035 to go about here

Figure 5 compares projections of the true prevalence of T2D in England to 2035 from PHE, the QOF trend, and the Markov models. Table 5 gives projections in

For peer review only - http://bmjopen.bmj.com/site/about/guidelines.xhtml

millions for 2025. These show that the projections: by Model 2 replicated the projections by PHE; by Model 1 are above those from the QOF trend; by Model 3 seem to be implausibly explosive. Figure 5 and Table 5 also show that projections by models 1 and 3 are robust to errors in the estimate of the numbers of those with intermediate hyperglycaemia in 2015.

Table 5: Projections of adults with T2D in England for 2025

Model	Projections for 2025 (millions)			
	Statistical		Markov (numbers with intermediate hyperglycaemia in 2015)	
	Point estimate	95% confidence intervals	5.05*	Zero
PHE	3.95	n.a.		
QOF trend;	4.91	4.79 to 5.03		
Model 1**			5.64	5.05
Model 2 ***			3.86	
Model 3****			9.10	8.60

Notes

n.a Not available

* as estimated by PHE

**based on Roberts et al,[26]

***based on Roberts et al,[26] but modified to reproduce the QOF trend to 2035

**** based on Neuman et al[23]

Figure 5: Projections of adults with T2D in England to go about here

Discussion

The four epidemiological models we reviewed[10,14,15,17] use past estimated prevalence rates by age and sex and projected changes in populations. They are focused on estimating geographical variations in the future prevalence of diabetes within countries, rather than giving sound estimates of future totals. Only one model aims to take account of increases in prevalence rates.[15] No model was validated by using past data to predict a known future.

Of the five projections of diabetes prevalence, for England and the UK we reviewed,[10–12,14,15] only one[15] reported confidence intervals. Three

For peer review only - http://bmjopen.bmj.com/site/about/guidelines.xhtml

projections of diabetes prevalence for the UK (aged 20 to 79) by global models for 2030[10,11] and 2035[12] are below the PHE estimate for 2015 for England (over 15). This raises questions over the validity of their global projections and their excision of those over 79 (estimated to account for nearly 30% of developing T2D after 2030). The estimates of T2D prevalence (in millions) in England for 2025 (with 95% confidence intervals where available) were: by PHE[73] 3.95; by the APHO model[15,16] 3.77 (2.64 to 5.57); from the QOF trend, 4.91 (4.79 to 5.03).

Markov chain models of the impacts of interventions that aim to prevent T2D require estimates of transition probabilities between states other than death and from these states to death, which are based on different sources. None of the articles we reviewed reported the complete matrix of transition probabilities. Only two[21,26] reported checks on the validity of their models using their projections of numbers developing T2D with no intervention, and none against projections from epidemiological models. This disconnect means that governments lack information on what the impact on the future prevalence of T2D might be if, like England they were to roll out at scale interventions like the NDPP. Two of our own Markov chain models (based on those of Roberts et al (for HbA1c),[26] and Neuman et al (for IGT)[23] gave projections (in millions) with T2D for England (for 2025 of 5.64 and 9.1 million), which are above all estimates from the epidemiological models we reviewed. Our model that reproduced PHE's projections has a lower rate of transition from intermediate hyperglycaemia to T2D than any of the models we reviewed.

The limitations of our research are that we did not undertake systematic reviews, hence we may have omitted relevant articles. We also developed simple transparent Markov chain models and a simple regression model to project a trend using QOF data.

Conclusions

The models we reviewed have desirable attributes for informing policy on preventing T2D by being simple and transparent and designed to use routinely-

For peer review only - http://bmjopen.bmj.com/site/about/guidelines.xhtml

available data. The Markov Chain models, for example, do not take account of diabetic complications or age. We have considered whether both types of models are requisite in their form and content [74] for the objective of giving reliable estimates of the order of magnitude of the future prevalence of T2D. We conclude that they are not. This is because both classes of model we reviewed often lack of any tests of validity, and the differences in projections of the future prevalence of T2D differ by orders of magnitude.

There are three implications of our study. First, methods of current epidemiological models are designed to underestimate the scale of increases in the future prevalence of T2D, and hence the urgency for governments of implementing preventive interventions. Second, models used to assess the preventive interventions lack transparency and tests of validity. Third, we need research to remedy these deficiencies.

Figure legends

Figure 1 - Review flowchart of epidemiological models

Figure 2: Review flowcharts of Markov chain models

Figure 3: Our Markov chain model

Figure 4: Projections of true diabetes prevalence by PHE, Holman et al & from the QOF trend (millions): 2005 to 2035

Figure 5: Projections of adults with T2D in England

Author contributions

Mi Jun Keng did the original work in developing initial Markov Chain models to estimate the impacts of preventive interventions on the future prevalence of Type 2 Diabetes (T2D) in England and has been involved throughout this project. Chiara De Poli worked with Mi Jun in developing those models, undertook the rapid reviews of epidemiological and Markov Chain models, and commented on

For peer review only - http://bmjopen.bmj.com/site/about/guidelines.xhtml

For peer review only

drafts of this paper. Gwyn Bevan prepared drafts of the paper, reviewed epidemiological and Markov Chain models, developed the models used in this paper, and undertook comparisons of projections. Rosalind Raine took part in the workshops on the models, reviewed our methods and findings, and commented on drafts.

Data statement

The data we have used are from cited public sources and we give details for our Markov Chain models in Table 1.2 of Appendix 1.

.

For peer review only

For peer review only - http://bmjopen.bmj.com/site/about/guidelines.xhtml

Declaration of interests

There are no conflicts of interest.

Funding

Funding was from the National Institute for Health Research (NIHR) Collaboration for Leadership in Applied Health Research and Care North Thames at Barts Health NHS Trust, which had no role in the writing of the manuscript or the decision to submit it for publication. None of us has been paid to write this article. The corresponding author has had full access to all the data in the study and had final responsibility for the decision to submit for publication.

Ethics approval

No ethics approval was sought for this study.

References

1 NCD Risk Factor Collaboration (NCD-RisC). Worldwide trends in diabetes since 1980: a pooled analysis of 751 population-based studies with 4.4 million participants. *Lancet (London, England)* 2016;**387**:1513–30. doi:10.1016/S0140-6736(16)00618-8

2 Public Health England, NHS England. National NHS Diabetes initiative launched in major bid to prevent illness (press release). 2015.

3 De Poli C, Bevan G, Keng MJ, *et al.* Impact of interventions to prevent diabetes in England: a simulation model. *Lancet* 2017;**390**:S36. doi:10.1016/S0140-6736(17)32971-9

4 Khangura S, Konnyu K, Cushman R, *et al.* Evidence summaries: The evolution of a

For peer review only - http://bmjopen.bmj.com/site/about/guidelines.xhtml

rapid review approach. *Syst Rev* Published Online First: 2012. doi:10.1186/2046-4053-1-10

5 Barry E, Roberts S, Oke J, *et al.* Efficacy and effectiveness of screen and treat policies in prevention of type 2 diabetes: systematic review and meta-analysis of screening tests and interventions. *BMJ* 2017;**356356**. doi:10.1136/bmj.i6538

6 Huang Y, Cai X, Mai W, *et al.* Association between prediabetes and risk of cardiovascular disease and all cause mortality: systematic review and meta-analysis. *BMJ* 2016;**355**:i5953. doi:10.1136/bmj.i5953

7 RStudio Team. RStudio: Integrated Development for R. 2015.

8 NHS Digital. Quality and Outcomes Framework (QOF) - 2016-17. 2017.

9 Wohlin C. Guidelines for snowballing in systematic literature studies and a replication in software engineering. In: *ACM International Conference Proceeding Series*. 2014. doi:10.1145/2601248.2601268

10 Shaw JEE, Sicree RAA, Zimmet PZZ. Global estimates of the prevalence of diabetes for 2010 and 2030. *Diabetes Res Clin Pract* 2010;**87**:4–14. doi:10.1016/j.diabres.2009.10.007

11 Whiting DR, Guariguata L, Weil C, *et al.* IDF diabetes atlas: global estimates of the prevalence of diabetes for 2011 and 2030. *Diabetes Res Clin Pract* 2011;**94**:311–21. doi:10.1016/j.diabres.2011.10.029

12 Guariguata L, Whiting DR, Hambleton I, *et al.* Global estimates of diabetes prevalence for 2013 and projections for 2035. *Diabetes Res Clin Pract* 2014;**103**:137–49. doi:10.1016/j.diabres.2013.11.002

13 Hex N, Bartlett C, Wright D, *et al.* Estimating the current and future costs of Type 1 and Type 2 diabetes in the UK, including direct health costs and indirect societal and productivity costs. *Diabet Med* 2012;**29**:855–62. doi:10.1111/j.1464-5491.2012.03698.x

14 Public Health England. Technical document for the diabetes prevalence model for England 2016. London: 2016.

15 Holman N, Forouhi NG, Goyder E, *et al.* The Association of Public Health

For peer review only - http://bmjopen.bmj.com/site/about/guidelines.xhtml

Observatories (APHO) Diabetes Prevalence Model: estimates of total diabetes prevalence for England, 2010-2030. *Diabet Med* 2011;**28**:575–82. doi:10.1111/j.1464-5491.2010.03216.x

16 Gatineau M, Hancock C, Holman N, *et al.* Adult obesity and type 2 diabetes. London, UK: 2014.

17 Guariguata L, Whiting D, Weil C, *et al.* The International Diabetes Federation diabetes atlas methodology for estimating global and national prevalence of diabetes in adults. *Diabetes Res Clin Pract* 2011;**94**:322–32. doi:10.1016/j.diabres.2011.10.040

18 Johansson P, Östenson C-G, Hilding AM, *et al.* A cost-effectiveness analysis of a community-based diabetes prevention program in Sweden. *Int J Technol Assess Health Care* 2009;**25**:350–8. doi:10.1017/S0266462309990079

19 Neumann A, Lindholm L, Norberg M, *et al.* The cost-effectiveness of interventions targeting lifestyle change for the prevention of diabetes in a Swedish primary care and community based prevention program. *Eur J Heal Econ* 2017;**18**:905–19. doi:10.1007/s10198-016-0851-9

20 Gillies CL, Lambert PC, Abrams KR, *et al.* Different strategies for screening and prevention of type 2 diabetes in adults: cost effectiveness analysis. *BMJ* 2008;**336**.

21 Palmer AJ, Tucker DMD, Polkinghorne KR, *et al.* Cost and clinical implications of diabetes prevention in an Australian setting: A long-term modeling analysis. *Prim Care Diabetes* 2012;**6**:109–21. doi:10.1016/j.pcd.2011.10.006

22 Caro JJ, Getsios D, Caro I, *et al.* Economic evaluation of therapeutic interventions to prevent Type 2 diabetes in Canada. *Diabet Med* 2004;**21**:1229–36. doi:10.1111/j.1464-5491.2004.01330.x

23 Neumann A, Schwarz P, Lindholm L. Estimating the cost-effectiveness of lifestyle intervention programmes to prevent diabetes based on an example from Germany: Markov modelling. *Cost Eff Resour Alloc* 2011;**9**:17. doi:10.1186/1478-7547-9-17

24 Liu X, Li C, Gong H, *et al.* An economic evaluation for prevention of diabetes mellitus in a developing country: a modelling study. *BMC Public Health*

For peer review only

2013;**13**:729. doi:10.1186/1471-2458-13-729

25 Wong CKH, Jiao F-F, Siu S-C, *et al.* Cost-Effectiveness of a Short Message Service Intervention to Prevent Type 2 Diabetes from Impaired Glucose Tolerance. *J Diabetes Res* 2016;**2016**:1–8. doi:10.1155/2016/1219581

26 Roberts S, Craig D, Adler A, *et al.* Economic evaluation of type 2 diabetes prevention programmes: Markov model of low- and high-intensity lifestyle programmes and metformin in participants with different categories of intermediate hyperglycaemia. *BMC Med* 2018;**16**:16. doi:10.1186/s12916-017-0984-4

27 Zhou H, Isaman DJM, Messinger S, *et al.* A computer simulation model of diabetes progression, quality of life, and cost. *Diabetes Care* 2005;**28**:2856–63.

28 Palmer AJ, Roze S, Valentine WJ, *et al.* Intensive lifestyle changes or metformin in patients with impaired glucose tolerance: Modeling the long-term health economic implications of the diabetes prevention program in Australia, France, Germany, Switzerland, and the United Kingdom. *Clin Ther* 2004;**26**:304–21. doi:10.1016/S0149-2918(04)90029-X

29 Herman WH, Hoerger TJ, Brandle M, *et al.* The cost-effectiveness of lifestyle modification or metformin in preventing type 2 diabetes in adults with impaired glucose tolerance. *Ann Intern Med* 2005;**142**:323–32.

30 Chen TH-H, Yen M-F, Tung T-H. A computer simulation model for cost–effectiveness analysis of mass screening for Type 2 diabetes mellitus. *Diabetes Res Clin Pract* 2001;**54**:37–42. doi:10.1016/S0168-8227(01)00307-2

31 Zhuo X, Zhang P, Selvin E, *et al.* Alternative HbA1c Cutoffs to Identify High-Risk Adults for Diabetes Prevention. *Am J Prev Med* 2012;**42**:374–81. doi:10.1016/j.amepre.2012.01.003

32 Schaufler TM, Wolff M. Cost Effectiveness of Preventive Screening Programmes for Type 2 Diabetes Mellitus in Germany. *Appl Health Econ Health Policy* 2010;**8**:191–202. doi:10.2165/11532880-000000000-00000

33 Ikeda S, Kobayashi M, Tajima N. Cost-effectiveness analysis of voglibose for prevention of type 2 diabetes mellitus in Japanese patients with impaired glucose

For peer review only - http://bmjopen.bmj.com/site/about/guidelines.xhtml

tolerance. 2010;**1**:252–8. doi:10.1111/j.2040-1124.2010.00052.x

34 Smith KJ, Hsu HE, Roberts MS, *et al.* Cost-effectiveness analysis of efforts to reduce risk of type 2 diabetes and cardiovascular disease in southwestern Pennsylvania, 2005-2007. *Prev Chronic Dis* 2010;**7**:A109.

35 The CDC Diabetes Cost-effectiveness Group TCDC. Cost-effectiveness of Intensive Glycemic Control, Intensified Hypertension Control, and Serum Cholesterol Level Reduction for Type 2 Diabetes. *JAMA* 2002;**287**:2542. doi:10.1001/jama.287.19.2542

36 Knowler WC, Barrett-Connor E, Fowler SE, *et al.* Reduction in the incidence of type 2 diabetes with lifestyle intervention or metformin. *N Engl J Med* 2002;**346**:393–403. doi:10.1056/NEJMoa012512

37 Selvin E, Steffes MW, Zhu H, *et al.* Glycated Hemoglobin, Diabetes, and Cardiovascular Risk in Nondiabetic Adults. *N Engl J Med* 2010;**362**:800–11. doi:10.1056/NEJMoa0908359

38 Kuo HS, Chang HJ, Chou P, *et al.* A Markov chain model to assess the efficacy of screening for non-insulin dependent diabetes mellitus (NIDDM). *Int J Epidemiol* 1999;**28**:233–40.

39 Icks A, Rathmann W, Haastert B, *et al.* Cost-Effectiveness of Type 2 Diabetes Screening: Results from Recently Published Studies. *Das Gesundheitswes* 2005;**67**:167–71. doi:10.1055/s-2005-858232

40 Roper NA, Bilous RW, Kelly WF, *et al.* Cause-specific mortality in a population with diabetes: South Tees Diabetes Mortality Study. *Diabetes Care* 2002;**25**:43–8.

41 Meigs JB, Muller DC, Nathan DM, *et al.* The natural history of progression from normal glucose tolerance to type 2 diabetes in the Baltimore Longitudinal Study of Aging. *Diabetes* 2003;**52**:1475–84.

42 Rasmussen SS, Glümer C, Sandbaek A, *et al.* Progression from impaired fasting glucose and impaired glucose tolerance to diabetes in a high-risk screening programme in general practice: the ADDITION Study, Denmark. *Diabetologia* 2007;**50**:293–7. doi:10.1007/s00125-006-0530-y

For peer review only

43 Wein P, Beischer N, Harris C, *et al.* A trial of simple versus intensified dietary modification for prevention of progression to diabetes mellitus in women with impaired glucose tolerance. *Aust N Z J Obstet Gynaecol* 1999;**39**:162–6.

44 Bonora E, Kiechl S, Willeit J, *et al.* Population-based incidence rates and risk factors for type 2 diabetes in white individuals: the Bruneck study. *Diabetes* 2004;**53**:1782–9.

45 Chiasson J-L, Josse RG, Gomis R, *et al.* Acarbose for prevention of type 2 diabetes mellitus: the STOP-NIDDM randomised trial. *Lancet* 2002;**359**:2072–7. doi:10.1016/S0140-6736(02)08905-5

46 de Vegt F, Dekker JM, Jager A, *et al.* Relation of impaired fasting and postload glucose with incident type 2 diabetes in a Dutch population: The Hoorn Study. *JAMA* 2001;**285**:2109–13.

47 Edelstein SL, Knowler WC, Bain RP, *et al.* Predictors of progression from impaired glucose tolerance to NIDDM: an analysis of six prospective studies. *Diabetes* 1997;**46**:701–10.

48 Eriksson J, Lindström J, Valle T, *et al.* Prevention of Type II diabetes in subjects with impaired glucose tolerance: the Diabetes Prevention Study (DPS) in Finland. Study design and 1-year interim report on the feasibility of the lifestyle intervention programme. *Diabetologia* 1999;**42**:793–801.

49 Eriksson J, Lehtovirta M, Ehrnstrom B, *et al.* Long-term beneficial effects of glipizide treatment on glucose tolerance in subjects with impaired glucose tolerance. *J Intern Med* 2006;**259**:553–60. doi:10.1111/j.1365-2796.2006.01633.x

50 Jarrett RJ, Keen H, Fuller JH, *et al.* Worsening to diabetes in men with impaired glucose tolerance ('borderline diabetes'). *Diabetologia* 1979;**16**:25–30.

51 Wareham NJ, Byrne CD, Williams R, *et al.* Fasting proinsulin concentrations predict the development of type 2 diabetes. *Diabetes Care* 1999;**22**:262–70.

52 DECODE study group. Glucose tolerance and mortality: comparison of WHO and American Diabetic Association diagnostic criteria. *Lancet* 1999;**354**:617–21. doi:10.1016/S0140-6736(98)12131-1

For peer review only - http://bmjopen.bmj.com/site/about/guidelines.xhtml

53 Rossing P, Hougaard P, Borch-Johnsen K, *et al.* Predictors of mortality in insulin dependent diabetes: 10 year observational follow up study. *BMJ* 1996;**313**:779–84.

54 Oizumi T. [Epidemiologic investigation on the incidence of diabetes mellitus from impaired glucose tolerance in population based study]. *Nihon Rinsho* 2005;**63 Suppl 2**:73–7.

55 Nakagami T, DECODA Study Group. Hyperglycaemia and mortality from all causes and from cardiovascular disease in five populations of Asian origin. *Diabetologia* 2004;**47**:385–94. doi:10.1007/s00125-004-1334-6

56 Orchard TJ, Temprosa M, Goldberg R, *et al.* The effect of metformin and intensive lifestyle intervention on the metabolic syndrome: the Diabetes Prevention Program randomized trial. *Ann Intern Med* 2005;**142**:611–9.

57 Fox CS, Pencina MJ, Meigs JB, *et al.* Trends in the Incidence of Type 2 Diabetes Mellitus From the 1970s to the 1990s: The Framingham Heart Study. *Circulation* 2006;**113**:2914–8. doi:10.1161/CIRCULATIONAHA.106.613828

58 Lakka H-M, Laaksonen DE, Lakka TA, *et al.* The metabolic syndrome and total and cardiovascular disease mortality in middle-aged men. *JAMA* 2002;**288**:2709–16.

59 Moss SE, Klein R, Klein BE. Cause-specific mortality in a population-based study of diabetes. *Am J Public Health* 1991;**81**:1158–62.

60 Fuller JH, Stevens LK, Wang SL. Risk factors for cardiovascular mortality and morbidity: the WHO Mutinational Study of Vascular Disease in Diabetes. *Diabetologia* 2001;**44 Suppl 2**:S54-64.

61 Tuomilehto J, Lindström J, Eriksson JG, *et al.* Prevention of Type 2 Diabetes Mellitus by Changes in Lifestyle among Subjects with Impaired Glucose Tolerance. *N Engl J Med* 2001;**344**:1343–50. doi:10.1056/NEJM200105033441801

62 Pan XR, Hu YH, Li GW, *et al.* Impaired glucose tolerance and its relationship to ECG-indicated coronary heart disease and risk factors among Chinese. Da Qing IGT and diabetes study. *Diabetes Care* 1993;**16**:150–6.

63 Harris MI, Klein R, Welborn TA, *et al.* Onset of NIDDM occurs at least 4-7 yr before

For peer review only - http://bmjopen.bmj.com/site/about/guidelines.xhtml

clinical diagnosis. *Diabetes Care* 1992;**15**:815–9.

64 Pan X-R, Li G-W, Hu Y-H, *et al.* Effects of Diet and Exercise in Preventing NIDDM in People With Impaired Glucose Tolerance: The Da Qing IGT and Diabetes Study. *Diabetes Care* 1997;**20**.

65 Jia WP, Pang C, Chen L, *et al.* Epidemiological characteristics of diabetes mellitus and impaired glucose regulation in a Chinese adult population: the Shanghai Diabetes Studies, a cross-sectional 3-year follow-up study in Shanghai urban communities. *Diabetologia* 2007;**50**:286–92. doi:10.1007/s00125-006-0503-1

66 Dunstan DW, Zimmet PZ, Welborn TA, *et al.* The rising prevalence of diabetes and impaired glucose tolerance: the Australian Diabetes, Obesity and Lifestyle Study. *Diabetes Care* 2002;**25**:829–34.

67 Diabetes Prevention Program Research Group, Knowler WC, Fowler SE, *et al.* 10-year follow-up of diabetes incidence and weight loss in the Diabetes Prevention Program Outcomes Study. *Lancet (London, England)* 2009;**374**:1677–86. doi:10.1016/S0140-6736(09)61457-4

68 Balk EM, Earley A, Raman G, *et al.* Combined Diet and Physical Activity Promotion Programs to Prevent Type 2 Diabetes Among Persons at Increased Risk: A Systematic Review for the Community Preventive Services Task Force. *Ann Intern Med* 2015;**163**:437–51. doi:10.7326/M15-0452

69 Morris DH, Khunti K, Achana F, *et al.* Progression rates from HbA1c 6.0–6.4% and other prediabetes definitions to type 2 diabetes: a meta-analysis. *Diabetologia* 2013;**56**:1489–93. doi:10.1007/s00125-013-2902-4

70 DECODE Study Group - European Diabetes Epidemiology. Is the current definition for diabetes relevant to mortality risk from all causes and cardiovascular and noncardiovascular diseases? *Diabetes Care* 2003;**26**:688–96.

71 Moody A, Cowley G, Fat LN, *et al.* Social inequalities in prevalence of diagnosed and undiagnosed diabetes and impaired glucose regulation in participants in the Health Surveys for England series. *BMJ Open* 2016;**6**:e010155. doi:10.1136/BMJOPEN-2015-010155

72 Healthcare Quality Improvement Partnership (HQIP). National Diabetes Audit,

For peer review only - http://bmjopen.bmj.com/site/about/guidelines.xhtml

2015-16 Report 2: Complications and Mortality. 2017.

73 National Cardiovascular Intelligence Network (NCVIN) - Public Health England. Prevalence estimates of diabetes. 2016.

74 Phillips LD. A theory of requisite decision models. *Acta Psychol (Amst)* Published Online First: 1984. doi:10.1016/0001-6918(84)90005-2

For peer review only

For peer review only - http://bmjopen.bmj.com/site/about/guidelines.xhtml

Figure 1 - Review flowchart of epidemiological models

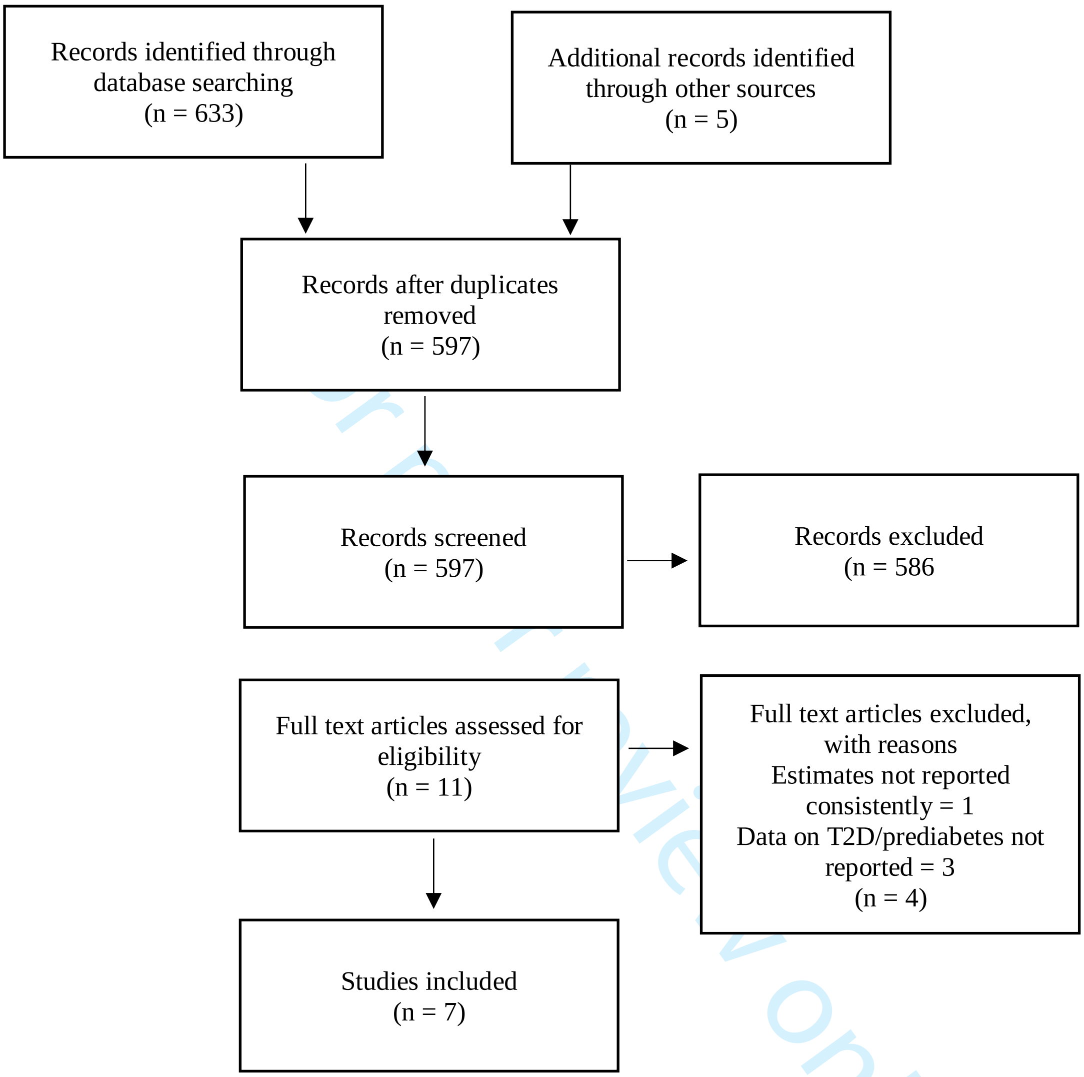

Figure 2 - Review flowchart of Markov chain models

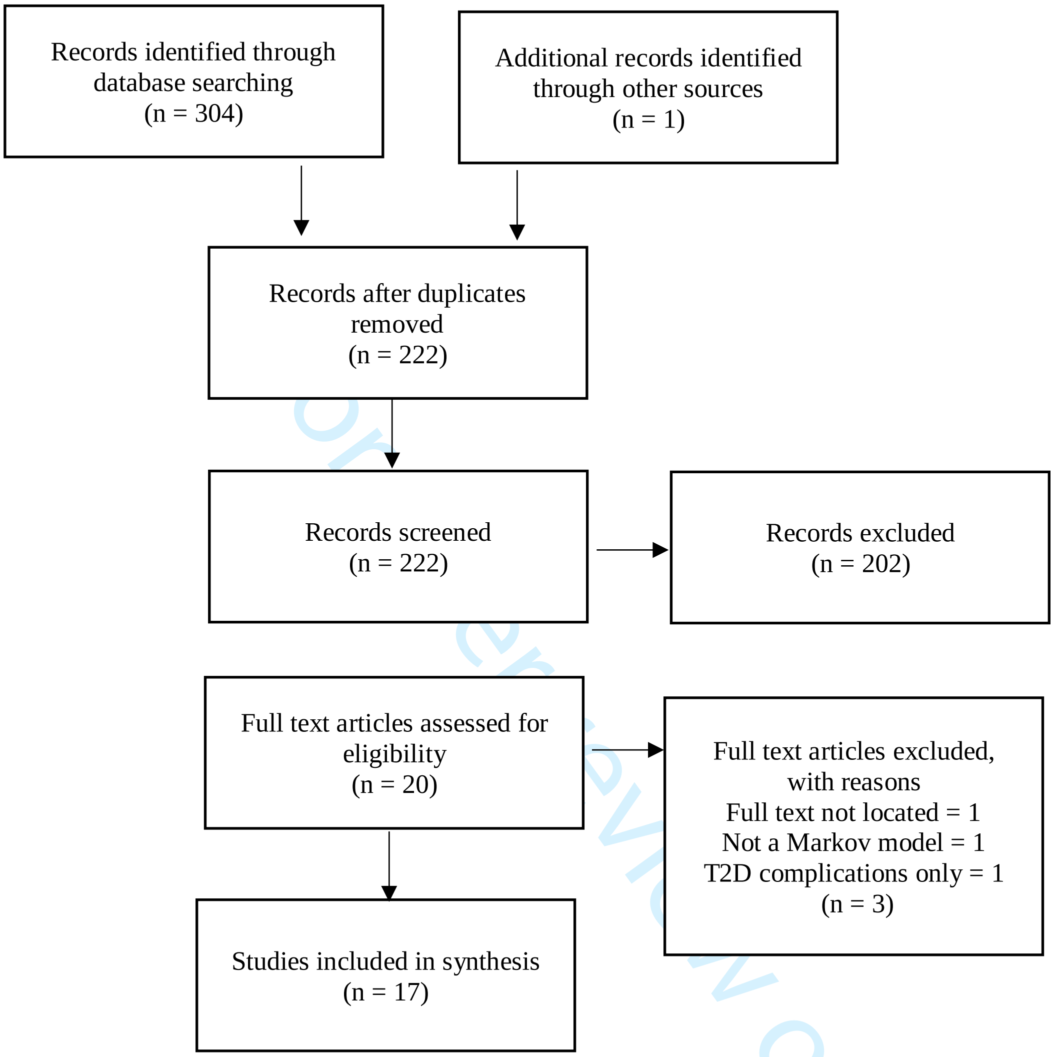

Figure 3 - Our Markov chain models

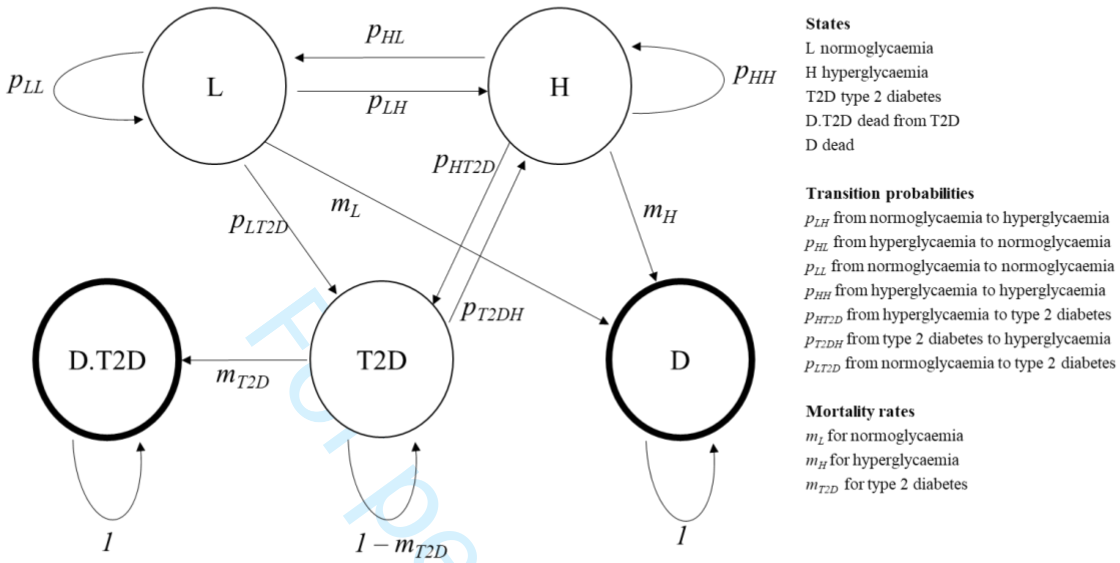

For peer review only

Figure 4 - Projections of true diabetes prevalence by PHE, Holman et al & from the QOF trend: 2005 to 2035

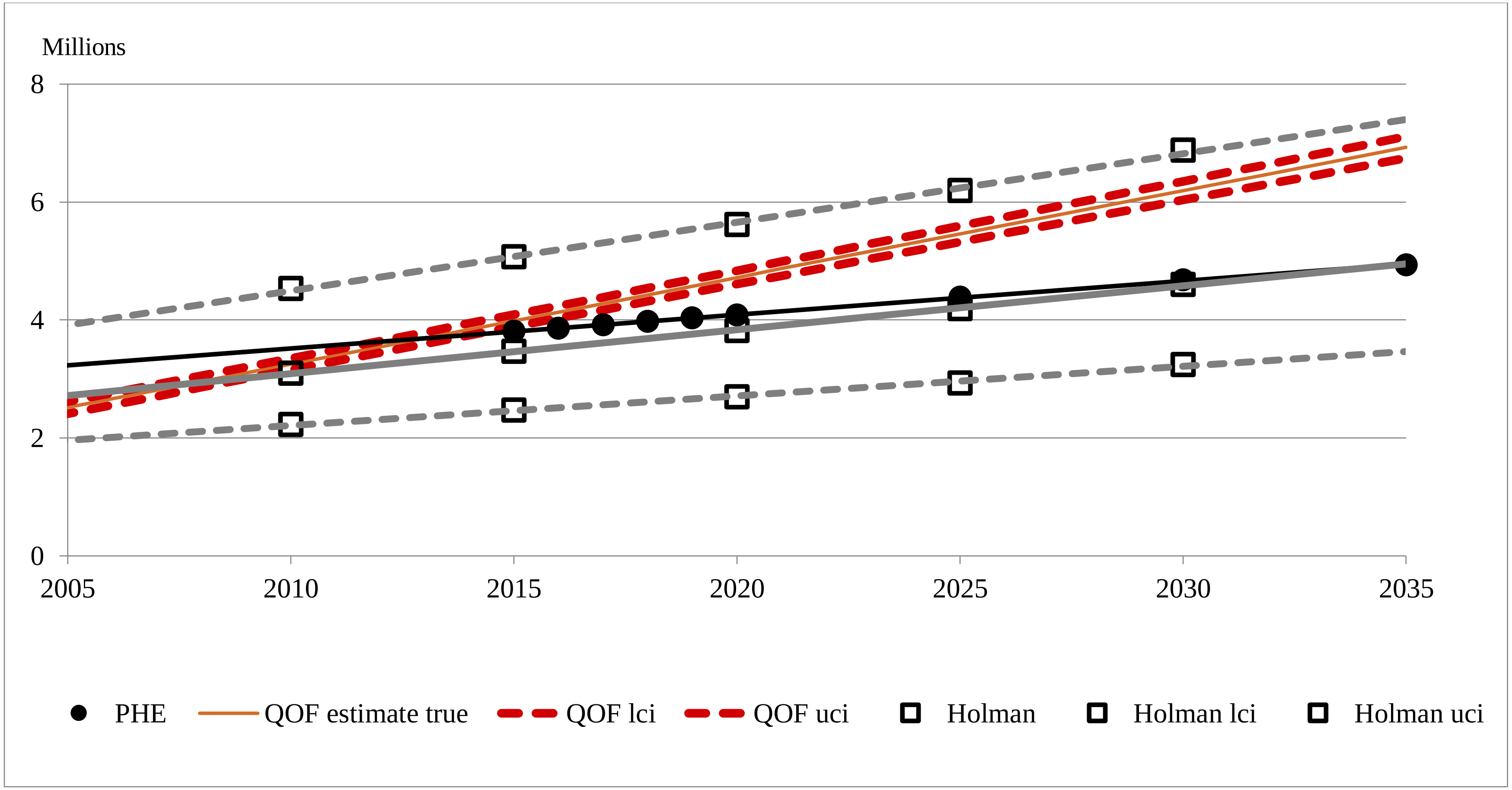

Figure 5 - Projections of adults with T2D in England: 2015 to 2035

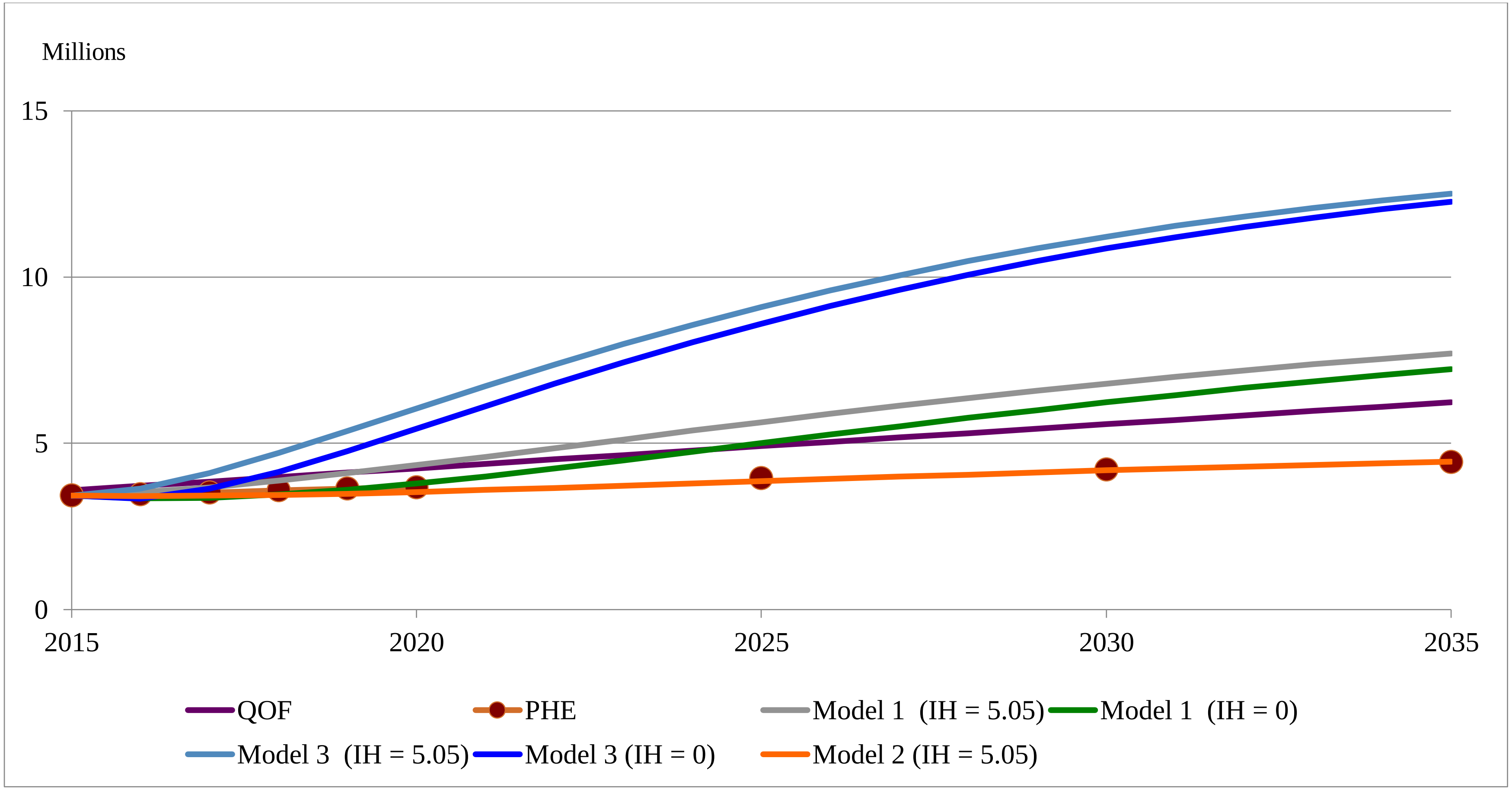

Appendix 1: Tables giving details of models

Index

Table 1.1: Search strategy of the rapid reviews

Table 1.2: Measures of intermediate hyperglycaemia used in Markov chain models

Table 1.3: Data sources of estimates used by our Markov Chain models

Table 1.4: Ratios used for comparing different estimates

Table 1.5: The three sets of transition probabilities used in different models

For peer review only

Table 1.1: Search strategy of the rapid reviews

**Epidemiological models**	
**Web of Science**	from All Databases<br>You searched for: TOPIC: ("diabet*" OR "type 2 diabetes" OR "diabetes mellitus") AND TITLE: ("Engl*" or "United Kingdom" or "UK") AND TOPIC: ("model" or "simulation" or "project*") AND TOPIC: ("epidemiolog*" or "prevalence" or "incidence" or "trend*") NOT TITLE: ("child*")<br>Refined by: LANGUAGES: (ENGLISH)<br>Timespan: All years.<br>Search language=Auto
**PubMed**	((((((("diabet*" OR "type 2 diabetes" OR "diabetes mellitus")) AND ("Engl*" OR "UK" OR "United Kingdom")) AND ("model" OR "simulation" OR "project*")) AND ("epidemiolog*" OR "prevalence" OR "incidence" OR "trend*")) NOT "child*" AND Humans[Mesh]) AND Humans[Mesh] AND English[lang] AND (Humans[Mesh] AND English[lang])
**Markov chain models**	
**Web of science**	TITLE: ("diabet*" OR "type 2 diabetes" OR "diabetes mellitus" or "pre-diabetes" or "prediabetes") AND TITLE: ("economic evaluation" or "cost-effectiveness" or "cost effectiveness" or "cost-utility" or "cost utility") AND TOPIC: ("Markov") NOT TOPIC: ("child*")<br>Refined by: LANGUAGES: (ENGLISH)<br>Timespan: All years.<br>Search language=Auto
**PubMed**	**((("diabet*" OR "type 2 diabetes" OR "diabetes mellitus" OR "prediabetes" OR "pre-**diabetes") AND ("economic evaluation" OR "cost-effectiveness" OR "cost effectiveness" OR "cost-utility" OR "cost utility")) AND "Markov" NOT "child") AND ("humans"[MeSH Terms] AND English[lang])

For peer review only - http://bmjopen.bmj.com/site/about/guidelines.xhtml

Table 1.2: Measures of intermediate hyperglycaemia used in Markov chain models

Measure of intermediate hyperglycaemia	Definition
Impaired fasting glucose (IFG)	• Diagnosed with an Oral glucose tolerance test (OGTT) performed after an overnight fast<br>• Defined by a fasting plasma glucose (FPG) concentration of<br>○ 5.6-6.9 mmol/L according to American Diabetes Association (ADA)[1]<br>○ 6.0-6.9 mmol/L according to the World Health Organization (WHO)[2]
Impaired glucose tolerance (IGT)	• Diagnosed with a 2-hour glucose tolerance test (2hrGTT), i.e. a blood test performed 2 hours after a 75-g glucose load<br>• Defined by 2-h plasma glucose concentration of<br>○ 7.8-11 mmol/L according to to American Diabetes Association (ADA)[1]<br>○ 7-11 mmol/L according to the World Health Organization (WHO)[2]
Glycated Haemoglobin (HbA1c)	• Diagnosed with the A1c test, measuring the average blood glucose over 2-3 months<br>• Defined by A1c concentration of<br>○ 39-47 mmol/mol (5.7-6.4%) according to to American Diabetes Association (ADA)[1]<br>○ 42-47 mmol/mol (6.0-6.4%) according to the World Health Organization (WHO)[3]

For peer review only

Table 1.3: Data sources of estimates used by our Markov Chain models

Estimate	Year(s)	Source
Estimated prevalence of intermediate hyperglycaemia (based on HbA1c)	2015	Public Health England[4]
Estimated prevalence of diabetes (both types)	2015	Public Health England[5]
Estimated prevalence of normoglycaemia: residual of the population for 2015	2015	Office of National Statistics[6]
Age distributions for those with intermediate hyperglycaemia & diabetes	Five years of combined data from 2009 to 2013	Health Surveys for England (HSE)[7]
Mortality rates by age	2015	Office of National Statistics[6]
Hazard ratios for those with diabetes & T2D	2015-16	National Diabetes Audit[8]
Hazard ratios for those for those with intermediate hyperglycaemia	Various years	Systematic review[9]

For peer review only

Table 1.4: Ratios used for comparing different estimates

Numerator	Denominator	Ratio	Sources
Diagnosed prevalence of diabetes	True prevalence of diabetes	75%	[10,11]
Prevalence of T2D	Prevalence of diabetes	90%	[12]
English population aged 20 to 79 in 2030 & 2035	UK population aged 20 to 79 in 2030 & 2035	• 2030: 87%<br>• 2035: 87%	[6]
Prevalence of diabetics aged over 15 (England) in 2030 & 2035	Prevalence of diabetics aged 20 to 79 (England) in 2030 & 2035	• 2030: 128%<br>• 2035: 129%	[7]
Prevalence of diabetics aged over 15 in England in 2030 & 2035	Prevalence of diabetics aged over 20 in UK in 2030 & 2035	• 2030: 0.87*1.28 = 111%<br>• 2035: 0.87*1.29 = 113%	[6, 7]

For peer review only

For peer review only - http://bmjopen.bmj.com/site/about/guidelines.xhtml

Table 1.5: The three sets of transition probabilities used in different models

	Model 1*	Model 2**	Model 3***
Normoglycaemia – Normoglycaemia	0.925	0.925	0.831
Normoglycaemia – Intermediate hyperglycaemia	0.069	0.069	0.163
Normoglycaemia – T2D	0.000	0.000	0.000
Normoglycaemia – Dead	0.006	0.006	0.006
Total	*1.000*	*1.000*	*1.000*
Intermediate hyperglycaemia -Intermediate hyperglycaemia	0.856	0.878	0.754
Intermediate hyperglycaemia- Normoglycaemia	0.090	0.090	0.162
Intermediate hyperglycaemia – T2D	0.036	0.013	0.060
Intermediate hyperglycaemia – Dead	0.019	0.019	0.024
Totals	*1.000*	*1.000*	*1.000*
T2D-T2D	0.977	0.977	0.974
T2D – Normoglycaemia	0.000	0.000	0.000
T2D- Intermediate hyperglycaemia	0.000	0.000	0.005
T2D – Dead	0.023	0.023	0.021
Total	*1.000*	*1.000*	*1.000*

Notes:
* Model 1is based on the transition probabilities from Roberts et al[13]for HbA1c.
** Model 2 is based on Model 1 modified to generate the PHE projections of the prevalence of T2D:the transition probability from intermediate hyperglycaemia to T2D of Model 2 (0.013) is a third of that of Model 1 (0.036); and has a corresponding increase in the transition probability of remaining as intermediate hyperglycaemia (0.836 to 0.878).
*** Model 3 is based on the transition probabilities from Neuman et al[14] for IGT.

References

1 American Diabetes Association (ADA). 2. Classification and Diagnosis of Diabetes: Standards of Medical Care in Diabetes. *Diabetes Care* 2018;**41**:S13–27.

2 World Health Organization (WHO). Definition and diagnosis of diabetes mellitus and intermediate hyperglycemia : report of a WHO/IDF consultation. Geneva (CH): 2006.

3 World Health Organization (WHO). Use of glycated haemoglobin (HbA1c) in the diagnosis of diabetes mellitus. 2011.

4 Public Health England - National Cardiovascular Intelligence Network. Prevalence estimates of non-diabetic hyperglycaemia. 2015.

5 National Cardiovascular Intelligence Network (NCVIN) - Public Health England. Prevalence estimates of diabetes. 2016.

6 Office for National Statistics. 2014-based National Population Projections. 2015.

7 National Cardiovascular Intelligence Network (NCVIN)- Public Health England. NHS Diabetes Prevention Programme (NHS DPP) Non-diabetic hyperglycaemia. 2015.

8 Healthcare Quality Improvement Partnership (HQIP). National Diabetes Audit, 2015-16 Report 2: Complications and Mortality. 2017.

9 Huang Y, Cai X, Mai W, *et al.* Association between prediabetes and risk of cardiovascular disease and all cause mortality: systematic review and

For peer review only - http://bmjopen.bmj.com/site/about/guidelines.xhtml

meta-analysis. *BMJ* 2016;**355**:i5953. doi:10.1136/bmj.i5953

10 Holman N, Forouhi NG, Goyder E, *et al.* The Association of Public Health Observatories (APHO) Diabetes Prevalence Model: Estimates of total diabetes prevalence for England, 2010-2030. *Diabet Med* 2011;**28**:575–82. doi:10.1111/j.1464-5491.2010.03216.x

11 Moody A, Cowley G, Fat LN, *et al.* Social inequalities in prevalence of diagnosed and undiagnosed diabetes and impaired glucose regulation in participants in the Health Surveys for England series. *BMJ Open* 2016;**6**:e010155. doi:10.1136/BMJOPEN-2015-010155

12 Gatineau M, Hancock C, Holman N, *et al.* Adult obesity and type 2 diabetes. London, UK: 2014.

13 Roberts S, Craig D, Adler A, *et al.* Economic evaluation of type 2 diabetes prevention programmes: Markov model of low- and high-intensity lifestyle programmes and metformin in participants with different categories of intermediate hyperglycaemia. *BMC Med* 2018;**16**:16. doi:10.1186/s12916-017-0984-4

14 Neumann A, Lindholm L, Norberg M, *et al.* The cost-effectiveness of interventions targeting lifestyle change for the prevention of diabetes in a Swedish primary care and community based prevention program. *Eur J Heal Econ* 2017;**18**:905–19. doi:10.1007/s10198-016-0851-9

For peer review only - http://bmjopen.bmj.com/site/about/guidelines.xhtml

Appendix 2: Rapid Reviews of Epidemiological and Markov chain models

Table 2.1 gives the search strategies for each review and Table 2 the details of our rapid review of Markov chain models and Table 2.2 gives details of our rapid review of Markov chain models.

Table 2.1: Search strategies for each review

**Epidemiological models**	
**Web of science**	TOPIC: ("diabet*" OR "type 2 diabetes" OR "diabetes mellitus") AND TITLE: ("Engl*" or "United Kingdom" or "UK") AND TOPIC: ("model" or "simulation" or "project*") AND TOPIC: ("epidemiolog*" or "prevalence" or "incidence" or "trend*") NOT TITLE: ("child*")<br>Timespan: All years.<br>Search language=Auto
**PubMed**	(((("diabet*"[All Fields] OR "type 2 diabetes"[All Fields] OR "diabetes mellitus"[All Fields] OR "pre-diabetes"[All Fields] OR "prediabetes"[All Fields]) AND ("economic evaluation"[All Fields] OR "cost-effectiveness"[All Fields] OR "cost effectiveness"[All Fields] OR "cost-utility"[All Fields] OR "cost utility"[All Fields])) AND "markov"[All Fields]) NOT ("child*"[All Fields] OR "pediatric"[All Fields] OR "paediatric"[All Fields])) NOT "type 1 diabetes"[All Fields] AND ("humans"[MeSH Terms] AND English[lang])
**Markov chain models**	
**Web of science**	TITLE: ("diabet*" OR "type 2 diabetes" OR "diabetes mellitus" or "pre-diabetes" or "prediabetes") AND TITLE: ("economic evaluation" or "cost-effectiveness" or "cost effectiveness" or "cost-utility" or "cost utility") NOT TOPIC: ("child*" or "pediatric" or "paediatric") NOT TOPIC: ("type 1 diabetes") AND TOPIC: ("markov")<br>Refined by: LANGUAGES: (ENGLISH)<br>Timespan: All years.<br>Search language=Auto
**PubMed**	(((("diabet*"[All Fields] OR "type 2 diabetes"[All Fields] OR "diabetes mellitus"[All Fields] OR "pre-diabetes"[All Fields] OR "prediabetes"[All Fields]) AND ("economic evaluation"[All Fields] OR "cost-effectiveness"[All Fields] OR "cost effectiveness"[All Fields] OR "cost-utility"[All Fields] OR "cost utility"[All Fields])) AND "markov"[All Fields]) NOT ("child*"[All Fields] OR "pediatric"[All Fields] OR "paediatric"[All Fields])) NOT "type 1 diabetes"[All Fields] AND ("humans"[MeSH Terms] AND English[lang])

For peer review only

Table 2.2: Details of our rapid review of Markov chain models

Author	Type	Country	Risk measure	Objectives of the model	Description of the Markov model	Transition probabilities	Mortality rates	Population modelled	Outcomes	Results in terms of the number of cases prevented under "no intervention"	Sensitivity analysis	Model validation
Caro et al 2004	EA	Canada	IGT	- To compare the health and economic outcomes of acarbose, an intensive lifestyle modification programme, metformin or no intervention to prevent progression to diabetes	A Markov model to simulate long-term outcomes in a cohort of patients with IGT under each of four treatment strategies. The cohort is followed for a 10- year period in the base case analyses. The model cycles over 6-month periods. Four main states were considered: IGT, diabetes, normal glucose tolerance (NGT) and death. Patients who revert to NGT may develop IGT again, while patients who develop diabetes are assumed to remain in that state until death.	Reported, originally developed for the model	Estimated based on age- and gender-specific death hazards calculated from Canadian life table data and increased by 45% to take into account the effect of IGT. Upon reverting to NGT, patients were assumed to lose the increased risk.	For the base case, patient characteristics were taken from the STOP-NIDDM trial [12]. Just over half of patients in that trial were male, and the mean age at the start of the trial was 54.5 years.	No of patients transitioning to T2D<br>No who reverted and remained NGT<br>Life expectancy<br>Years free of T2D	For a cohort of 1000 patients, over the course of 10 years, 542 untreated patients with IGT are expected to develop diabetes, while 242 will have returned to NGT	Performed, results for base case not available	Not available
Chen et al, 2001	CEA	Taiwan	NA	- To develop the natural history of T2D<br>- To quantify the efficacy of early detection of T2D in slowing or reducing the progression of complications<br>- To evaluate the effect of inter-screening interval and age at the start of screening on slowing/reducing the progression of complications or deaths<br>- To compare the cost and effectiveness of a screening regime<br>- To assess the cost–effectiveness of T2D screening by age-specific groups and different inter-	A Markov model to simulate the natural history of T2D from normal, onset of DM, clinical complications, deaths.<br>Disease progression modules from onset of DM to complications include three parts: Retinopathy, Nephropathy, and Neuropathy.	Not reported<br>Transition parameters used for simulating disease progression refer to Eastman et al., Javitt at al., Harris et al., Klein et al., Ballard et al., Humphrey et al. , USRD, Dyck et al., Humphrey et al., and CDC–DCS group.	Not provided<br>- Life-table information was used to adjust for competing causes of deaths<br>- Mortality rates for diabetes complications retrieved from the literature	A hypothetical cohort with 30,000 adults aged over 30	Life-years gained<br>QALYs	Not available	Not available	Not available

For peer review only

Author	Type	Country	Risk measure	Objectives of the model	Description of the Markov model	Transition probabilities	Mortality rates	Population modelled	Outcomes	Results in terms of the number of cases prevented under “no intervention”	Sensitivity analysis	Model validation
				screening interval.								
Gillies et al, 2008	CEA	UK	IGT	To compare potential screening strategies, and subsequent interventions, for the prevention and treatment of T2D<br>- screening for T2D to enable early detection and treatment<br>- screening for T2D and impaired glucose tolerance, intervening with lifestyle interventions in those with a diagnosis of impaired glucose tolerance<br>- as for (b) but with pharmacological interventions<br>- no screening	Hybrid model consists of a decision tree and a Markov model<br>The decision tree comprises three main arms, representing no screening, screening for undiagnosed T2D, and screening for impaired glucose tolerance and undiagnosed diabetes, with either lifestyle or pharmacological interventions applied in those with impaired glucose tolerance<br>The Markov model consists of seven states: normal glucose tolerance, undiagnosed impaired glucose tolerance, diagnosed impaired glucose tolerance, death, and three states for people with diabetes (undiagnosed, diagnosed clinically, or diagnosed through screening, either from a screening test or because they are diagnosed with impaired glucose tolerance initially and hence enter a surveillance programme)<br>Each model cycle represents one year and the model is run for a time horizon of 50 years.	Reported	Increased risk of death with diabetes<br>(hazard ratio) 0.756 SE - (0.087)<br>Increased risk of death for 1% increase in HbA1c (hazard ratio) 0.104 (SE =0.039	Hypothetical population, aged 45 at time of screening, with above average risk of diabetes..	Clinical and cost outcomes	Not available	Performed, results available	Not available

For peer review only

Author	Type	Country	Risk measure	Objectives of the model	Description of the Markov model	Transition probabilities	Mortality rates	Population modelled	Outcomes	Results in terms of the number of cases prevented under "no intervention"	Sensitivity analysis	Model validation
Herman et al, 2005	CEA	USA	IGT	To estimate the lifetime cost–utility of the DPP interventions.	Markov model originally developed by the Centers for Disease Control and Prevention and Research Triangle Institute International to assess the progression from impaired glucose tolerance to onset of diabetes to clinically diagnosed diabetes to diabetes with complications and death by using a lifetime simulation model.	Not reported	Not reported	Members of the DPP cohort 25 years of age or older with impaired glucose tolerance.	Progression of disease<br>Costs<br>Quality of life	If the entire DPP cohort were treated with the placebo intervention, approximately 50% of individuals would develop diabetes within 7 years. Over a lifetime conversion rate from IGT to T2D is 82.8%	Performed, results available	Not available
Ikeda et al, 2010	CEA	Japan	IGT	To estimate the cost-effectiveness of administering voglibose, in addition to standard care of diet and exercise, compared with standard care alone for high-risk Japanese patients with impaired glucose tolerance	Markov model consisting of five stages: normal glucose tolerance, IGT, T2DM, dialysis and death	Available only for transition from NGT to IGT	For the annual mortality of NGT, the average values for males and females in the national data of the abridged life table in 2008 were used Relative risk of death in IGT and T2DM in comparison with NGT was set as 1.35 and 3.03, respectively.	The age of the IGT population was set as 56, corresponding to the average age in the voglibose clinical trial population,	- Long-term costs<br>- Life expectancy<br>- Cost-effectiveness	Not available	Performed, results available	Not available
Johansson et al, 2009	CEA	Sweden	FPG	To estimate the cost-effectiveness of a community-based program promoting general population lifestyle changes to prevent diabetes.	Markov model constructed to reflect the metabolic syndrome, covers adults, with the termination age set at 85 years, after which no further health effects or costs are accumulated Model is fully described in a separate technical report	Not reported	Not reported Mortality risks were taken from Swedish registers, and included both disease-related mortality as well as unrelated mortality	Population group aged 36–56 years at baseline	- Costs<br>- QALYs	Not available	Performed, results available	Not available
Liu et al, 2013	EA	China	IGT	To estimate the clinical and economic outcomes of screening for undiagnosed diabetes and impaired glucose tolerance (IGT), followed by the implementation of lifestyle intervention in those with IGT.	Hybrid decision tree Markov model. The decision tree included five arms representing five scenarios. The first three scenarios involved screening for undiagnosed diabetes and IGT followed by one of the three active lifestyle interventions (diet, exercise or duo-intervention), which	Reported	Not reported The life-table information was used to evaluate the competing causes of death at the different initiation ages	A representative sample of Chinese adults was used to create a simulated population of 20,000 people aged 25 years and above.	- Remaining survival years QALYs per subject with diabetes or IGT<br>- Life-years gained before the onset of diabetes or before the onset of any complication per subject with IGT<br>- Cost per subject for prevention strategies or	Not available	Performed, results available.	Performed, not reported

For peer review only - http://bmjopen.bmj.com/site/about/guidelines.xhtml

Author	Type	Country	Risk measure	Objectives of the model	Description of the Markov model	Transition probabilities	Mortality rates	Population modelled	Outcomes	Results in terms of the number of cases prevented under "no intervention"	Sensitivity analysis	Model validation
					were applied to the IGT subjects. The fourth scenario involved screening for undiagnosed diabetes and IGT, without the formal lifestyle interventions. The fifth scenario involved the control group with no screening or intervention.<br><br>The decision tree used positive screening rates and the prevalence of diabetes and IGT in the reference population to determine how many individuals started in each state of the Markov models. Each Markov model consisted of eight main health states: IGT, normal glucose tolerance, onset of diabetes, four diabetes complication states and death. The Markov models ran for a time horizon of 40 years, and each of the model cycles represented 1 year. Separate simulations with different incidence rates of diabetes, mortality rates and health utilities were performed for the diabetes prevention programmes or for the control starting at 25, 40 and 60 years, respectively.				control at different initiation ages.			
Neumann et al, 2011	CEA	Germany	IGT	To investigate the long-term cost-effectiveness of lifestyle intervention programmes for the prevention of T2D	Four-state Markov modelling with a probabilistic cohort analysis : normal glucose tolerance (NGT), impaired glucose tolerance	Reported	Not reported Mortality Life tables provide the mortality rates for different ages and sexes. Eight different mortality categories, by age and	The prevalence of IGT among the general German population is used as the base for the model, with 16% of individuals having	- Cost per quality-adjusted life year (QALY)	Not available	Performed, results available	Not available

For peer review only

Author	Type	Country	Risk measure	Objectives of the model	Description of the Markov model	Transition probabilities	Mortality rates	Population modelled	Outcomes	Results in terms of the number of cases prevented under "no intervention"	Sensitivity analysis	Model validation
					(IGT), diagnosed type 2 diabetes mellitus (T2D), or death. A one-year cycle length and a lifetime time horizon are applied.		sex, are established: less than 35, 35-64, 65-74, and 75 years and over for men and women. Mortality statistics were obtained from the Statistical Office of the Federal State of Saxony Transition probability of a person with T2D dying from T2D. adjusted using the data on all-cause deaths attributable to diabetes from the study by Roglic et al [25].	IGT, 84% NGT and no one T2D.				
Neumann et al, 2017	CEA	Sweden	IFG IGT	To estimate the cost-effectiveness of a T2D prevention initiative targeting weight reduction, increased physical activity and healthier diet in persons in pre-diabetic states by comparing a hypothetical intervention versus no intervention in a Swedish setting.	The model consisted of six different, mutually exclusive states: NGT, IFG, IGT, IFG and IGT, T2D and death. The length of one cycle was 1 year. A lifetime horizon was applied.<br><br>As it was assumed that 1 year was too short to develop T2D directly from NGT, this transition was not possible. Hence, all hypothetical persons must have developed any of the three pre-diabetic states before the development of T2D.	Not reported	Age-based all-cause mortality and mortality due to T2D were taken from Statistics Sweden and the National Board of Health and Welfare based on the years 2003–2009 Five-year age ranges were estimated No increased risk of dying due to any of the pre-diabetic states was assumed.	Not reported Based on the Vasterbotten Intervention Program (VIP)	- QALY<br>- Incremental cost-effectiveness ratios (ICERs)	Not available	Performed, results available	Not available
Palmer et al, 2012	CEA	Australia	IGT	To examine the long-term cost-effectiveness of the control, metformin and ILC interventions in the DPP for a cohort of subjects at high risk of developing type 2 diabetes in an Australian healthcare setting	Semi-Markov model, with four health states: 'normal glucose regulation' (NGR) (plasma glucose con-centration <5.6 mmol/L in fasting state or <7.8 mmol/L 2 h after a 75 g oral glucose load); 'impaired glucose tolerance' (IGT) (fasting plasma glucose concentration 5.6–6.9 mmol/L or 7.8–11.0 mmol/L 2 h after a 75 g oral	Reported	Annual mortality rates were calculated from Australian sex- and age-specific life tables and were state-dependent, but independent of treatment arm All-cause mortality rates in the NGR state were applied unadjusted Relative mortality risks for subjects in the IGT, "undiagnosed" diabetes or "diagnosed" diabetes	A hypothetical cohort was defined with baseline characteristics in keeping with the Diabetes Prevention Program (DPP) study: mean age 50.6 years; 32.2% male; mean body mass index 34.0 kg/m2; and IGT present.	- Cumulative incidence<br>- Lifetime incremental direct costs<br>- Incremental costs per QALY-gained	Mean cumulative incidence (95% CI) of type 2 diabetes in the control, metformin and ILC treatment arms estimated at 89.7% (89.4–90.1), 83.8% (83.3–84.3) and 73.4 (72.8–74.1), respectively	Performed, results available	Internal validation performed, results available

For peer review only

Author	Type	Country	Risk measure	Objectives of the model	Description of the Markov model	Transition probabilities	Mortality rates	Population modelled	Outcomes	Results in terms of the number of cases prevented under "no intervention"	Sensitivity analysis	Model validation
					glucose load); 'type 2 diabetes' (T2D) (plasma glucose concentration at least 7.0 mmol/L or 11.1 mmol/L 2 h after a 75 g oral glucose load),'dead'. Each cycle in the model represented one year of a simulated subject's life and at the end of each cycle, subjects could remain in the same state, progress to another state or die. The simulation ran over subject lifetimes		states were 1.50 (95% CI 1.10–2.00), 1.30 (0.90–2.66) and 2.30 (1.60–3.20), respectively					
Palmer et at, 2004	EA	Australia France Germany Switzerland UK	IGT	To establish whether implementing the active treatments used in the DPP would be cost-effective in the selected countries.	Markov model consisting of 3 states: IGT (as defined in the DPP), type 2 DM, and deceased. Simulated patients initially had IGT and progressed at differing rates to T2S depending on the treatment received. A patient lifetime horizon was used.	Reported	Partially reported The probability of death associated with IGT or T2D was calculated from age- and sex-dependent country-specific, national, all-cause mortality tables, adjusted using published relative risks (RRs) for all-cause mortality for patients with IGT versus normo-glycemic patients.	The cohort of patients in this analysis was constructed to resemble the study population of the DPP (mean age, 50.6 years; mean body weight, 94.2 kg; mean body mass index [BMI], 34.0 kg/m2; men, 32.2%)	- No of years free of DM - Percentage of patients developing DM - Life expectancy - Total lifetime costs per patient	Not available	Performed, results available	Not available
Roberts et al, 2018	EA	England	IFG IGT HbA1c	To examine the costs and effects of different intensity lifestyle programmes and metformin in participants with different categories of intermediate hyperglycaemia	Decision tree and Markov model (50-year horizon) to compare four approaches: (1) a low-intensity lifestyle programme based on current NICE guidance, (2) a high-intensity lifestyle programme based on the US Diabetes Prevention Program, (3) metformin, and (4) no intervention, modelled for three different types of intermediate hyperglycaemia (IFG, IGT and HbA1c).	Reported	Not reported All-cause age-standardised mortality rates were determined from the Office of National Statistics in England, with increased risk of death calculated for participants with intermediate hyper-glycaemia or T2DM	Not described	Impact on an individual participant in a prevention programme: (1) discounted cumulative healthcare costs (including costs of diagnostic tests and primary and secondary care associated with the intervention, intermediate hyperglycaemia, T2DM and complications of T2DM), (2) discounted QALYs, (3) incidence of T2DM, (4) average number of years with T2DM, (5) cost-effectiveness ratios in £/QALY, and (6)	With no intervention, 42% of the IGT population and 38% of the IFG and HbA1c population developed T2DM over 50 years.	Performed, results available	Performed, reported

For peer review only

Author	Type	Country	Risk measur e	Objectives of the model	Description of the Markov model	Transition probabilities	Mortality rates	Population modelled	Outcomes	Results in terms of the number of cases prevented under "no intervention"	Sensitivity analysis	Model validation
									incremental cost-effectiveness ratios (ICERs), in £/QALY (for non-dominated interventions). Impact of a nation-wide prevention programme: (1) discounted annual incremental costs, (2) discounted cumulative incremental costs, (3) discounted incremental costs as a percentage of the total diabetes expenditure [17], and (4) cumulative incidence of T2DM.			
Schaufler et al, 2010	CEA	Germany	OGTT	To examine the cost effectiveness of screening for T2DM in Germany	Markov model to reproduce the time-discrete stochastic process using a 1 year cycle	Reported	Not reported General mortality rates were derived from the official German mortality tables The higher mortality for patients with diabetes than for the general population, especially after cardio-vascular events, was accounted for by applying relative risks drawn from Roper et al.[28]	Not described	- Quality of life (QOL)<br>- Lifetime costs<br>- Age at diabetes diagnosis<br>- Incidence and age at occurrence of diabetes-related complications.	Not available	Performed, results available	Performed, reported
Smith et al 2010	CEA	USA	IFG	To assessed the cost-effectiveness of a modified version of the US DPP	Markov model with six states: risk factor negative (no diabetes), risk factor positive (enrolled in mDPP), risk factor positive (not enrolled in mDPP), stable T2D, complications, death	Partially reported	Mortality rates based on age- and sex-specific US mortality (which accounts for baseline mortality) and the relative risks of death for metabolic syndrome, stable diabetes, and complicated diabetes<br>- risk factor positive 1.7 (Lakka et al 2002)<br>- risk factor negative 1 (own assumption)<br>- stable T2D 2 (Moss et al 1991)	In the model, we used a base case that examined 55-year-old men and women at monthly intervals for 3 years. 75% women	- Metabolic syndrome risk at 1 year<br>- Costs<br>- QALYs<br>- T2D incidence	Without the mDPP, 9.6% of the cohort developed diabetes over 3 years	Performed, results for base-case not reported	Not available

For peer review only

Author	Type	Country	Risk measure	Objectives of the model	Description of the Markov model	Transition probabilities	Mortality rates	Population modelled	Outcomes	Results in terms of the number of cases prevented under "no intervention"	Sensitivity analysis	Model validation
							- complicated T2D 2.4 (Fuller et al 2001)					
Wong et l, 2016	CEA	Hong Kong	IGT	To investigate the costs and cost-effectiveness of a short message service (SMS) intervention to prevent the onset of type 2 diabetes mellitus (T2DM) in subjects with impaired glucose tolerance (IGT).	Markov model with one-year transition cycle with four Markov states: normal glucose tolerance (NGT), IGT, T2DM, and death. Long-term modelling referred to time horizon over a 50-year period beyond the two year intervention	Reported	All-cause mortality rates for NGT were adopted from the Hong Kong Life Table 2011<br>The relative risks of mortality in IGT and T2DM were 1.50 (95% CI 1.10–2.00) and 2.30 (95%CI 1.60–3.20), respectively, which were used to adjust the age-specific death rate for subjects with IGT or T2DM [23]	Not reported	- Costs<br>- QALYs	Not available	Performed, results available	Not available
Zhou et al. 2005	CEA	USA	IGT	To develop and validate a comprehensive computer simulation model to assess the impact of screening, prevention, and treatment strategies on T2D and its complications, comorbidities, quality of life, and cost.	Markov model with four states: NGT, IGT, T2D, death.	Not reported	Not reported<br>Estimates of mortality are taken from the age-, sex-, and race-specific mortality for the U.S. population.	Not reported	- Health states<br>- Utilities<br>- Costs	Not available	Not available	Validated usning the WESDR is a population- based study of individuals with diabetes the WESDR cohort with type 2 diabetes in southern Wisconsin.
Zhuo et al, 2012	CEA	USA	HbA1c	To examine the change in the cost effectiveness of diabetes-preventive interventions because of progressive 0.1% decremental reductions in the HbA1c cutoff from 6.4% to 5.5%.	Markov model described in a report by Herman et al.	Not reported	Not reported	A nationally representative sample of U.S. adults (aged 18 years) from the 1999–2006 National Health and Nutrition Examination Survey (NHANES)	Cost effectiveness associated with the HbA1c cutoffs was measured as cost per QALY gained	Not available	Performed, results available	Performed, not reported. International

For peer review only

How valid are projections of the future prevalence of diabetes? Rapid reviews of prevalence-based and Markov chain models and comparisons of different models' projections for England

Journal:	*BMJ Open*
Manuscript ID	bmjopen-2019-033483.R2
Article Type:	Original research
Date Submitted by the Author:	23-Dec-2019
Complete List of Authors:	Bevan, Gwyn; London School of Economics and Political Science, Management<br>De Poli, Chiara; London School of Economics and Political Science, Management<br>Keng, Mi; University of Oxford, Nuffield Department of Population Health<br>Raine, Rosalind; University College London, Department of Applied Health Research
**Primary Subject Heading**:	Diabetes and endocrinology
Secondary Subject Heading:	Epidemiology, Health economics, Health services research, Health policy, Public health
Keywords:	Diabetes Mellitus, Prevalence, Markov Chains, Obesity, EPIDEMIOLOGY

SCHOLARONE™
Manuscripts

I, the Submitting Author has the right to grant and does grant on behalf of all authors of the Work (as defined in the below author licence), an exclusive licence and/or a non-exclusive licence for contributions from authors who are: i) UK Crown employees; ii) where BMJ has agreed a CC-BY licence shall apply, and/or iii) in accordance with the terms applicable for US Federal Government officers or employees acting as part of their official duties; on a worldwide, perpetual, irrevocable, royalty-free basis to BMJ Publishing Group Ltd ("BMJ") its licensees and where the relevant Journal is co-owned by BMJ to the co-owners of the Journal, to publish the Work in this journal and any other BMJ products and to exploit all rights, as set out in our licence.

The Submitting Author accepts and understands that any supply made under these terms is made by BMJ to the Submitting Author unless you are acting as an employee on behalf of your employer or a postgraduate student of an affiliated institution which is paying any applicable article publishing charge ("APC") for Open Access articles. Where the Submitting Author wishes to make the Work available on an Open Access basis (and intends to pay the relevant APC), the terms of reuse of such Open Access shall be governed by a Creative Commons licence – details of these licences and which Creative Commons licence will apply to this Work are set out in our licence referred to above.

Other than as permitted in any relevant BMJ Author's Self Archiving Policies, I confirm this Work has not been accepted for publication elsewhere, is not being considered for publication elsewhere and does not duplicate material already published. I confirm all authors consent to publication of this Work and authorise the granting of this licence.

For peer review only

How valid are projections of the future prevalence of diabetes? Rapid reviews of prevalence-based and Markov chain models and comparisons of different models' projections for England

Gwyn Bevan*

emeritus professor of policy analysis

Department of Management, London School of Economics and Political Science, WC2A 2AE, London, UK.

Email: R.G.Bevan@lse.ac.uk

Mob: +44 (0)77867 88967

Chiara De Poli

Research fellow

Department of Management, London School of Economics and Political Science, WC2A 2AE, London, UK.

Mi Jun Keng

researcher

Nuffield Department of Population Health, University of Oxford, OX3 7LF, Oxford, UK.

Rosalind Raine

professor of applied health research

Department of Applied Health Research, UCL, WC1E 7HB, London, UK

* Corresponding author

For peer review only - http://bmjopen.bmj.com/site/about/guidelines.xhtml

How valid are projections of the future prevalence of diabetes? Rapid reviews of prevalence-based and Markov chain models and comparisons of different models' projections for England

Abstract

Objectives

To examine validity of prevalence-based models giving projections of prevalence of diabetes in adults, in England and the UK, and of Markov chain models giving estimates of economic impacts of interventions to prevent type 2 diabetes (T2D).

Methods

Rapid reviews of both types of models. Estimation of the future prevalence of T2D in England: by Markov chain models; and from the trend in the prevalence of diabetes, as reported in the Quality and Outcomes Framework (QOF), estimated by Ordinary Least Squares regression analysis.

Setting

Adult population in England and UK.

Main outcome measure

Prevalence of T2D in England and UK in 2025.

Results

The prevalence-based models reviewed use sample estimates of past prevalence rates by age and sex and projected population changes. Three most recent

For peer review only

For peer review only - http://bmjopen.bmj.com/site/about/guidelines.xhtml

models, including that of Public Health England (PHE), neither take account of increases in obesity, nor report confidence intervals. The Markov chain models reviewed use transition probabilities between states of risk and death, estimated from various sources. None of their accounts give the full matrix of transition probabilities, and only a minority report tests of validation. Their primary focus is on estimating the ratio of costs to benefits of preventive interventions in those with hyperglycaemia, only one reported estimates of those developing T2D in the absence of a preventive intervention in the general population.

Projections of the prevalence of T2D in England in 2025 were (in millions) by PHE, 3.95; from the QOF trend, 4.91; and by two Markov chain models, based on our review, 5.64 and 9.10.

Conclusions

To inform national policies on preventing T2D, governments need validated models, designed to use available data, which estimate the scale of incidence of T2D and survival in the general population, with and without preventive interventions.

Article summary

Strengths and limitations of this study

- We undertook rapid reviews of prevalence-based models and Markov chain models, which have been used to give projections of the future prevalence of diabetes to examine their data sources and assumptions.
- We compared projections of the future prevalence of diabetes in England from: reports for the prevalence-based models; our own Markov chain models (based on transition probabilities from our review); and the trend in the prevalence of diagnosed diabetes as reported by general practitioners in England (estimated by ordinary least squares regression analysis).

For peer review only - http://bmjopen.bmj.com/site/about/guidelines.xhtml

- This study's limitations are that our reviews were rapid and our models are transparent and simple.

Keywords

Diabetes Mellitus

Prevalence

Forecasting

Markov Chains

Obesity

4,327 words

For peer review only

How valid are projections of the future prevalence of diabetes? Rapid reviews of prevalence-based and Markov chain models and comparisons of different models' projections for England

Introduction

Rigorous analysis of worldwide trends of increases in the preventable onset of Type 2 Diabetes (T2D) in adults justifies a call for the urgent of implementation of 'population-based interventions that prevent diabetes, enhance its early detection, and use lifestyle and pharmacological interventions to prevent or delay its progression to complications'.[1] In March 2015, NHS England and Public Health England (PHE) launched, at scale, the NHS Diabetes Prevention Programme (NDPP), which is a pragmatic lifestyle intervention that targets adults with raised levels of Glycated Haemoglobin (HbA1c) or a Fasting Plasma Glucose (FPG) [2]. The NDPP aims 'to significantly reduce the 4 million people in England otherwise expected to have Type 2 Diabetes (T2D) by 2025' based on evidence from 'well-designed randomised controlled trials (RCTs) in Finland, the USA, Japan, China and India'.[3] Many studies have used Markov chain models to estimate the impacts of such preventive interventions using transition probabilities between states: 'normoglycaemia' and 'intermediate hyperglycaemia' (glucose levels associated with a low and high risks of developing T2D), T2D and death. When we tried to use these models,[4] we had difficulty in finding details from published models, and the models we did develop gave projections of the future prevalence of T2D in 2025 in England, in the absence of a preventive intervention, that were much higher than 4 million. That estimate is based on PHE's prevalence-based model [5] that gives future projections of the prevalence of T2D (at future time t, N(t)) by multiplying projections of the country's population by age and sex (at time t (**P**(t)) by projections of age-specific prevalence of diabetes (at time t, **D**(t)). (N(t) = **D**(t)* **P**(t)).) Hence this study, which had three aims. First, to compare the model used by PHE to project the prevalence of diabetes in England with other models

For peer review only - http://bmjopen.bmj.com/site/about/guidelines.xhtml

applied to England and the UK. Second, to identify Markov chain models we could use to project the prevalence of T2D in England. Third, to compare projections for England of prevalence of diabetes and T2D from different models.

Although we have used England for the purpose of comparing projections by these different models, our study raises general questions about their validity. And hence of the evidence available to governments assessing the urgency of preventing T2D and choosing between different interventions. We consider only adults with diabetes. We use 'diabetes' to cover all types of diabetes, T2D for adults with type 2, 'true' prevalence for both diagnosed and undiagnosed diabetes and T2D.

Methods

Rapid reviews

Our comparisons of projections of different models builds on two reviews of the literature, which were designed to be rapid (not systematic): 'a type of knowledge synthesis in which components of the systematic review process are simplified or omitted to produce information in a short period of time'.[6] We used stringent criteria to identify the principal methods of each type of model. These reviews were undertaken in March 2018, of articles published at any time available on Web of science and PubMed, which together provide a comprehensive coverage of the literature in the medical and applied health research fields. (The search strategy of each review is given in Appendix 1.) Articles included in each review were critically appraised and technical specifications of the models and projections were extracted and tabulated. The flowcharts in Figures 1 and 2 show the screening process.

Figure 1 - Review flowchart of epidemiological models to go about here

Figure 2: Review flowcharts of Markov chain models to go about here

For peer review only - http://bmjopen.bmj.com/site/about/guidelines.xhtml

Review 1 aimed to identify primary studies published from 2010 of models giving estimates of the prevalence of diabetes in adults in England or the UK. We examined how the models take account of future changes in age-specific prevalence rates and test their validity.

Review 2 aimed to identify primary studies using Markov chain models that reported results of interventions to prevent T2D. We included articles using Markov models to run economic analyses, utility analyses and cost effectiveness analyses of interventions targeting people diagnosed with T2D, or with intermediate hyperglycaemia according to different measures: HbA1c, IFG, Impaired Glucose Tolerance (IGT), Oral Glucose Tolerance Test (OGTT) and Fasting Plasma Glucose (FPG). (Definitions are given in Appendix 1.) We reviewed the transition probabilities of the different models, whether they were used to estimate the future prevalence of T2D without a preventive intervention, and tests of validation. In our discussion, we refer to have the systematic review by Leal et al [7] of models of prediabetes populations used for reported economic outcomes or evaluations, which has been recently published.

Our Markov chain models

Our Markov chain models are in Excel (see Figure 3) and based on a cycle length of 1 year. The transition probabilities between states other than death are based on review 2 (see below). We estimated English mortality rates using the following data sources: age distributions for those with intermediate hyperglycaemia and diabetes, from combined HSE data (from 2009 to 2013); [8] mortality rates by age, from the Office of National Statistics (for 2015); [9] hazard ratios, for those with diabetes (1.32) and T2D (1.28) with reference to those without diabetes, from the National Diabetes Audit (for 2015-16). [10] We estimated mortality rates for those with intermediate hyperglycaemia using hazard ratios with reference to those with normoglycaemia as estimated (with 95% confidence intervals) by a systematic review and meta-analysis:[11] for IGT 1.32 (1.23 to 1.40) and for HbA1c 0.97 (0.88 to 1.07). We used 1.32 for IGT, but 1 for HbA1c because the estimate of 0.97 is not significantly different from 1. We

For peer review only - http://bmjopen.bmj.com/site/about/guidelines.xhtml

estimated mortality rates as follows, for 2015, for the English population: for normoglycaemia, 0.6% (compared with 1.07% for the general adult population); for intermediate hyperglycaemia, 1.9% and 2.3% for HbA1c and IGT; and for T2D, 2.3% and 2.2% for HbA1c and IGT. The probability of remaining in a state was derived as the residual (so all transition probabilities from each state sum to one).

In making future projections of the prevalence of T2D in England, without a preventive intervention, up to 2035, we used PHE estimates for 2015 of those with diabetes[12] and intermediate hyperglycaemia,[13] and derived the estimate of those with normoglycaemia as the residual for the population of England.[14] Given doubts over the reliability of diagnosing intermediate hyperglycaemia (IH),[15] we examined the robustness of our results by using the PHE estimate (IH = 5.05 million), and the extreme value of zero (IH = 0). The data sources of our estimates for England, of the prevalence of diabetes, intermediate hyperglycaemia and normoglycaemia; and of mortality rates of those with T2D, intermediate hyperglycaemia and normoglycaemia are given in the text.

Figure 3: Our Markov chain model to go about here

Estimating the trend in diagnosed diabetes

We estimated, by OLS regression analysis (using R),[16] the trend increase in the reported prevalence of diabetes as diagnosed by general practitioners in England, in the Quality Outcomes Framework (QOF) from 2004-05 (2004) to 2017-18 (2017)).[17] We used these estimates to give projections of the future prevalence of diagnosed diabetes to 2035.

Comparing projections of the prevalence of diabetes

We compared three sets of projections of the prevalence of diabetes and T2D in England from:

- different prevalence-based models,
- the trend in QOF data,

For peer review only - http://bmjopen.bmj.com/site/about/guidelines.xhtml

- our Markov chain models.

The ratios we used for making comparisons across different estimates and their sources are as follows:

- 75% for the ratio of diagnosed to the true prevalence of diabetes; [18][19]
- 90% for the ratio of the prevalence of T2D to diabetes; [12]
- 128% and 129% for the ratios, for 2030 and 2035, of the prevalence of diabetics in England aged over 15 to those aged 20 to 79 (England) in 2030 & 2035. [8], [9]

Patients and public involvement

Patients and the public were not involved in this research study.

Results

Rapid review 1: Methods of prevalence-based models

Rapid review 1 of methods of prevalence-based models retrieved 633 articles and from their citations we identified a further five by snowballing[20]. After removing duplicates, we screened 597 articles, of which 11 were relevant and fully assessed. After reviewing the full articles, five were excluded and seven were included in our analysis[5,21–26]. This review identified four different underlying models described in Table 1 which have been used to give five different projections of the future prevalence of diabetes for England and the UK. Two models produce global estimates: Shaw et al,[21] Guariguata et al[27], which is used by Whiting et al[22] and Guariguata et al;[23] and two for England only, the PHE model,[5] and the Association of Public Health Observatories (APHO) Diabetes Prevalence Model,[25] which is used by Hex et al[24] and Gatineau et al.[26]

For peer review only - http://bmjopen.bmj.com/site/about/guidelines.xhtml

Table 1: Methods of prevalence-based models

Model	Method of estimation	Prevalence rates used for projections	Validation against QOF data?	Model validation?	Confidence intervals?
Shaw et al[21]	Logistic regression	Age & sex	No	No	No
Guariguata et al[27]	Logistic regression	Age & sex, & urban / rural	No	No	No
Association of Public Health Observatories (APHO)[25,26]	Direct estimation from HSE for age, sex, & IMD. Trend in obesity estimated by linear regression.	Age & sex, Index of Multiple Deprivation (2004), Ethnicity & increases in obesity	Yes for 2008/09	No	Yes
PHE[5]	Logistic regression	Age & sex, ethnicity, IMD 2015	Yes for 2014/15	Yes: refitting model on 70% of data & assessing against remaining 30%	No

Each prevalence-based model uses: projected population changes; and estimates of the true age-specific prevalence rates of diabetes, from past annual Health Surveys for England (HSE), which are subject to two limitations. First, the small size of the sample means that the point estimate for the year of the survey is surrounded by large confidence interval estimates. Gatineau et al indicate that

For peer review only - http://bmjopen.bmj.com/site/about/guidelines.xhtml

the HSE survey for 2013 gives point estimate of prevalence of 7.3% with confidence interval estimates ranging from 4.3 to 10.3%.[26] The PHE model[5] reduces the sampling error from HSE by using three years of data (2012, 2013 and 2014). Second, the HSE estimates of prevalence are based on those who self-reported a diabetes diagnosis made by a doctor (by HbA1c or FPG); and, for those who have not been diagnosed and agreed to have a blood test, having a HbA1c value of 6.5% or more.[5] Hence these estimates may be in error because of poor reliability of self-reporting or because of actual diagnostic errors. Barry et al (p. 9) report that 'The most commonly used test (HbA1c) is neither sensitive nor specific; the fasting glucose test is specific but not sensitive'. [15] Holman et al (p.6) pointed out, however, that 'Although HbA1c and fasting identify different groups of people with undiagnosed diabetes, the proportion of people that are identified is similar'.[25]

Our review aimed to answer two questions about the models.

1. *How were the models validated?* A basic test of the validity of a forecasting model is to apply this to past data to predict a known future: e.g. does the model using HSE data from 2004 predict prevalence as estimated from HSE data in 2014? None of the accounts of the models we reviewed reports such a test. The PHE model[5] was validated by refitting the model on 70% of the data (randomly selected) and checking its estimates against the remaining 30% of data.
2. *Did the models try to take account of future changes in age-specific prevalence rates?* Only the APHO model[25] aimed to do this by estimating the net effect of trends in: changes in ethnicity; and being overweight and obese to create a sex-specific obesity adjustment index. They did not, however, give details of how that index was modelled. The other three models[5,21,27] assumed that future age-specific prevalence of diabetes would be as estimated from past HSEs.

The prevalence-based models we reviewed are focused on estimating geographical variations in the future prevalence of diabetes within countries, rather than giving sound estimates of future totals.

For peer review only - http://bmjopen.bmj.com/site/about/guidelines.xhtml

Rapid review 2: Markov chain models

Rapid review 2 of Markov models identified 304 articles. An additional one was snowballed. After removing duplicates, 222 articles were screened, 20 of them were considered relevant and fully assessed. Of these, one was excluded because we could not locate it, one did not report the results, and one modelled the progression from diabetes to its complications only. Table 2 gives details of the remaining 17 articles,[28–44]ordered in terms of their completeness of the information we could find on transition probabilities. (Appendix 2 gives additional information on objectives, model, population, outcomes, sensitivity analysis and validation.) Two articles did not report the measure of intermediate hyperglycaemia used.[40,44] Twelve reported a model using one risk measure only: nine models used IGT,[29,30,32–35,38,39,43] two HbA1c[37,41] and one FPG only.[28] Neumann et al reported two models, using IFG and IGT;[29] and Roberts et al[36], three models using HbA1c, IGT and IFG. Hence, we reviewed 20 models.

Our objective was to develop a matrix of transition probabilities, with one transition probability only between states, and hence designed to use available data for England. Table 2 gives the transition probabilities we found and shows no article provided the complete matrix of transition probabilities. Five only reported the full set between states other than death. No article reports transition probabilities from different states to death (i.e. mortality rates for each state) and, where relative risk of mortality is reported for intermediate hyperglycaemia and T2D, we could not always find whether this was compared with normoglycaemia. Nor could we find how these models satisfied the fundamental requirement of a Markov chain model that all transition probabilities out of a state, estimated from different datasets, (including return to that state) sum to one.

For peer review only - http://bmjopen.bmj.com/site/about/guidelines.xhtml

Table 2: Transition probabilities reported in different models for no preventive intervention (or standard care)

Reference	Measure of Intermediate Hyperglycaemia (IH)	Country	Normoglycaemia (NG) to IH	IH to NG	NG to T2D	T2D to NG	IH to T2D	T2D to IH	Mortality rates (Relative risk*)
Johansson et al, 2009 **[28]	FPG	Sweden							
Herman et al, 2005**[39]	IGT	USA					10.80%[45]		
Palmer et at, 2004[38]	IGT	Australia, France, Germany, Switzerland, & UK					Overall 11% for standard care Varies by age (10.8% to 11.6%) & Body Mass Index (9.0% to 14.3%) [46]		IH:1.37 (1.05 - 1.79) Undiagnosed T2D: 1.76 (1.17 – 2.66) Diagnosed T2D: 2.26 (1.78 – 2.87)
Zhuo et al, 2012**[41]	HbA1c	USA					0.07% to 18.9% by HbA1c [47]		
Chen et al, 2001[40]		Taiwan			1.10%[48]				
Zhou et al 2005[37]	HbA1c	USA			0%	0%		0%	
Schaufler & Wolfe 2010[42]	IGT or IFG	Germany	male, 2.23% female, 1.45%[49]		male, 2.51% & female, 1.66%[49]		male, 4.79% female, 4.23%[49]		Source given for higher mortality rates for T2D [50]
Gillies et al, 2008[30]	IGT	UK	< 65, 1.66% > 65, 2.49%[51]				1.96% based on 12 studies[51–62]		Increased risk of death with diabetes (hazard ratio) 0.756 (SE = 0.087) [63]

For peer review only - http://bmjopen.bmj.com/site/about/guidelines.xhtml

Reference	Measure of Intermediate Hyperglycaemia (IH)	Country	Normoglycaemia (NG) to IH	IH to NG	NG to T2D	T2D to NG	IH to T2D	T2D to IH	Mortality rates (Relative risk*)
									1% increase in HbA1c (hazard ratio) 0.104 (SE =0.039 [64]
Palmer & Tucker 2012 [31]	IGT	Australia		Reported over time for standard care [65] -10%, year 1 -5.6% year 2 - 3.5% years > 2	Reported for standard care 4.6% [51]	0%	Reported over time for standard care -11%, years 1 -3[46] -5.6%, years > 3[46]		IH: 1.50 (1.10–2.00) “undiagnosed” T2D: 1.30 (0.90–2.66) “diagnosed” T2D 2.30 (1.60–3.20) [66]
Ikeda et al, 2010[43]	IGT	Japan	3.10% [67]	For standard care 33.1%[68]	0%	0%	For standard care 6.6% [68]	0%	IH: 1.35 T2DM: 3.03 [69]
Smith et al, 2010[44]		USA	4% [70]		0.40% [71]	0%	10.80%[39]	0%	IH: 1.7 [72] stable T2D: 2 [73] complicated T2D: 2.4 [74]
Neumann et al, 2017[29]	IGT	Sweden	Risk equation reported	Risk equation reported	0%	0%	Risk equation reported	Risk equation reported	No increased risk for intermediate hyperglycaemia. T2D mortality not reported.
Caro et al, 2004[32]	IGT	Canada	16.30% (original estimate)	16.20% (original	0%	0%	6.30% (original estimate)	0%	IH: 1.45 (original estimate)

For peer review only - http://bmjopen.bmj.com/site/about/guidelines.xhtml

Reference	Measure of Intermediate Hyperglycaemia (IH)	Country	Normoglycaemia (NG) to IH	IH to NG	NG to T2D	T2D to NG	IH to T2D	T2D to IH	Mortality rates (Relative risk*)
				estimate)					
Neumann et al, 2011[33]	IGT	Germany	16.30% [32]	16.20% [32]	0%	0%	6.00%[75]	0.50% (original estimate)	
Liu et al, 2013[34]	IGT	China	1.28% [76]	11.60% [77]	0%	0%	initiation ages 25: 0.644% 40: 16.7% 60: 57.8% [78–80]	0%	
Wong et al, 2016[35]	IGT	Hong Kong	16.30% [32]	16.20% [32]	0%	0%	For usual practice years 1-3 11% [54] years > 4 5.6% [46]	0%	IH: 1.50 (1.10–2.00) T2D: 2.30 (1.60–3.20) [31]
Roberts et al, 2018[36]	IGT	England	6.33% [51]	8.97% [81]	0%	0%	4.55%[82]	0%	IH: 1.50 T2D: 1.9 [83]
	HbA1c	England	6.86% [51]	8.97% [81]	0%	0%	3.55%[82]	0%	IH: 1.2 T2D: 1.6 [83]
	IFG (ADA)	England	6.86% [51]	8.97% [81]	0%	0%	4.74%[82]	0%	IH: 1.2 T2D: 1.6 [83]
Range (for single probabilities)	IGT		1.28 -16.30%	8.97-16.20% (& for standard care from 3.5% to 33.1%)	0.00-2.5% (male) (& 4.6% for standard care)	0%	1.96-10.8% (& 11% for standard care)	0.00-0.5%	IH:1.35-1.7 T2D: 1.76-3.03
Meta-analyses	IGT						4.55%[82]		IH: 1.32 (1.23 to 1.40) [11]

For peer review only - http://bmjopen.bmj.com/site/about/guidelines.xhtml

Reference	Measure of Intermediate Hyperglycaemia (IH)	Country	Normoglycaemia (NG) to IH	IH to NG	NG to T2D	T2D to NG	IH to T2D	T2D to IH	Mortality rates (Relative risk*)
	HbA1c						3.55%[82]		IH: 0.97 (0.88 to 1.07) [11]
	IFG (ADA)						3.54%[82]		IH: 1.13, (1.02 - 1.25) [11]

Key and Notes:

0%: not allowed

*Relative risk over normoglycaemia specified in [29], [30], [32] [35], [30], [43]. Ranges in parentheses are 95% confidence intervals

** Models described elsewhere

For peer review only - http://bmjopen.bmj.com/site/about/guidelines.xhtml

Our review aimed to answer three questions about the Markov chain models:

1. *Do these articles provide evidence of the likely impact of national preventive programmes?* The primary focus of the articles we reviewed is on estimating the ratio of costs to benefits of preventive interventions for those who are hyperglycaemic (most based on IGT, only three for HbA1c, two for IFG, and one for FPG). None reported the impact of preventive interventions on reducing the burden of disease from T2D in the general population. Only four articles [19,26,32,41] modelled the general population (with normoglycaemia and intermediate hyperglycaemia)

2. *How were the models validated?* Whereas most articles reported outcomes of sensitivity analyses, only five reported comparisons of their models' outputs with other empirical data: clinical trials; [23, 33] the population with T2D in southern Wisconsin; [37] the disease progression of T2D in Germany; [34] mortality data for England and estimates of current prevalence of T2D by age group. [28] A good empirical test of a model's validity is of its estimates of those developing T2D in the absence of a preventive intervention. Only Caro et al [32] reported this for a general population, but they did not report a check against other projections. Of the articles that modelled populations with intermediate hyperglycaemia, only three reported estimates of the percentages developing T2D in the absence of intervention. [15,23,24]

3. *How do transition probabilities compare?* All models, except that of Neumann et al,[33] allow transitions from T2D to death only. Neumann et al[33] allow transition (at a low probability, 0.5%) from T2D to intermediate hyperglycaemia (IGT) (because 'this transition exists but seldom occurs', p 4). Only two models allow transition from normoglycaemia directly to T2D: Schaufler and Wolfe[42] (IFG or IGT - for males, 2.51% and females, 1.66%) and Smith et al (measure of intermediate hyperglycaemia not specified, 0.40%).[44] For the transition probabilities reported in Table 2,

For peer review only - http://bmjopen.bmj.com/site/about/guidelines.xhtml

two models allow for changes over time [23, 27]; and seven for variations by age [20, 21, 22, 23, 26, 30, 31]. Table 2 shows that wide ranges of transition probabilities used by the different IGT models: from normoglycaemia to intermediate hyperglycaemia, 1.28 to 16.30%; from intermediate hyperglycaemia to low, 8.97-16.20%; normoglycaemia to T2D, 0.00-2.51% (for males); intermediate hyperglycaemia to T2D, 1.96-10.8%. A meta-analysis recommended a rate of 4.55% for the last.[82]

The relative risks reported for intermediate hyperglycaemia for IGT ranged from 1.35 to 1.7; and T2D from 1.76 to 3.03. Roberts et al[36] report these risks for HbA1c to be 1.2 and 1.6. The estimates from the systematic review and meta-analysis[11] for intermediate hyperglycaemia were: for IGT 1.32 (1.23 to 1.40) and for HbA1c 0.97 (0.88 to 1.07). One article[33] reported a matrix in which probabilities of transitions between states other than death sum to one, which implies no one dies.

As PHE identify those with intermediate hyperglycaemia using HbA1c, [5] the model used by Roberts et al[36] for HbA1c is most appropriate for projecting the prevalence of T2D in England. They used the recommended transition probabilities from different risk measures of intermediate hyperglycaemia to T2D identified by a meta-analysis.[82] Neumann et al[33] and Caro et al[32] have similar transition probabilities, which are higher than those of Roberts et al, [36] for IGT from normoglycaemia to intermediate hyperglycaemia, and intermediate hyperglycaemia to T2D: 16.3% and 6.00% compared with 6.33% and 4.55%. We used the transition probabilities used by Neumann et al[33] because that is more recent. Model 1 is based on Roberts et al (HbA1c),[36] which was modified as Model 2 to give the projections of PHE. To do this, Model 2's transition probability from intermediate hyperglycaemia to T2D (0.013) is a third of that of Model 1 (0.036), and below the lowest rate of any model we reviewed (0.02). (Model 2 has a corresponding increase in the transition probability of remaining in intermediate hyperglycaemia (0.836 to 0.878).)

For peer review only - http://bmjopen.bmj.com/site/about/guidelines.xhtml

Model 3 is based on Neumann et al.[33] Details of the models are given in Appendix 1.

Estimating the trend in diagnosed diabetes

Table 3 reports the OLS estimate of the trend in diagnosed diabetes from QOF data,[17] which gives an annual rate of increase of 11%.

Table 3: The trend model from QOF data

Coefficients	Value	Standard error	T	Pr > |t|	Lower bound (95%)	Upper bound (95%)
Intercept	-229	2.436	94.228	< 0.0001	-234.889	-224.167
Year	0.115	0.001	95.235	< 0.0001	0.113	0.118
Adjusted R-squared	0.9987					

Comparing projections of the future prevalence of T2D

Table 4 gives: for the different prevalence-based models their defined populations, data sources, and projections of diabetes true prevalence (in millions); comparable estimates of the true prevalence of diabetes from the QOF trend (increased by a third); and the annual rate of increase in prevalence from the first in the series to the last. Table 4 shows that, for the three models that do not allow for increase in prevalence rates by age and sex,[21–23] the older the HSE data used, the lower is the estimate of the rate of increase in prevalence for England. We compare projections of true prevalence of diabetes and T2D by different models giving numbers in millions; and, in parentheses, confidence intervals (where available).

For peer review only - http://bmjopen.bmj.com/site/about/guidelines.xhtml

Table 4: True diabetes prevalence (millions) estimated by different epidemiological models and from the QOF Trend

Source of estimate	Population	Data source	Details of series									
			First year	Prevalence	Final Year	Prevalence	Annual rate of increase (%)*	Projections				
								2015	2020	2025	2030	2035
Shaw et al[21]	UK: 20 to 79 (UN, 2007)	HSE (2003)	2010	2.14	2030	2.55	2.0				2.55	
Whiting et al[22]	UK: 20 to 79 (UN, 2011)	HSE (2004 & 2009)	2011	3.06	2030	3.65	3.1				3.65	
Guariguata et al[23]	UK: 20 to 79 (UN, 2011)	HSE (2004)	2013	2.98	2035	3.62	2.9					3.62
Holman et al[25]	England: >15 (ONS)	HSE (2006)	2010	3.10	2030	4.60 (3.25-6.88)	7.5	3.47 (2.47-5.07)	3.82 (2.70-5.62)	4.19 (2.93-6.19)	4.60 (3.25-6.88)	
PHE[5]	England: >15 (ONS)	HSE (2012, 2013 & 2014)	2015	3.81	2035	4.94	5.6	3.81	4.09	4.39	4.68	4.94
QOF trend**	England: >15 registered with GPs	QOF (2004-05 to 2017-18)	2004-05	1.77	2017	3.20	11.0	3.99 (3.88-4.09)	4.72 (4.61-4.84)	5.46 (5.32-5.59)	6.19 (6.04-6.35)	6.93 (6.75-7.11)

Notes:

*Estimated as the rate of increase from the first estimate to the last

** To estimate the true prevalence from the QOF trend these estimates were increased by a third.

For peer review only - http://bmjopen.bmj.com/site/about/guidelines.xhtml

Global models give three projections of the true prevalence for diabetes prevalence in the UK (aged 20 to 79): for 2030, 2.55[21] and 3.65;[22] and 2035, 3.62.[23] Each projection is below the estimate by PHE[12] for England for 2015, 3.81 (based on HSEs for 2012, 2103 and 2014). These global models assume low rates of increase in prevalence over time and exclude those over 79, who we estimated to account for nearly 30% who would be over 15 in England and develop diabetes in 2030 and 2035. The projections by these global models are not examined further.

Two models give projections of the true prevalence of diabetes for England only (aged over 15): the PHE model[12] for 2030, 4.68 and 2035, 4.94; and APHO for 2030, 4.60 (3.25 to 6.88).[25] The two accounts of the APHO model [15, 16] report the same projection for 2030; but one estimated the prevalence of diabetes in 2010 (3.10) [25] to be higher than the other for 2013 (2.17). [16] And the increase in prevalence to 2030 attributed to increases in obesity, was estimated to be a half [25] and a third.[26] Figure 4 compares three projections for 2025: PHE,[12] 4.39; Holman et al. 4.19 (2.93-6.19); and the QOF trend, 5.46 (5.32-5.59), which has a narrow confidence interval because this trend has been so stable.

Figure 4: Projections of true diabetes prevalence in England: 2005 to 2035 to go about here

Figure 5 compares projections of the true prevalence of T2D in England to 2035 from PHE, the QOF trend, and our three Markov chain models. This shows that the projections by Model 2 replicated the projections by PHE; by Model 1 are above those from PHE and the QOF trend; by Model 3 seem to be implausibly explosive. Figure 5 also shows the impact of reducing the estimate of those with intermediate hyperglycaemia to zero in 2015 on the projections by models 1 and 3. Table 5 gives projections for 2025, These are: 3.95, by PHE; 4.91 (4.79 to 5.03) from the QOF trend; 5.64 (5.12 to 10.3) by Model 1; 3.86 (2.06 to 4.27) by Model 2; and 9.10 (8.84 to 18.8) by Model 3. Putting the estimate of those with

For peer review only - http://bmjopen.bmj.com/site/about/guidelines.xhtml

intermediate hyperglycaemia to zero in 2015 reduces the projections by Models 1 and 3 to 5.05 and 8.10, which are above the projections by PHE and the QOF trend.

Table 5: Projections of the true prevalence of T2D in England for 2025

Model	Projections for 2025 (millions)			
	Statistical		Markov (numbers with intermediate hyperglycaemia in 2015)	
	Point estimate	95% confidence intervals	5.05*	Zero
PHE	3.95	n.a.		
QOF trend;	4.91	4.79 to 5.03		
Model 1**			5.64	5.05
Model 2 ***			3.86	
Model 3****			9.07	8.57

Notes
n.a Not available
* as estimated by PHE
**based on Roberts et al,[36]
***based on Roberts et al,[36] but modified to reproduce the QOF trend to 2035
**** based on Neuman et al[33]

Figure 5: Projections of the true prevalence of T2D in England: 2015 to 2035 to go about here

Discussion

Akushevich et al,[84] point out that although the 'prevalence probability of a disease is a fundamental epidemiologic characteristic' for which there are various data sources, this random variable is the difference between changes over time in disease incidence and patient survival. This has a statistical implication that, whatever modelling approach is used, we would expect projections of prevalence to have large errors of estimation. The policy implication, which Akushevich et al emphasise, is that the overriding objective ought to be to improve population health, rather than reducing the prevalence of T2D: because, e.g., improving survival of those with T2D, may increase prevalence (depending on changes in incidence). Akushevich et al developed a new methodological approach that partitions trends in observed disease prevalence into their two components, and hence gives estimates of the direction

For peer review only - http://bmjopen.bmj.com/site/about/guidelines.xhtml

and strength of the effect of each. Their models are estimated from a single data set (Medicare data), incorporate changes over time and take account of age.

The four prevalence-based models we reviewed[10,14,15,17] use past estimated prevalence rates by age and sex and projected changes in populations. They are focused on estimating geographical variations in the future prevalence of diabetes within countries, rather than giving sound estimates of future totals. Only one model aims to take account of changes in prevalence rates by age and sex over time.[15] Of the five projections of diabetes prevalence, for England and the UK we reviewed,[10–12,14,15] only one[15] reported confidence intervals.

The Markov chain models of the economic impacts of interventions that aim to prevent T2D, which we reviewed, aim to capture changes in incidence and survival in one model. Their primary focus is on estimating the ratio of costs to benefits of preventive interventions for those who are hyperglycaemic (mostly based on IGT). None reported the impact of preventive interventions on reducing the burden of disease from T2D in the general population. We could not find a complete matrix of transition probabilities; nor descriptions of how transition probabilities estimated from different datasets satisfied the fundamental requirement of a Markov chain model that all transition probabilities out of a state sum to one. The transition probabilities we did find do not vary over time. In seven articles these probabilities do vary by age [20 21, 22, 23, 26, 30, 31]. In their systematic review of models of the economic impacts of preventive interventions, Leal et al [7] also found the majority of models assumed that 'the rate of progression to T2D was constant across the entire prediabetes population'. They attribute this in part to limitations in the available data, but highlight the 'stark contrast' between these simple models and 'The complexity of risk prediction models for diabetes incidence and the variety of covariates used'. [85],[86] Friedman famously[87] argued, however, that the relevant question to ask about the 'assumptions' of economic theory, 'is not whether they are descriptively realistic … but whether the theory works, which means that it yields sufficiently accurate predictions' [p 153].

For peer review only - http://bmjopen.bmj.com/site/about/guidelines.xhtml

Three projections of diabetes prevalence (in millions) for the UK (aged 20 to 79) by global models are: for 2030, 2.55 [21,22] and 3.65; [21,22], and for 2035, 3.62. [23] Each is below the PHE estimate of 3.81 for 2015 for England only (over 15) [12]. This raises questions over the validity of these global projections; and their excision of those over 79, who we estimated to account for nearly 30% of developing T2D after 2030. We report three estimates of diabetes prevalence in England for 2025 (with 95% confidence intervals where available): by 4.39 by PHE, [12] 4.19 (2.93 to 6.19) by the APHO model, [25,26] and from the QOF trend, 5.46, (5.32-5.59). We, and Leal et al, [7] found only minority of articles reported tests of validation. Such checks are vital for Markov chain models given the different data sources used to estimate transition probabilities.

Our Markov Chain models are based on transition probabilities to states other than death from published models, to death from English mortality rates, and of remaining in a state as the residual (so all transition probabilities from each state sum to one). The projections of prevalence of T2D for England for 2025 are: 5.64 by Model 1 (based on Roberts et al for HbA1c),[36] and 9.1 by Model 3 (based on Neuman et al (for IGT)[33]. To reproduce PHE's projections by Model 2, of 3.86, Model 1 was modified with a lower probability of transition from intermediate hyperglycaemia to T2D than any of the models we reviewed. These comparisons suggest that the PHE projection of T2D prevalence in 2025 of 4 million is too low, and a more realistic estimate is about 5 million.

The limitations of our research are our models are simple and transparent, and, as we did not undertake systematic reviews, we may have omitted relevant articles. The systematic review by Leal et al [7] reviewed 29 studies, which included 12 of the 17 studies of Markov chain models that we reviewed. Their principal findings are strikingly similar to ours. They recommend the development of 'more comprehensive models that are capable of better capturing the continuity in disease progression and, also, of incorporating the identification of novel biomarkers'. But, they recognise such models require more detailed data and only need to be comprehensive enough to provide reliable estimates for decision making.

For peer review only - http://bmjopen.bmj.com/site/about/guidelines.xhtml

Conclusions

There are three implications of our reviews of two types of models used to project prevalence of T2D. First, current prevalence-based models are focused on estimating geographical variations in the future prevalence of diabetes within countries, rather than giving sound estimates of future totals. They are designed to underestimate the scale of increases in the future prevalence of T2D in England and the UK, and hence the urgency for governments to implement preventive interventions. Second, the primary focus of the Markov chain models is on estimating the ratio of costs to benefits of preventive interventions for those who are hyperglycaemic (mostly based on IGT). We found that no articles gave the complete matrix of transition probabilities and a full description of how they were derived. Only a minority have been subjected to tests of validity. Third, to inform national policies, governments need estimates of the impacts of preventive interventions on reducing the burden of disease from T2D in the general population. These estimates ought to be derived from validated models, designed to use available data, that estimate changes over time in the incidence and survival of patients with T2D, with and without preventive interventions.

Acknowledgments

We are grateful to our two referees, Professor Anders Green and Professor Igor Akushevich, for critical comments on earlier drafts. Their comments have clarified our argument and helped us to consider the adequacy of two types of models that project changes in prevalence, given that this is the observed outcome of changes in incidence and survival.

Figure legends

Figure 1 - Review flowchart of epidemiological models

For peer review only - http://bmjopen.bmj.com/site/about/guidelines.xhtml

Figure 2: Review flowcharts of Markov chain models

Figure 3: Our Markov chain model

Figure 4: Projections of true diabetes prevalence in England: 2005 to 2035

Figure 5: Projections of the true prevalence of T2D in England: 2015 to 2035

Author contributions

Mi Jun Keng did the original work in developing initial Markov Chain models to estimate the impacts of preventive interventions on the future prevalence of Type 2 Diabetes (T2D) in England and has been involved throughout this project. Chiara De Poli worked with Mi Jun in developing those models, undertook the rapid reviews of epidemiological and Markov Chain models, and commented on drafts of this paper. Gwyn Bevan prepared drafts of the paper, reviewed epidemiological and Markov Chain models, developed the models used in this paper, and undertook comparisons of projections. Rosalind Raine took part in the workshops on the models, reviewed our methods and findings, and commented on drafts.

Declaration of interests

There are no conflicts of interest.

Funding

Funding was from the National Institute for Health Research (NIHR) Collaboration for Leadership in Applied Health Research and Care North Thames at Barts Health NHS Trust, which had no role in the writing of the manuscript or the decision to submit it for publication. None of us has been paid to write this

For peer review only - http://bmjopen.bmj.com/site/about/guidelines.xhtml

article. The corresponding author has had full access to all the data in the study and had final responsibility for the decision to submit for publication.

Data statement

The data we have used are from cited public sources.

Ethics approval

No ethics approval was sought for this study.

For peer review only

References

1 NCD Risk Factor Collaboration (NCD-RisC). Worldwide trends in diabetes since 1980: a pooled analysis of 751 population-based studies with 4.4 million participants. *Lancet (London, England)* 2016;**387**:1513–30. doi:10.1016/S0140-6736(16)00618-8

2 NHS DPP Programme Support Team. NHS Diabetes Prevention Programme (NHS DPP) - Primary Care Toolkit To Support Local Implementation of the NHS DPP. London (UK):

3 Public Health England, NHS England. National NHS Diabetes initiative launched in major bid to prevent illness (press release). 2015.

4 De Poli C, Bevan G, Keng MJ, *et al.* Impact of interventions to prevent diabetes in England: a simulation model. *Lancet* 2017;**390**:S36. doi:10.1016/S0140-6736(17)32971-9

5 Public Health England. Technical document for the diabetes prevalence model for England 2016. London: 2016.

6 Khangura S, Konnyu K, Cushman R, *et al.* Evidence summaries: The evolution of a rapid review approach. *Syst Rev* Published Online First: 2012. doi:10.1186/2046-4053-1-10

7 Leal J, Morrow LM, Khurshid W, *et al.* Decision models of prediabetes populations: A systematic review. *Diabetes, Obes Metab* Published Online First: 2019. doi:10.1111/dom.13684

8 National Cardiovascular Intelligence Network (NCVIN)- Public Health England. NHS Diabetes Prevention Programme (NHS DPP) Non-diabetic hyperglycaemia. 2015.

9 Office for National Statistics. 2014-based National Population Projections. 2015.

10 Healthcare Quality Improvement Partnership (HQIP). National Diabetes Audit, 2015-16 Report 2: Complications and Mortality. 2017.

11 Huang Y, Cai X, Mai W, *et al.* Association between prediabetes and risk of cardiovascular disease and all cause mortality: systematic review and meta-

For peer review only - http://bmjopen.bmj.com/site/about/guidelines.xhtml

analysis. *BMJ* 2016;**355**:i5953. doi:10.1136/bmj.i5953

12 National Cardiovascular Intelligence Network (NCVIN) - Public Health England. Prevalence estimates of diabetes. 2016.

13 Public Health England - National Cardiovascular Intelligence Network. Prevalence estimates of non-diabetic hyperglycaemia. 2015.

14 Office for National Statistics. National Population Projections: 2014-based Statistical Bulletin. *Off Natl Stat* Published Online First: 2015. doi:10.1038/nature09689

15 Barry E, Roberts S, Oke J, *et al.* Efficacy and effectiveness of screen and treat policies in prevention of type 2 diabetes: systematic review and meta-analysis of screening tests and interventions. *BMJ* 2017;**356356**. doi:10.1136/bmj.i6538

16 RStudio Team. RStudio: Integrated Development for R. 2015.

17 NHS Digital. Quality and Outcomes Framework (QOF) - 2016-17. 2017.

18 Holman N, Forouhi NG, Goyder E, *et al.* The Association of Public Health Observatories (APHO) Diabetes Prevalence Model: Estimates of total diabetes prevalence for England, 2010-2030. *Diabet Med* 2011;**28**:575–82. doi:10.1111/j.1464-5491.2010.03216.x

19 Moody A, Cowley G, Fat LN, *et al.* Social inequalities in prevalence of diagnosed and undiagnosed diabetes and impaired glucose regulation in participants in the Health Surveys for England series. *BMJ Open* 2016;**6**:e010155. doi:10.1136/BMJOPEN-2015-010155

20 Wohlin C. Guidelines for snowballing in systematic literature studies and a replication in software engineering. In: *ACM International Conference Proceeding Series*. 2014. doi:10.1145/2601248.2601268

21 Shaw JEE, Sicree RAA, Zimmet PZZ. Global estimates of the prevalence of diabetes for 2010 and 2030. *Diabetes Res Clin Pract* 2010;**87**:4–14. doi:10.1016/j.diabres.2009.10.007

22 Whiting DR, Guariguata L, Weil C, *et al.* IDF diabetes atlas: global estimates of the prevalence of diabetes for 2011 and 2030. *Diabetes Res Clin Pract* 2011;**94**:311–

For peer review only - http://bmjopen.bmj.com/site/about/guidelines.xhtml

21. doi:10.1016/j.diabres.2011.10.029

23 Guariguata L, Whiting DR, Hambleton I, *et al.* Global estimates of diabetes prevalence for 2013 and projections for 2035. *Diabetes Res Clin Pract* 2014;**103**:137–49. doi:10.1016/j.diabres.2013.11.002

24 Hex N, Bartlett C, Wright D, *et al.* Estimating the current and future costs of Type 1 and Type 2 diabetes in the UK, including direct health costs and indirect societal and productivity costs. *Diabet Med* 2012;**29**:855–62. doi:10.1111/j.1464-5491.2012.03698.x

25 Holman N, Forouhi NG, Goyder E, *et al.* The Association of Public Health Observatories (APHO) Diabetes Prevalence Model: estimates of total diabetes prevalence for England, 2010-2030. *Diabet Med* 2011;**28**:575–82. doi:10.1111/j.1464-5491.2010.03216.x

26 Gatineau M, Hancock C, Holman N, *et al.* Adult obesity and type 2 diabetes. London, UK: 2014.

27 Guariguata L, Whiting D, Weil C, *et al.* The International Diabetes Federation diabetes atlas methodology for estimating global and national prevalence of diabetes in adults. *Diabetes Res Clin Pract* 2011;**94**:322–32. doi:10.1016/j.diabres.2011.10.040

28 Johansson P, Östenson C-G, Hilding AM, *et al.* A cost-effectiveness analysis of a community-based diabetes prevention program in Sweden. *Int J Technol Assess Health Care* 2009;**25**:350–8. doi:10.1017/S0266462309990079

29 Neumann A, Lindholm L, Norberg M, *et al.* The cost-effectiveness of interventions targeting lifestyle change for the prevention of diabetes in a Swedish primary care and community based prevention program. *Eur J Heal Econ* 2017;**18**:905–19. doi:10.1007/s10198-016-0851-9

30 Gillies CL, Lambert PC, Abrams KR, *et al.* Different strategies for screening and prevention of type 2 diabetes in adults: cost effectiveness analysis. *BMJ* 2008;**336**.

31 Palmer AJ, Tucker DMD, Polkinghorne KR, *et al.* Cost and clinical implications of diabetes prevention in an Australian setting: A long-term modeling analysis. *Prim*

For peer review only - http://bmjopen.bmj.com/site/about/guidelines.xhtml

Care Diabetes 2012;**6**:109–21. doi:10.1016/j.pcd.2011.10.006

32 Caro JJ, Getsios D, Caro I, *et al.* Economic evaluation of therapeutic interventions to prevent Type 2 diabetes in Canada. *Diabet Med* 2004;**21**:1229–36. doi:10.1111/j.1464-5491.2004.01330.x

33 Neumann A, Schwarz P, Lindholm L. Estimating the cost-effectiveness of lifestyle intervention programmes to prevent diabetes based on an example from Germany: Markov modelling. *Cost Eff Resour Alloc* 2011;**9**:17. doi:10.1186/1478-7547-9-17

34 Liu X, Li C, Gong H, *et al.* An economic evaluation for prevention of diabetes mellitus in a developing country: a modelling study. *BMC Public Health* 2013;**13**:729. doi:10.1186/1471-2458-13-729

35 Wong CKH, Jiao F-F, Siu S-C, *et al.* Cost-Effectiveness of a Short Message Service Intervention to Prevent Type 2 Diabetes from Impaired Glucose Tolerance. *J Diabetes Res* 2016;**2016**:1–8. doi:10.1155/2016/1219581

36 Roberts S, Craig D, Adler A, *et al.* Economic evaluation of type 2 diabetes prevention programmes: Markov model of low- and high-intensity lifestyle programmes and metformin in participants with different categories of intermediate hyperglycaemia. *BMC Med* 2018;**16**:16. doi:10.1186/s12916-017-0984-4

37 Zhou H, Isaman DJM, Messinger S, *et al.* A computer simulation model of diabetes progression, quality of life, and cost. *Diabetes Care* 2005;**28**:2856–63.

38 Palmer AJ, Roze S, Valentine WJ, *et al.* Intensive lifestyle changes or metformin in patients with impaired glucose tolerance: Modeling the long-term health economic implications of the diabetes prevention program in Australia, France, Germany, Switzerland, and the United Kingdom. *Clin Ther* 2004;**26**:304–21. doi:10.1016/S0149-2918(04)90029-X

39 Herman WH, Hoerger TJ, Brandle M, *et al.* The cost-effectiveness of lifestyle modification or metformin in preventing type 2 diabetes in adults with impaired glucose tolerance. *Ann Intern Med* 2005;**142**:323–32.

For peer review only

40 Chen TH-H, Yen M-F, Tung T-H. A computer simulation model for cost–effectiveness analysis of mass screening for Type 2 diabetes mellitus. *Diabetes Res Clin Pract* 2001;**54**:37–42. doi:10.1016/S0168-8227(01)00307-2

41 Zhuo X, Zhang P, Selvin E, *et al.* Alternative HbA1c Cutoffs to Identify High-Risk Adults for Diabetes Prevention. *Am J Prev Med* 2012;**42**:374–81. doi:10.1016/j.amepre.2012.01.003

42 Schaufler TM, Wolff M. Cost Effectiveness of Preventive Screening Programmes for Type 2 Diabetes Mellitus in Germany. *Appl Health Econ Health Policy* 2010;**8**:191–202. doi:10.2165/11532880-000000000-00000

43 Ikeda S, Kobayashi M, Tajima N. Cost-effectiveness analysis of voglibose for prevention of type 2 diabetes mellitus in Japanese patients with impaired glucose tolerance. 2010;**1**:252–8. doi:10.1111/j.2040-1124.2010.00052.x

44 Smith KJ, Hsu HE, Roberts MS, *et al.* Cost-effectiveness analysis of efforts to reduce risk of type 2 diabetes and cardiovascular disease in southwestern Pennsylvania, 2005-2007. *Prev Chronic Dis* 2010;**7**:A109.

45 The CDC Diabetes Cost-effectiveness Group TCDC. Cost-effectiveness of Intensive Glycemic Control, Intensified Hypertension Control, and Serum Cholesterol Level Reduction for Type 2 Diabetes. *JAMA* 2002;**287**:2542. doi:10.1001/jama.287.19.2542

46 Diabetes Prevention Program Research Group, Knowler WC, Fowler SE, *et al.* 10-year follow-up of diabetes incidence and weight loss in the Diabetes Prevention Program Outcomes Study. *Lancet (London, England)* 2009;**374**:1677–86. doi:10.1016/S0140-6736(09)61457-4

47 Selvin E, Steffes MW, Zhu H, *et al.* Glycated Hemoglobin, Diabetes, and Cardiovascular Risk in Nondiabetic Adults. *N Engl J Med* 2010;**362**:800–11. doi:10.1056/NEJMoa0908359

48 Kuo HS, Chang HJ, Chou P, *et al.* A Markov chain model to assess the efficacy of screening for non-insulin dependent diabetes mellitus (NIDDM). *Int J Epidemiol* 1999;**28**:233–40.

For peer review only - http://bmjopen.bmj.com/site/about/guidelines.xhtml

49 Icks A, Rathmann W, Haastert B, *et al.* Cost-Effectiveness of Type 2 Diabetes Screening: Results from Recently Published Studies. *Das Gesundheitswes* 2005;**67**:167–71. doi:10.1055/s-2005-858232

50 Roper NA, Bilous RW, Kelly WF, *et al.* Cause-specific mortality in a population with diabetes: South Tees Diabetes Mortality Study. *Diabetes Care* 2002;**25**:43–8.

51 Meigs JB, Muller DC, Nathan DM, *et al.* The natural history of progression from normal glucose tolerance to type 2 diabetes in the Baltimore Longitudinal Study of Aging. *Diabetes* 2003;**52**:1475–84.

52 Bonora E, Kiechl S, Willeit J, *et al.* Population-based incidence rates and risk factors for type 2 diabetes in white individuals: the Bruneck study. *Diabetes* 2004;**53**:1782–9.

53 Wein P, Beischer N, Harris C, *et al.* A trial of simple versus intensified dietary modification for prevention of progression to diabetes mellitus in women with impaired glucose tolerance. *Aust N Z J Obstet Gynaecol* 1999;**39**:162–6.

54 Knowler WC, Barrett-Connor E, Fowler SE, *et al.* Reduction in the incidence of type 2 diabetes with lifestyle intervention or metformin. *N Engl J Med* 2002;**346**:393–403. doi:10.1056/NEJMoa012512

55 Chiasson J-L, Josse RG, Gomis R, *et al.* Acarbose for prevention of type 2 diabetes mellitus: the STOP-NIDDM randomised trial. *Lancet* 2002;**359**:2072–7. doi:10.1016/S0140-6736(02)08905-5

56 de Vegt F, Dekker JM, Jager A, *et al.* Relation of impaired fasting and postload glucose with incident type 2 diabetes in a Dutch population: The Hoorn Study. *JAMA* 2001;**285**:2109–13.

57 Edelstein SL, Knowler WC, Bain RP, *et al.* Predictors of progression from impaired glucose tolerance to NIDDM: an analysis of six prospective studies. *Diabetes* 1997;**46**:701–10.

58 Eriksson J, Lindström J, Valle T, *et al.* Prevention of Type II diabetes in subjects with impaired glucose tolerance: the Diabetes Prevention Study (DPS) in Finland. Study design and 1-year interim report on the feasibility of the lifestyle

For peer review only - http://bmjopen.bmj.com/site/about/guidelines.xhtml

intervention programme. *Diabetologia* 1999;**42**:793–801.

59 Eriksson J, Lehtovirta M, Ehrnstrom B, *et al.* Long-term beneficial effects of glipizide treatment on glucose tolerance in subjects with impaired glucose tolerance. *J Intern Med* 2006;**259**:553–60. doi:10.1111/j.1365-2796.2006.01633.x

60 Jarrett RJ, Keen H, Fuller JH, *et al.* Worsening to diabetes in men with impaired glucose tolerance ('borderline diabetes'). *Diabetologia* 1979;**16**:25–30.

61 Wareham NJ, Byrne CD, Williams R, *et al.* Fasting proinsulin concentrations predict the development of type 2 diabetes. *Diabetes Care* 1999;**22**:262–70.

62 Rasmussen SS, Glümer C, Sandbaek A, *et al.* Progression from impaired fasting glucose and impaired glucose tolerance to diabetes in a high-risk screening programme in general practice: the ADDITION Study, Denmark. *Diabetologia* 2007;**50**:293–7. doi:10.1007/s00125-006-0530-y

63 DECODE study group. Glucose tolerance and mortality: comparison of WHO and American Diabetic Association diagnostic criteria. *Lancet* 1999;**354**:617–21. doi:10.1016/S0140-6736(98)12131-1

64 Rossing P, Hougaard P, Borch-Johnsen K, *et al.* Predictors of mortality in insulin dependent diabetes: 10 year observational follow up study. *BMJ* 1996;**313**:779–84.

65 Perreault L, Kahn SE, Christophi CA, *et al.* Regression from pre-diabetes to normal glucose regulation in the diabetes prevention program. *Diabetes Care* 2009;**32**:1583–8. doi:10.2337/dc09-0523

66 Barr ELM, Magliano DJ, Polkinghorne KR, *et al.* The Australian Diabetes, Obesity and Lifestyle Study. 2006.

67 Oizumi T. [Epidemiologic investigation on the incidence of diabetes mellitus from impaired glucose tolerance in population based study]. *Nihon Rinsho* 2005;**63 Suppl 2**:73–7.

68 Kawamori R, Tajima N, Iwamoto Y, *et al.* Voglibose for prevention of type 2 diabetes mellitus: a randomised, double-blind trial in Japanese individuals with

For peer review only

For peer review only - http://bmjopen.bmj.com/site/about/guidelines.xhtml

impaired glucose tolerance. *Lancet* Published Online First: 2009. doi:10.1016/S0140-6736(09)60222-1

69 Nakagami T, DECODA Study Group. Hyperglycaemia and mortality from all causes and from cardiovascular disease in five populations of Asian origin. *Diabetologia* 2004;**47**:385–94. doi:10.1007/s00125-004-1334-6

70 Orchard TJ, Temprosa M, Goldberg R, *et al.* The effect of metformin and intensive lifestyle intervention on the metabolic syndrome: the Diabetes Prevention Program randomized trial. *Ann Intern Med* 2005;**142**:611–9.

71 Fox CS, Pencina MJ, Meigs JB, *et al.* Trends in the Incidence of Type 2 Diabetes Mellitus From the 1970s to the 1990s: The Framingham Heart Study. *Circulation* 2006;**113**:2914–8. doi:10.1161/CIRCULATIONAHA.106.613828

72 Lakka H-M, Laaksonen DE, Lakka TA, *et al.* The metabolic syndrome and total and cardiovascular disease mortality in middle-aged men. *JAMA* 2002;**288**:2709–16.

73 Moss SE, Klein R, Klein BE. Cause-specific mortality in a population-based study of diabetes. *Am J Public Health* 1991;**81**:1158–62.

74 Fuller JH, Stevens LK, Wang SL. Risk factors for cardiovascular mortality and morbidity: the WHO Mutinational Study of Vascular Disease in Diabetes. *Diabetologia* 2001;**44 Suppl 2**:S54-64.

75 Tuomilehto J, Lindström J, Eriksson JG, *et al.* Prevention of Type 2 Diabetes Mellitus by Changes in Lifestyle among Subjects with Impaired Glucose Tolerance. *N Engl J Med* 2001;**344**:1343–50. doi:10.1056/NEJM200105033441801

76 Pan XR, Hu YH, Li GW, *et al.* Impaired glucose tolerance and its relationship to ECG-indicated coronary heart disease and risk factors among Chinese. Da Qing IGT and diabetes study. *Diabetes Care* 1993;**16**:150–6.

77 Harris MI, Klein R, Welborn TA, *et al.* Onset of NIDDM occurs at least 4-7 yr before clinical diagnosis. *Diabetes Care* 1992;**15**:815–9.

78 Pan X-R, Li G-W, Hu Y-H, *et al.* Effects of Diet and Exercise in Preventing NIDDM in People With Impaired Glucose Tolerance: The Da Qing IGT and Diabetes Study. *Diabetes Care* 1997;**20**.

For peer review only

79 Jia WP, Pang C, Chen L, *et al.* Epidemiological characteristics of diabetes mellitus and impaired glucose regulation in a Chinese adult population: the Shanghai Diabetes Studies, a cross-sectional 3-year follow-up study in Shanghai urban communities. *Diabetologia* 2007;**50**:286–92. doi:10.1007/s00125-006-0503-1

80 Dunstan DW, Zimmet PZ, Welborn TA, *et al.* The rising prevalence of diabetes and impaired glucose tolerance: the Australian Diabetes, Obesity and Lifestyle Study. *Diabetes Care* 2002;**25**:829–34.

81 Balk EM, Earley A, Raman G, *et al.* Combined Diet and Physical Activity Promotion Programs to Prevent Type 2 Diabetes Among Persons at Increased Risk: A Systematic Review for the Community Preventive Services Task Force. *Ann Intern Med* 2015;**163**:437–51. doi:10.7326/M15-0452

82 Morris DH, Khunti K, Achana F, *et al.* Progression rates from HbA1c 6.0–6.4% and other prediabetes definitions to type 2 diabetes: a meta-analysis. *Diabetologia* 2013;**56**:1489–93. doi:10.1007/s00125-013-2902-4

83 DECODE Study Group - European Diabetes Epidemiology. Is the current definition for diabetes relevant to mortality risk from all causes and cardiovascular and noncardiovascular diseases? *Diabetes Care* 2003;**26**:688–96.

84 Akushevich I, Yashkin AP, Kravchenko J, *et al.* Identifying the causes of the changes in the prevalence patterns of diabetes in older U.S. adults: A new trend partitioning approach. *J Diabetes Complications* Published Online First: 2018. doi:10.1016/j.jdiacomp.2017.12.014

85 Collins GS, Mallett S, Omar O, *et al.* Developing risk prediction models for type 2 diabetes: A systematic review of methodology and reporting. *BMC Med* Published Online First: 2011. doi:10.1186/1741-7015-9-103

86 Hippisley-Cox J, Coupland C. Development and validation of QDiabetes-2018 risk prediction algorithm to estimate future risk of type 2 diabetes: cohort study. *BMJ* Published Online First: 2017. doi:10.1136/bmj.j5019

87 Friedman M. The methodology of positive economics. In: *The Philosophy of Economics: An Anthology*. 2007. doi:10.1017/CBO9780511819025.010

For peer review only - http://bmjopen.bmj.com/site/about/guidelines.xhtml

For peer review only

For peer review only - http://bmjopen.bmj.com/site/about/guidelines.xhtml

Figure 1 - Review flowchart of epidemiological models

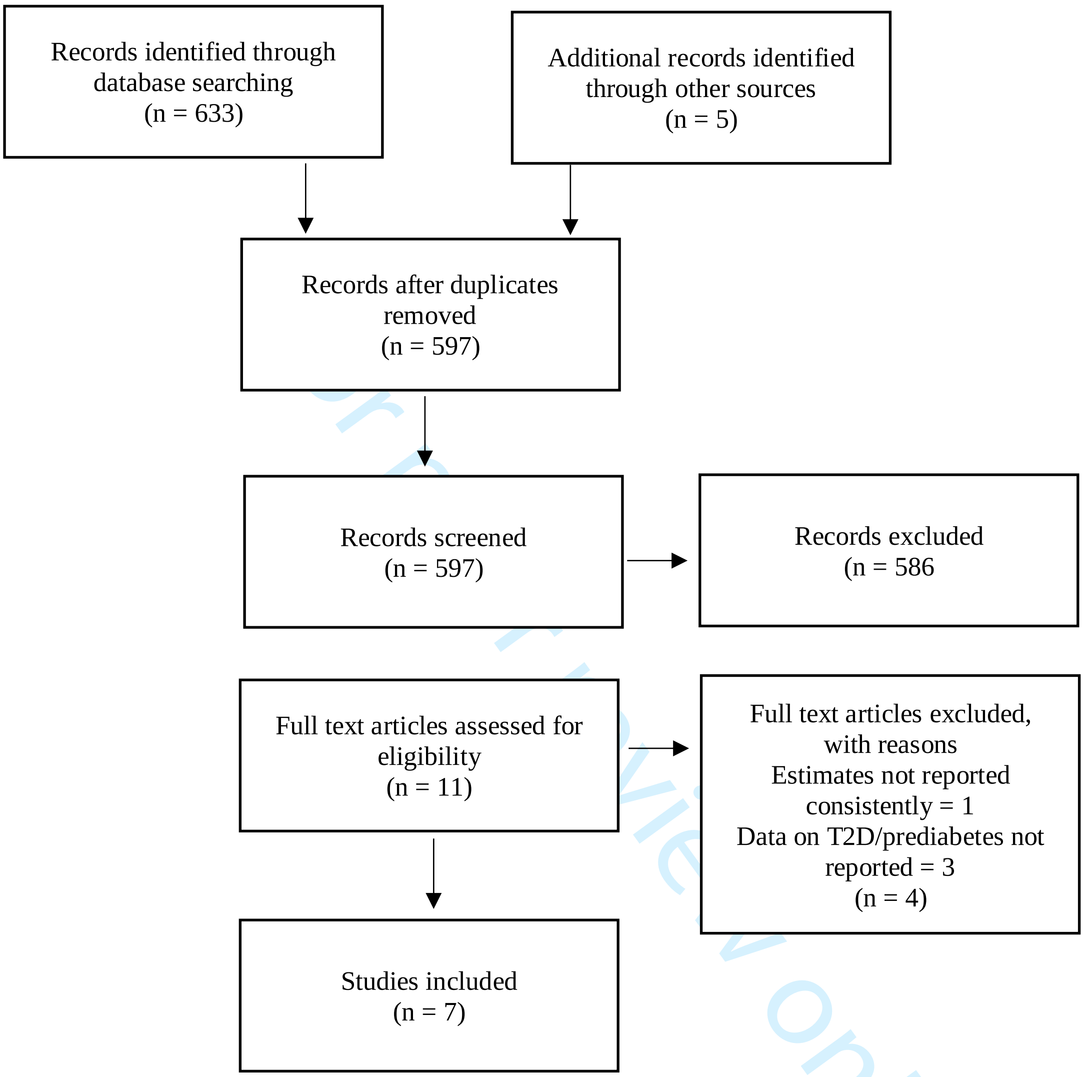

Figure 2 - Review flowchart of Markov chain models

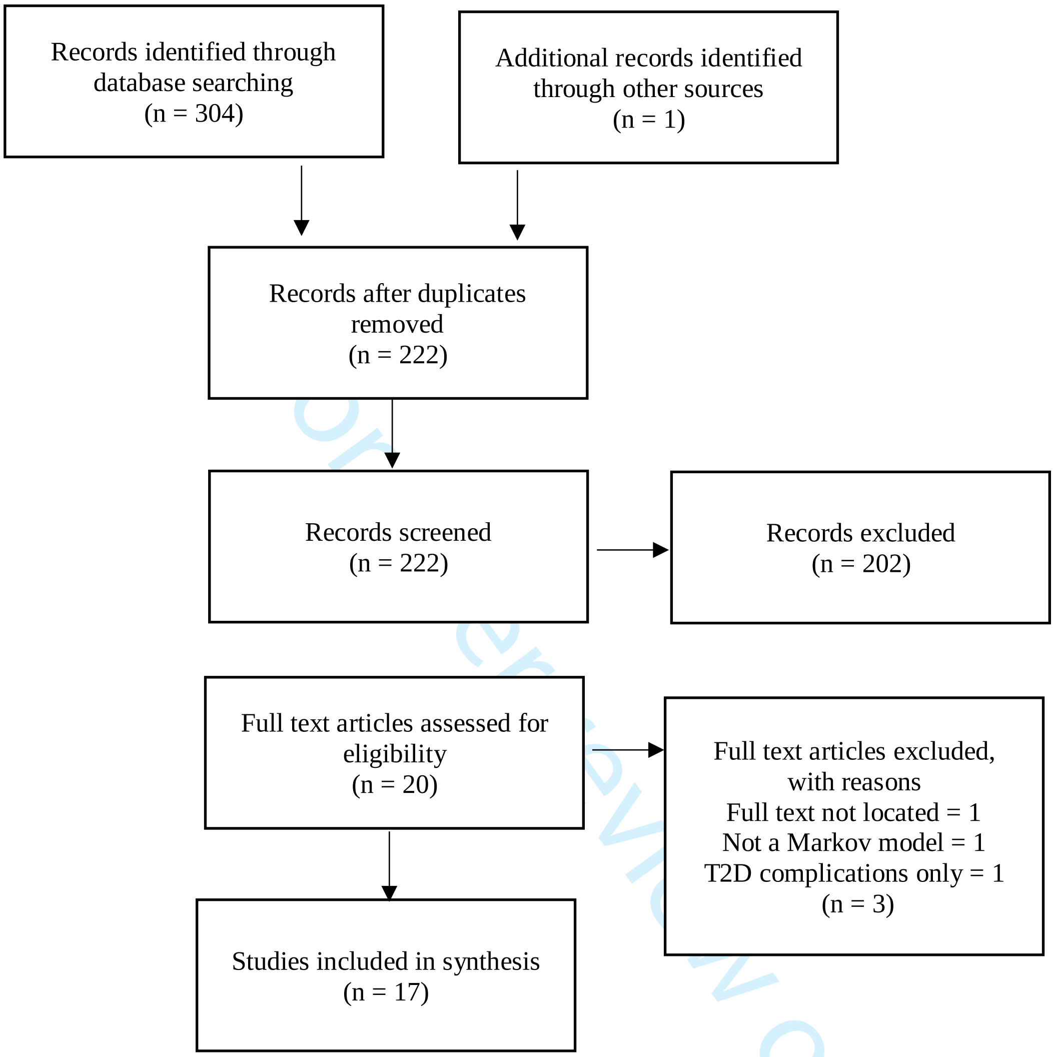

Figure 3 - Our Markov chain models

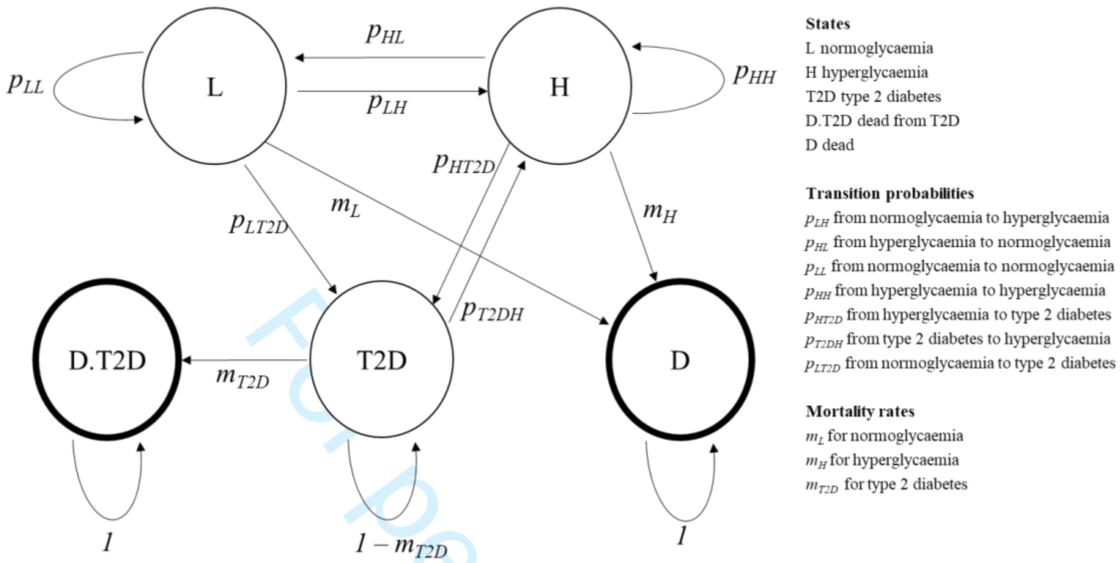

For peer review only

Figure 4: Projections of true diabetes prevalence in England: 2005 to 2035

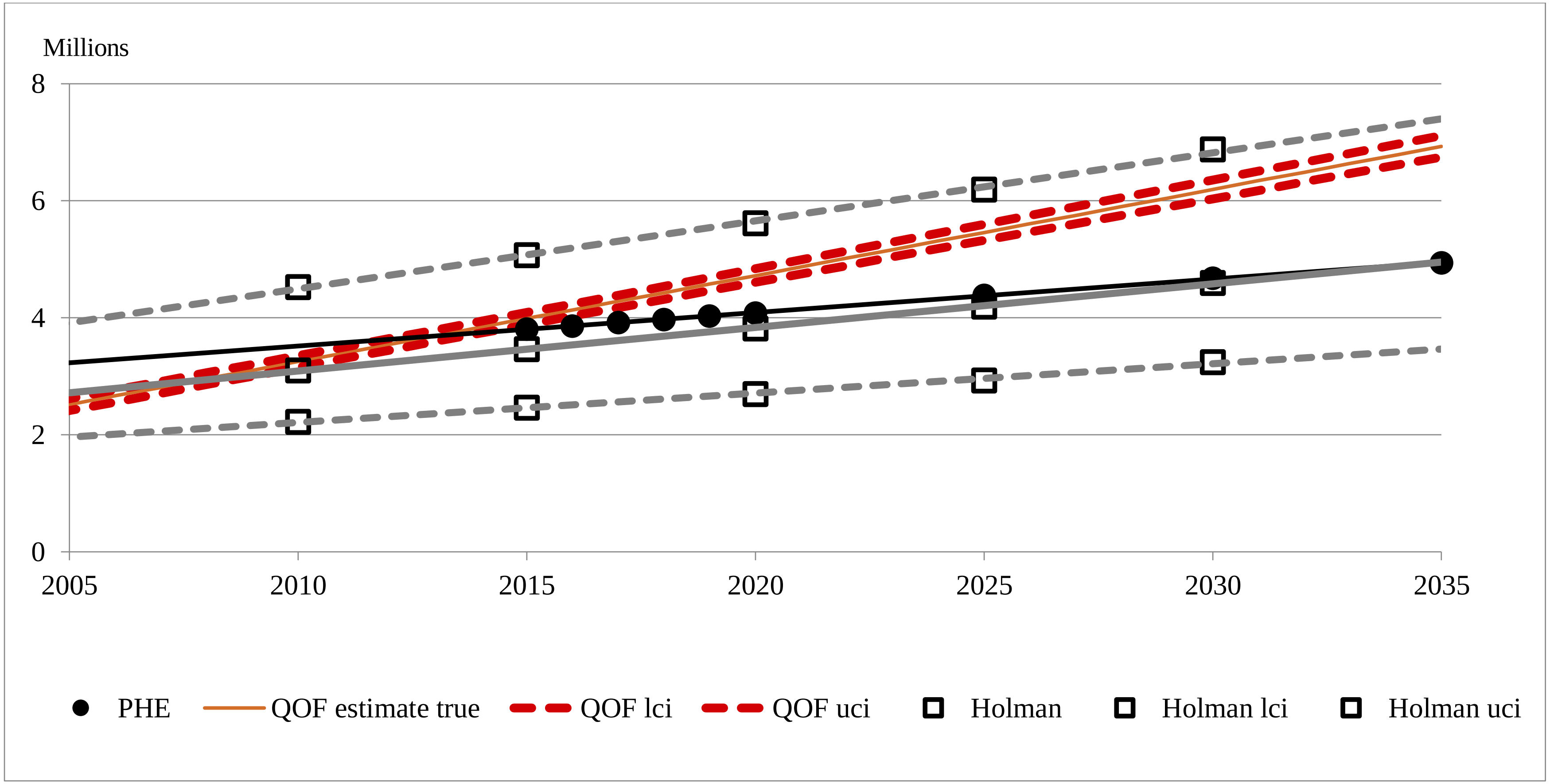

Figure 5 - Projections of the true prevalence of T2D in England: 2015 to 2035

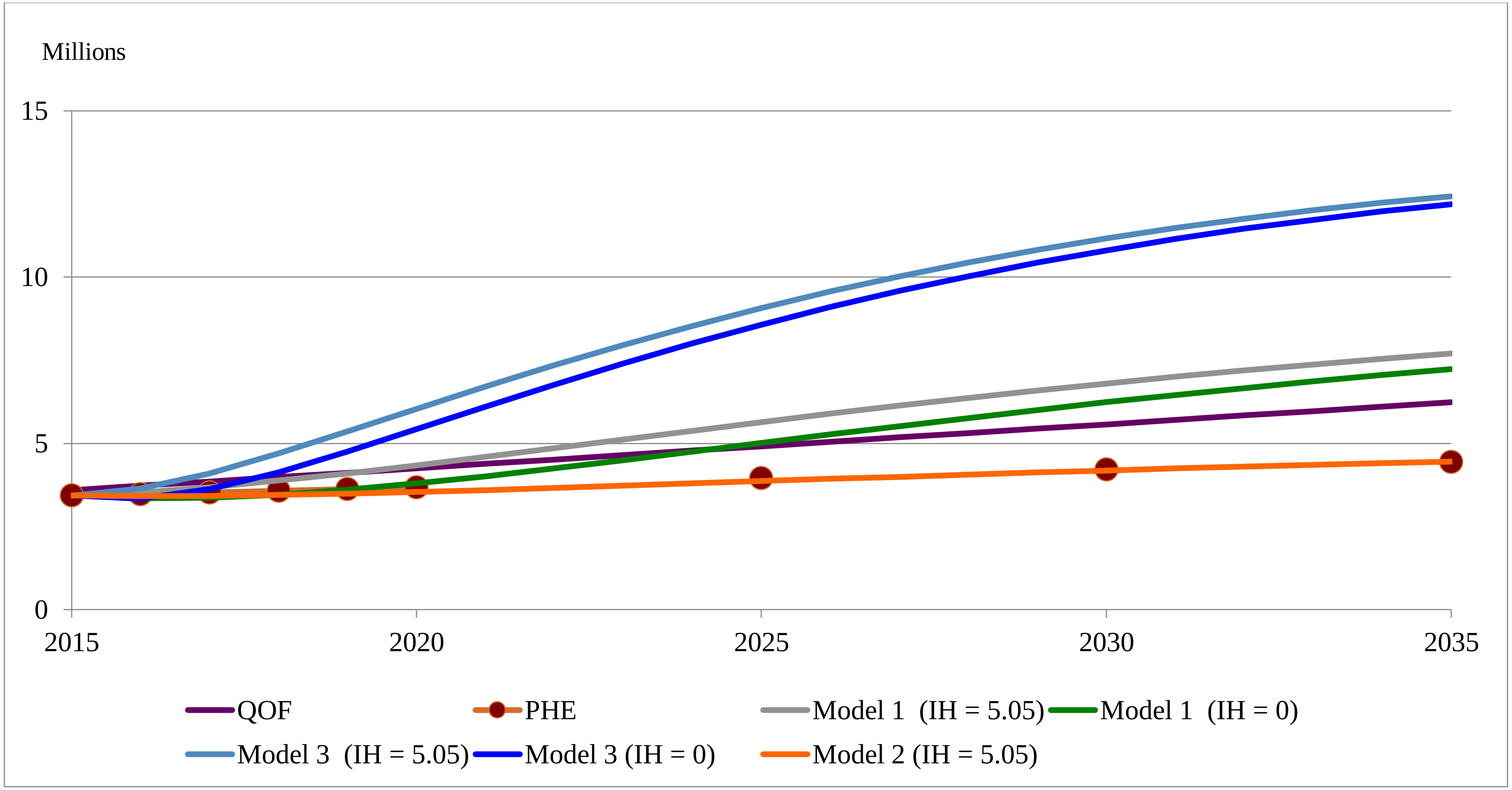

Appendix 1: Tables giving details of models

Index

Table 1.1: Search strategy of the rapid reviews

Table 1.2: Measures of intermediate hyperglycaemia used in Markov chain models

Table 1.3: Data sources of estimates used by our Markov Chain models

Table 1.4: Ratios used for comparing different estimates

Table 1.5: The three sets of transition probabilities used in different models

For peer review only

Table 1.1: Search strategy of the rapid reviews

**Epidemiological models**	
**Web of Science**	from All Databases<br>You searched for: TOPIC: ("diabet*" OR "type 2 diabetes" OR "diabetes mellitus") AND TITLE: ("Engl*" or "United Kingdom" or "UK") AND TOPIC: ("model" or "simulation" or "project*") AND TOPIC: ("epidemiolog*" or "prevalence" or "incidence" or "trend*") NOT TITLE: ("child*")<br>Refined by: LANGUAGES: (ENGLISH)<br>Timespan: All years.<br>Search language=Auto
**PubMed**	(((((("diabet*" OR "type 2 diabetes" OR "diabetes mellitus")) AND ("Engl*" OR "UK" OR "United Kingdom")) AND ("model" OR "simulation" OR "project*")) AND ("epidemiolog*" OR "prevalence" OR "incidence" OR "trend*")) NOT "child*" AND Humans[Mesh]) AND Humans[Mesh] AND English[lang] AND (Humans[Mesh] AND English[lang])
**Markov chain models**	
**Web of science**	TITLE: ("diabet*" OR "type 2 diabetes" OR "diabetes mellitus" or "pre-diabetes" or "prediabetes") AND TITLE: ("economic evaluation" or "cost-effectiveness" or "cost effectiveness" or "cost-utility" or "cost utility") AND TOPIC: ("Markov") NOT TOPIC: ("child*")<br>Refined by: LANGUAGES: (ENGLISH)<br>Timespan: All years.<br>Search language=Auto
**PubMed**	**((("diabet*" OR "type 2 diabetes" OR "diabetes mellitus" OR "prediabetes" OR "pre-**diabetes") AND ("economic evaluation" OR "cost-effectiveness" OR "cost effectiveness" OR "cost-utility" OR "cost utility")) AND "Markov" NOT "child") AND ("humans"[MeSH Terms] AND English[lang])

For peer review only - http://bmjopen.bmj.com/site/about/guidelines.xhtml

Table 1.2: Measures of intermediate hyperglycaemia used in Markov chain models

Measure of intermediate hyperglycaemia	Definition
Impaired fasting glucose (IFG)	• Diagnosed with an Oral glucose tolerance test (OGTT) performed after an overnight fast<br>• Defined by a fasting plasma glucose (FPG) concentration of<br>○ 5.6-6.9 mmol/L according to American Diabetes Association (ADA)[1]<br>○ 6.0-6.9 mmol/L according to the World Health Organization (WHO)[2]
Impaired glucose tolerance (IGT)	• Diagnosed with a 2-hour glucose tolerance test (2hrGTT), i.e. a blood test performed 2 hours after a 75-g glucose load<br>• Defined by 2-h plasma glucose concentration of<br>○ 7.8-11 mmol/L according to to American Diabetes Association (ADA)[1]<br>○ 7-11 mmol/L according to the World Health Organization (WHO)[2]
Glycated Haemoglobin (HbA1c)	• Diagnosed with the A1c test, measuring the average blood glucose over 2-3 months<br>• Defined by A1c concentration of<br>○ 39-47 mmol/mol (5.7-6.4%) according to to American Diabetes Association (ADA)[1]<br>○ 42-47 mmol/mol (6.0-6.4%) according to the World Health Organization (WHO)[3]

For peer review only

Table 1.3: Data sources of estimates used by our Markov Chain models

Estimate	Year(s)	Source
Estimated prevalence of intermediate hyperglycaemia (based on HbA1c)	2015	Public Health England[4]
Estimated prevalence of diabetes (both types)	2015	Public Health England[5]
Estimated prevalence of normoglycaemia: residual of the population for 2015	2015	Office of National Statistics[6]
Age distributions for those with intermediate hyperglycaemia & diabetes	Five years of combined data from 2009 to 2013	Health Surveys for England (HSE)[7]
Mortality rates by age	2015	Office of National Statistics[6]
Hazard ratios for those with diabetes & T2D	2015-16	National Diabetes Audit[8]
Hazard ratios for those for those with intermediate hyperglycaemia	Various years	Systematic review[9]

For peer review only

For peer review only - http://bmjopen.bmj.com/site/about/guidelines.xhtml

Table 1.4: Ratios used for comparing different estimates

Numerator	Denominator	Ratio	Sources
Diagnosed prevalence of diabetes	True prevalence of diabetes	75%	[10,11]
Prevalence of T2D	Prevalence of diabetes	90%	[12]
English population aged 20 to 79 in 2030 & 2035	UK population aged 20 to 79 in 2030 & 2035	• 2030: 87%<br>• 2035: 87%	[6]
Prevalence of diabetics aged over 15 (England) in 2030 & 2035	Prevalence of diabetics aged 20 to 79 (England) in 2030 & 2035	• 2030: 128%<br>• 2035: 129%	[7]
Prevalence of diabetics aged over 15 in England in 2030 & 2035	Prevalence of diabetics aged over 20 in UK in 2030 & 2035	• 2030: 0.87*1.28 = 111%<br>• 2035: 0.87*1.29 = 113%	[6, 7]

For peer review only

Table 1.5: The three sets of transition probabilities used in different models

	Model 1*	Model 2**	Model 3***
Normoglycaemia – Normoglycaemia	0.925	0.925	0.831
Normoglycaemia – Intermediate hyperglycaemia	0.069	0.069	0.163
Normoglycaemia – T2D	0.000	0.000	0.000
Normoglycaemia – Dead	0.006	0.006	0.006
Totals	*1.000*	*1.000*	*1.000*
Intermediate hyperglycaemia -Intermediate hyperglycaemia	0.856	0.878	0.754
Intermediate hyperglycaemia- Normoglycaemia	0.090	0.090	0.162
Intermediate hyperglycaemia – T2D	0.036	0.013	0.060
Intermediate hyperglycaemia – Dead	0.019	0.019	0.023
Totals	*1.000*	*1.000*	*1.000*
T2D-T2D	0.977	0.977	0.974
T2D – Normoglycaemia	0.000	0.000	0.000
T2D- Intermediate hyperglycaemia	0.000	0.000	0.005
T2D – Dead	0.023	0.023	0.022
Totals	*1.000*	*1.000*	*1.000*

Notes:
* Model 1is based on the transition probabilities from Roberts et al[13]for HbA1c.
** Model 2 is based on Model 1 modified to generate the PHE projections of the prevalence of T2D:the transition probability from intermediate hyperglycaemia to T2D of Model 2 (0.013) is a third of that of Model 1 (0.036); and has a corresponding increase in the transition probability of remaining as intermediate hyperglycaemia (0.836 to 0.878).
*** Model 3 is based on the transition probabilities from Neuman et al[14] for IGT.

References

1 American Diabetes Association (ADA). 2. Classification and Diagnosis of Diabetes: Standards of Medical Care in Diabetes. *Diabetes Care* 2018;**41**:S13–27.

2 World Health Organization (WHO). Definition and diagnosis of diabetes mellitus and intermediate hyperglycemia : report of a WHO/IDF consultation. Geneva (CH): 2006.

3 World Health Organization (WHO). Use of glycated haemoglobin (HbA1c) in the diagnosis of diabetes mellitus. 2011.

4 Public Health England - National Cardiovascular Intelligence Network. Prevalence estimates of non-diabetic hyperglycaemia. 2015.

5 National Cardiovascular Intelligence Network (NCVIN) - Public Health England. Prevalence estimates of diabetes. 2016.

6 Office for National Statistics. 2014-based National Population Projections. 2015.

7 National Cardiovascular Intelligence Network (NCVIN)- Public Health England. NHS Diabetes Prevention Programme (NHS DPP) Non-diabetic hyperglycaemia. 2015.

8 Healthcare Quality Improvement Partnership (HQIP). National Diabetes Audit, 2015-16 Report 2: Complications and Mortality. 2017.

9 Huang Y, Cai X, Mai W, *et al.* Association between prediabetes and risk of cardiovascular disease and all cause mortality: systematic review and

For peer review only - http://bmjopen.bmj.com/site/about/guidelines.xhtml

meta-analysis. *BMJ* 2016;**355**:i5953. doi:10.1136/bmj.i5953

10 Holman N, Forouhi NG, Goyder E, *et al.* The Association of Public Health Observatories (APHO) Diabetes Prevalence Model: Estimates of total diabetes prevalence for England, 2010-2030. *Diabet Med* 2011;**28**:575–82. doi:10.1111/j.1464-5491.2010.03216.x

11 Moody A, Cowley G, Fat LN, *et al.* Social inequalities in prevalence of diagnosed and undiagnosed diabetes and impaired glucose regulation in participants in the Health Surveys for England series. *BMJ Open* 2016;**6**:e010155. doi:10.1136/BMJOPEN-2015-010155

12 Gatineau M, Hancock C, Holman N, *et al.* Adult obesity and type 2 diabetes. London, UK: 2014.

13 Roberts S, Craig D, Adler A, *et al.* Economic evaluation of type 2 diabetes prevention programmes: Markov model of low- and high-intensity lifestyle programmes and metformin in participants with different categories of intermediate hyperglycaemia. *BMC Med* 2018;**16**:16. doi:10.1186/s12916-017-0984-4

14 Neumann A, Lindholm L, Norberg M, *et al.* The cost-effectiveness of interventions targeting lifestyle change for the prevention of diabetes in a Swedish primary care and community based prevention program. *Eur J Heal Econ* 2017;**18**:905–19. doi:10.1007/s10198-016-0851-9

For peer review only

For peer review only - http://bmjopen.bmj.com/site/about/guidelines.xhtml

Appendix 2: Rapid Reviews of Epidemiological & Markov chain models

Table 2.1 gives the search strategies for the review & Table 2.2 gives details of our rapid review of Markov chain models.

Table 2.1: Search strategies

**Web of science**	TITLE: ("diabet*" OR "type 2 diabetes" OR "diabetes mellitus" or "pre-diabetes" or "prediabetes") & TITLE: ("economic evaluation" or "cost-effectiveness" or "cost effectiveness" or "cost-utility" or "cost utility") NOT TOPIC: ("child*" or "pediatric" or "paediatric") NOT TOPIC: ("type 1 diabetes") & TOPIC: ("markov")<br>Refined by: LANGUAGES: (ENGLISH)<br>Timespan: All years.<br>Search language=Auto
**PubMed**	(((("diabet*"[All Fields] OR "type 2 diabetes"[All Fields] OR "diabetes mellitus"[All Fields] OR "pre-diabetes"[All Fields] OR "prediabetes"[All Fields]) & ("economic evaluation"[All Fields] OR "cost-effectiveness"[All Fields] OR "cost effectiveness"[All Fields] OR "cost-utility"[All Fields] OR "cost utility"[All Fields])) & "markov"[All Fields]) NOT ("child*"[All Fields] OR "pediatric"[All Fields] OR "paediatric"[All Fields])) NOT "type 1 diabetes"[All Fields] & ("humans"[MeSH Terms] & English[lang])

For peer review only

For peer review only - http://bmjopen.bmj.com/site/about/guidelines.xhtml

Table 2.2: Details of our rapid review of Markov chain models

Author	Country	Risk measure *	Objectives	Model description	Population modelled	Outcomes	Number of cases with no intervention	Sensitivity analysis	Model validation
Caro et al, 2004 [1]	Canada	IGT	To compare health & economic outcomes of acarbose, an intensive lifestyle modification programme, metformin or no intervention to prevent progression to diabetes	A Markov model to simulate long-term outcomes in a cohort of patients with IH under each of four treatment strategies. The cohort is followed for a 10- year period in the base case analyses. The model cycles over 6-month periods. Four main states were considered: normoglycaemia (NG), intermediate hyperglycaemia (IH) Type 2 Diabetes (T2D) & death. Patients who revert to NG may develop IH again, while patients who develop diabetes are assumed to remain in that state until death.	Cohort of patients with IH. For base case, patient characteristics were taken from STOP-NIDDM trial. Just over half of patients in that trial were male, & mean age at start of the trial was 54.5 years	No of patients transitioning to T2D<br>No who reverted & remained NG<br>Life expectancy<br>Years free of T2D	For a cohort of 1000 patients, over course of 10 years, 542 untreated patients with IH are expected to develop diabetes, while 242 will have returned to NG	Performed, results for base case not reported	Not reported
Chen et al, 2001[2]	Taiwan	NA	To develop natural history of T2D<br>To quantify efficacy of early detection of T2D in slowing or reducing progression of complications<br>To evaluate effect of inter-screening interval & age at start of screening on slowing/reducing progression of complications or deaths<br>To compare cost & effectiveness of a screening regime<br>To assess cost–effectiveness of T2D screening by age-specific groups & different inter-screening interval	A Markov model to simulate natural history of T2D from normal, onset, clinical complications, deaths. Disease progression modules from onset of T2D to complications include three parts: Retinopathy, Nephropathy, & Neuropathy.	Hypothetical cohort with 30,000 adults aged over 30	Life-years gained<br>QALYs	Not reported	Not reported	Not reported
Gillies et al, 2008[3]	UK	IGT	To compare potential screening strategies, & subsequent interventions, for prevention & treatment of T2D<br>(a) screening for T2D to enable early detection & treatment<br>(b) screening for T2D & impaired glucose tolerance, intervening with lifestyle interventions in those with a diagnosis of impaired glucose tolerance<br>(c) as for (b) but with pharmacological interventions<br>(d) no screening	Hybrid model consists of a decision tree & a Markov model<br>The decision tree comprises three main arms, representing no screening, screening for undiagnosed T2D, & screening for impaired glucose tolerance & undiagnosed diabetes, with either lifestyle or pharmacological interventions applied in those with impaired glucose tolerance<br><br>The Markov model consists of seven states: normal glucose tolerance, undiagnosed impaired glucose tolerance, diagnosed impaired glucose tolerance, death, & three states for people with diabetes (undiagnosed, diagnosed clinically, or diagnosed through screening, either from a screening test or because they are diagnosed with impaired glucose tolerance initially & hence enter a surveillance programme)<br>Each model cycle represents one year & the model is run for a time horizon of 50 years.	Hypothetical population, aged 45 at time of screening, with above average risk of diabetes	Clinical & cost outcomes	Not reported	Performed, results reported	Not reported

For peer review only

Author	Country	Risk measure *	Objectives	Model description	Population modelled	Outcomes	Number of cases with no intervention	Sensitivity analysis	Model validation
Herman et al, 2005[4]	USA	IGT	To estimate lifetime cost–utility of the DPP interventions.	Markov model assesses progression from IH to onset of diabetes to clinically diagnosed diabetes to diabetes with complications & death by using a lifetime simulation model. Description of the model reported elsewhere.	Members of the DPP cohort 25 years of age or older with impaired glucose tolerance	Progression of disease<br>Costs<br>Quality of life	If the entire DPP cohort were treated with the placebo intervention, approximately 50% of individuals would develop diabetes within 7 years. Over a lifetime conversion rate from IH to T2D is 82.8%	Performed, results reported	Not reported
Ikeda et al, 2010[5]	Japan	IGT	To estimate cost-effectiveness of administering voglibose, in addition to standard care of diet & exercise, compared with standard care alone for high-risk Japanese patients with impaired glucose tolerance	Markov model consisting of five stages: normal glucose tolerance, IH, T2D, dialysis & death	IH cohort, mean age 56, corresponding to the average age in the voglibose clinical trial population	Long-term costs<br>Life expectancy<br>Cost effectiveness	Not reported	Performed, results reported	Not reported
Johansson et al, 2009 [6]	Sweden	FPG	To estimate cost-effectiveness of a community-based program promoting general population lifestyle changes to prevent diabetes.	Markov model constructed to reflect metabolic syndrome, covers adults, with termination age set at 85 years, after which no further health effects or costs are accumulated. Model is fully described elsewhere.	At high risk population aged 36–56 years at baseline	Costs<br>QALYs	Not reported	Performed, results reported	Not reported
Liu et al, 2013[7]	China	IGT	To estimate clinical & economic outcomes of screening for undiagnosed diabetes & impaired glucose tolerance (IH), followed by the implementation of lifestyle intervention in those with IH.	Hybrid decision tree Markov model. The decision tree included five arms representing five scenarios. The first three scenarios involved screening for undiagnosed diabetes & IH followed by one of three active lifestyle interventions (diet, exercise or duo-intervention), which were applied to the IH subjects. The fourth scenario involved screening for undiagnosed diabetes & IH, without formal lifestyle interventions. The fifth scenario involved control group with no screening or intervention.<br>The decision tree used positive screening rates & the prevalence of diabetes & IH in reference population to determine how many individuals started in each state of the Markov models. Each Markov model consisted of eight main health states: IH, normal glucose tolerance, onset of diabetes, four diabetes complication states & death.<br>The Markov models ran for a time horizon of 40 years, & each of the model cycles represented 1 year. Separate simulations with different incidence rates of diabetes, mortality rates & health utilities were performed for the diabetes prevention programmes or for the control starting at 25, 40 & 60 years, respectively.	A representative sample of Chinese adults aged 25 years & above	Remaining survival years<br>QALYs per subject with diabetes or IH<br>Life-years gained before onset of diabetes or before onset of any complication per subject with IH<br>Cost per subject for prevention strategies or control at different initiation ages.	Not reported	Performed, results reported.	Performed, not reported
Neumann et al, 2011 [8]	Germany	IGT	To investigate long-term cost-effectiveness of lifestyle intervention programmes for the prevention of T2D	Four-state Markov modelling with a probabilistic cohort analysis : NG, IH, diagnosed T2D, or death.<br>A one-year cycle length & a lifetime time horizon are applied.	Cohort, at baseline 16% of individuals having IH, 84% NG & no one T2D.	Cost per quality-adjusted life year (QALY)	Not reported	Performed, results reported	Not reported
Neumann	Sweden	IFG	To estimate cost-	The model consisted of	With IH (details not	QALY	Not reported	Performed,	Not reported

For peer review only

Author	Country	Risk measure *	Objectives	Model description	Population modelled	Outcomes	Number of cases with no intervention	Sensitivity analysis	Model validation
et al, 2017 [9]		IGT	effectiveness of a T2D prevention initiative targeting weight reduction, increased physical activity & healthier diet in persons in pre-diabetic states by comparing a hypothetical intervention versus no intervention in a Swedish setting.	six different, mutually exclusive states: NG, IH (IGT & IGT), T2D & death. The length of one cycle was 1 year. A lifetime horizon was applied. As it was assumed that 1 year was too short to develop T2D directly from NG, this transition was not possible. Hence, all hypothetical persons must have developed any of the three pre-diabetic states before the development of T2D.	reported) based on the Vasterbotten Intervention Program (VIP)	Incremental cost-effectiveness ratios (ICERs)		results reported	
Palmer & Tucker, 2012 [10]	Australia	IGT	To examine long-term cost-effectiveness of the control, metformin & ILC interventions in the Diabetes Prevention Program (DPP) for a cohort of subjects at high risk of developing type 2 diabetes in an Australian healthcare setting	Semi-Markov model, with four health states: 'normal glucose regulation' (NGR) (plasma glucose concentration <5.6 mmol/L in fasting state or <7.8 mmol/L 2 h after a 75 g oral glucose load); 'impaired glucose tolerance' (IH) (fasting plasma glucose concentration 5.6–6.9 mmol/L or 7.8–11.0 mmol/L 2 h after a 75 g oral glucose load); 'type 2 diabetes' (T2D) (plasma glucose concentration at least 7.0 mmol/L or 11.1 mmol/L 2 h after a 75 g oral glucose load),'dead'. Each cycle in the model represented one year of a simulated subject's life & at the end of each cycle, subjects could remain in the same state, progress to another state or die. The simulation ran over subject lifetimes	Hypothetical cohort was defined with baseline characteristics in keeping with DPP study: mean age 50.6 years; 32.2% male; mean body mass index 34.0 kg/m2; & IH present.	Cumulative incidence Lifetime incremental direct costs Incremental costs per QALY-gained	Mean cumulative incidence (95% CI) of type 2 diabetes in the control arm , estimated at 89.7% (89.4–90.1)	Performed, results reported	Validation performed against the observed incidence in the US DPP & follow-up DPPOS trials. R2 correlation-coefficient estimated at 0.9987
Palmer et at, 2004 [11]	Australia France Germany Switzerla nd UK	IGT	To establish whether implementing active treatments used in DPP would be cost-effective in the selected countries.	Markov model consisting of 3 states: IH (as defined in the DPP), T2D & deceased. Simulated patients initially had IH & progressed at differing rates to T2D depending on treatment received. A patient lifetime horizon was used.	Hypothetical cohort of patients with IH, constructed to resemble the study population of the DPP (mean age, 50.6 years; mean body weight, 94.2 kg; mean body mass index [BMI], 34.0 kg/m2; men, 32.2%)	No of years free of T2D Percentage of patients developing T2D Life expectancy Total lifetime costs per patient	Not reported	Performed, results reported	Not reported
Roberts et al, 2018 [12]	England	IFG IGT HbA1c	To examine costs and effects of different intensity lifestyle programmes and metformin in participants with different categories of intermediate hyperglycaemia	Decision tree and Markov model (50-year horizon) to compare four approaches: (1) a low-intensity lifestyle programme based on current NICE guidance, (2) a high-intensity lifestyle programme based on the US Diabetes Prevention Program, (3) metformin, and (4) no intervention, modelled for three different types of intermediate hyperglycaemia (IFG, IGT and HbA1c).	Population with a diagnosis of intermediate hyperglycaemia (IFG, IGT, HbA1c)	Impact on an individual participant in a prevention programme: (1) discounted cumulative healthcare costs (including costs of diagnostic tests and primary and secondary care associated with the intervention, intermediate hyperglycaemia, T2DM and complications of T2DM), (2) discounted QALYs, (3) incidence of T2DM, (4) average number of years with T2DM, (5) cost-effectiveness ratios in £/QALY, and (6) incremental cost-effectiveness ratios (ICERs), in £/QALY (for non-dominated interventions). Impact of a nation-wide prevention programme: (1) discounted annual incremental costs, (2) discounted	With no intervention, 42% of the IGT population and 38% of the IFG and HbA1c population developed T2DM over 50 years.	Performed, results available	Performed against the National Diabetes Audit 2015-2016. Reported for the prevalence of T2D by age groups (55-59, 60-64, 65-69, 70-74, 75-79, 80-84, 85+)

For peer review only

Author	Country	Risk measure *	Objectives	Model description	Population modelled	Outcomes	Number of cases with no intervention	Sensitivity analysis	Model validation
						cumulative incremental costs, (3) discounted incremental costs as a percentage of the total diabetes expenditure, and (4) cumulative incidence of T2DM.			
Schaufler & Wolfe, 2010[13]	Germany	OGTT	To examine cost effectiveness of screening for T2D in Germany	Markov model to reproduce the time-discrete stochastic process using a 1 year cycle	General German population	Quality of Life (QOL)<br>Lifetime costs<br>Age at diabetes diagnosis<br>Incidence & Age at occurrence of diabetes-related complications.	Not reported	Performed, results reported	Performed, results not reported
Smith et al, 2010[14]	USA	IFG	To assessed cost-effectiveness of a modified version of the US DPP (mDPP)	Markov model with six states: risk factor negative (no diabetes), risk factor positive (enrolled in mDPP), risk factor positive (not enrolled in mDPP), stable T2D, complications, death	Cohort of 55-year-old men & women without a history of diabetes	Metabolic syndrome risk at 1 year<br>Costs<br>QALYs<br>T2D incidence	Without the mDPP, 9.6% of the cohort developed diabetes over 3 years	Performed, results for base-case not reported	Not reported
Wong et al, 2016 [15]	Hong Kong	IGT	To investigate costs & cost-effectiveness of a short message service (SMS) intervention to prevent the onset of T2D with IH..	Markov model with one-year transition cycle with four Markov states: normal glucose tolerance (NG), IH, T2D, & death.<br>Long-term modelling referred to time horizon over a 50-year period beyond the two year intervention	Cohort of individuals with prediabetes	Costs<br>QALYs	Not reported	Performed, results reported	Not reported
Zhou et al. 2005[16]	USA	IGT	To develop & validate a comprehensive computer simulation model to assess the impact of screening, prevention, & treatment strategies on T2D & its complications, comorbidities, quality of life, & cost.	Markov model with four states: NG, IH, T2D, death.	Not described	Health states<br>Utilities<br>Costs	Not reported	Not reported	Performed against data on individuals with T2D in Wisconsin, USA) from the Wisconsin Epidemiologic Study of Diabetic Retinopathy (WESDR). Results not reported.
Zhuo et al, 2012 [17]	USA	HbA1c	To examine change in cost effectiveness of diabetes-preventive interventions because of progressive 0.1% decremental reductions in the HbA1c cutoff from 6.4% to 5.5%.	Markov model reported elsewhere.	Nationally representative sample of U.S. adults (aged 18 years) from the 1999–2006 National Health & Nutrition Examination Survey (NHANES)	Cost effectiveness associated with HbA1c cutoffs was measured as cost per QALY gained	Not reported	Performed, results reported	Performed against results of 47 major clinical trials & cohort studies. Results not reported. Details of the model's validation reported elsewhere

Notes:

* Risk measures: HbA1c: Glycated Haemoglobin; IFG: Impaired fasting glucose; IGT: Impaired glucose tolerance; OGTT: Oral glucose tolerance test; FPG: Fasting plasma glucose; NG: normoglycaemia

For peer review only

References

[1] Caro JJ, Getsios D, Caro I, et al. Economic evaluation of therapeutic interventions to prevent Type 2 diabetes in Canada. Diabet Med 2004;21:1229–36. doi:10.1111/j.1464-5491.2004.01330.x

[2] Chen TH-H, Yen M-F, Tung T-H. A computer simulation model for cost–effectiveness analysis of mass screening for Type 2 diabetes mellitus. Diabetes Res Clin Pract 2001;54:37–42. doi:10.1016/S0168-8227(01)00307-2

[3] Gillies CL, Lambert PC, Abrams KR, et al. Different strategies for screening & prevention of type 2 diabetes in adults: cost effectiveness analysis. BMJ 2008;336.http://www.bmj.com/content/336/7654/1180.long

[4] Herman WH, Hoerger TJ, Brandle M, et al. The cost-effectiveness of lifestyle modification or metformin in preventing type 2 diabetes in adults with impaired glucose tolerance. Ann Intern Med 2005;142:323–32.http://www.ncbi.nlm.nih.gov/pubmed/15738451

[5] Ikeda S, Kobayashi M, Tajima N. Cost-effectiveness analysis of voglibose for prevention of type 2 diabetes mellitus in Japanese patients with impaired glucose tolerance. 2010;1:252–8. doi:10.1111/j.2040-1124.2010.00052.x

[6] Johansson P, Östenson C-G, Hilding AM, et al. A cost-effectiveness analysis of a community-based diabetes prevention program in Sweden. Int J Technol Assess Health Care 2009;25:350–8. doi:10.1017/S0266462309990079

[7] Liu X, Li C, Gong H, et al. An economic evaluation for prevention of diabetes mellitus in a developing country: a modelling study. BMC Public Health 2013;13:729. doi:10.1186/1471-2458-13-729.

For peer review only - http://bmjopen.bmj.com/site/about/guidelines.xhtml

[8] Neumann A, Schwarz P, Lindholm L. Estimating the cost-effectiveness of lifestyle intervention programmes to prevent diabetes based on an example from Germany: Markov modelling. Cost Eff Resour Alloc 2011;9:17. doi:10.1186/1478-7547-9-17

[9] Neumann A, Lindholm L, Norberg M, et al. The cost-effectiveness of interventions targeting lifestyle change for the prevention of diabetes in a Swedish primary care & community based prevention program. Eur J Heal Econ 2017;18:905–19. doi:10.1007/s10198-016-0851-9

[10] Palmer AJ, Tucker DMD, Polkinghorne KR, et al. Cost & clinical implications of diabetes prevention in an Australian setting: A long-term modeling analysis. Prim Care Diabetes 2012;6:109–21. doi:10.1016/j.pcd.2011.10.006

[11] Palmer AJ, Roze S, Valentine WJ, et al. Intensive lifestyle changes or metformin in patients with impaired glucose tolerance: Modeling the long-term health economic implications of the diabetes prevention program in Australia, France, Germany, Switzerland, & the United Kingdom. Clin Ther 2004;26:304–21. doi:10.1016/S0149-2918(04)90029-X

[12] Roberts S, Craig D, Adler A, et al. Economic evaluation of type 2 diabetes prevention programmes: Markov model of low- & high-intensity lifestyle programmes & metformin in participants with different categories of intermediate hyperglycaemia. BMC Med 2018;16:16. doi:10.1186/s12916-017-0984-4

[13] Schaufler TM, Wolff M. Cost Effectiveness of Preventive Screening Programmes for Type 2 Diabetes Mellitus in Germany. Appl Health Econ Health Policy 2010;8:191–202. doi:10.2165/11532880-000000000-00000

[14] Smith KJ, Hsu HE, Roberts MS, et al. Cost-effectiveness analysis of efforts to reduce risk of type 2 diabetes & cardiovascular disease in southwestern Pennsylvania, 2005-2007. Prev Chronic Dis 2010;7:A109.http://www.ncbi.nlm.nih.gov/pubmed/20712936

[15] Wong CKH, Jiao F-F, Siu S-C, et al. Cost-Effectiveness of a Short Message Service Intervention to Prevent Type 2 Diabetes from Impaired Glucose Tolerance. J Diabetes Res 2016;2016:1–8. doi:10.1155/2016/1219581

[16] Zhou H, Isaman DJM, Messinger S, et al. A computer simulation model of diabetes progression, quality of life, & cost. Diabetes Care 2005;28:2856–63.http://www.ncbi.nlm.nih.gov/pubmed/16306545

[17] Zhuo X, Zhang P, Selvin E, et al. Alternative HbA1c Cutoffs to Identify High-Risk Adults for Diabetes Prevention. Am J Prev Med 2012;42:374–81. doi:10.1016/j.amepre.2012.01.003

For peer review only

For peer review only - http://bmjopen.bmj.com/site/about/guidelines.xhtml
